# MPK3-mediated phosphorylation inactivates MYB4 to promote FIT-dependent iron acquisition

**DOI:** 10.1038/s44319-026-00906-1

**Published:** 2026-08-25

**Authors:** Hui Song, Guangna Chen, Jiena Xu, Jing Zhang, Shuangyao Li, Shuxun Xu, Yanjun Wu, Li Jiang, Xi Wu, Shuqing Cao

**Affiliations:** School of Food and Biological Engineering, Hefei University of Technology, Hefei, China

**Keywords:** Plant Biology, Post-translational Modifications & Proteolysis

## Abstract

Iron (Fe) is an essential micronutrient for plant growth, and its homeostasis is critically regulated by the transcription factor FIT in response to deficiency. Here, we identify the R2R3-MYB transcription factor MYB4 as a previously unknown negative regulator of Fe acquisition. MYB4 directly interacts with FIT, competing with bHLH38/39 to prevent the formation of transcriptionally active heterodimers, thereby repressing the expression of the key Fe-uptake genes *IRT1* and *FRO2*. We establish that the mitogen-activated protein kinase MPK3 acts upstream in this pathway. Under Fe-deficient conditions, MPK3 is activated and directly phosphorylates MYB4 at Serine 4, promoting its proteasome-dependent destabilization. This post-translational switch releases FIT to dimerize with bHLH partners, activating Fe uptake. Our work unveils the MPK3-MYB4-FIT signaling module as a previously unrecognized regulatory module that contributes to the dynamic control of Fe homeostasis in *Arabidopsis*.

## Synopsis

Iron (Fe) deficiency activates MPK3-mediated phosphorylation of MYB4, triggering MYB4 degradation and releasing FIT-bHLH38/39 complex activity. This phospho-switch mechanism controls Fe uptake gene expression and maintains Fe homeostasis in Arabidopsis.

- MYB4 inhibits FIT-bHLH38/39 complex formation and suppresses Fe uptake gene expression during Fe sufficiency.
- MPK3 phosphorylates MYB4 to promote its ubiquitin-dependent degradation during Fe deficiency.
- Loss of MYB4 promotes Fe acquisition and Fe deficiency tolerance through activation of the FIT pathway.
- The MPK3-MYB4-FIT module provides post-translational control of plant Fe homeostasis.

## Abstract (web summary)

Iron (Fe) deficiency activates MPK3-mediated phosphorylation of MYB4, triggering MYB4 degradation and releasing FIT-bHLH38/39 complex activity. This phospho-switch mechanism controls Fe uptake gene expression and maintains Fe homeostasis in Arabidopsis.

## Graphical abstract

**Figure:**
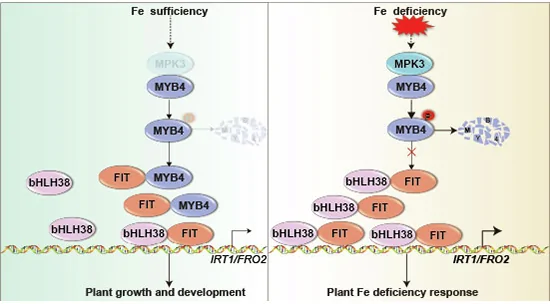

## Introduction

Iron (Fe) is one of the important nutrients necessary for plants and animals. It plays an important role in maintaining the normal physiological functions of the animal organism, participating in hematopoiesis, maintaining growth and development, and resisting infectious diseases (Hänsch and Mendel, 2009; Gao et al, 2021; Wu et al, 2023; Liang, 2022). Similarly, Fe has an essential role for plants, being involved in photosynthesis, biological nitrogen fixation, and the composition of cytochromes and non-heme ferritin in respiration and is an important substance in energy metabolism (Hänsch and Mendel, 2009; Gao et al, 2021). Fe is required for chloroplast synthesis, so plants under Fe deficiency stress lose their green color between the veins, and it is typical for young leaves to lose their green color. Because Fe availability directly affects chlorophyll biosynthesis and photosynthetic capacity, chlorophyll content serves as a widely used integrative indicator of plant Fe nutritional status (Abadía, 1992). Although the soil is rich in large amounts of Fe, the availability is extremely low, high pH and high carbonate content seriously reduces the effectiveness of Fe in the soil, leading to difficulties in plant uptake of Fe from the soil, resulting in Fe deficiency stress, which seriously affects the growth of plants and reduces crop yields (Chen and Barak, 1982; Mengel, 1994; Gao et al, 2021; Song et al, 2022). Plants have developed two major Fe deficiency adaptation mechanisms in response to Fe deficiency stress during the evolutionary process: mechanism I and mechanism II. Except for gramineous plants, which acquire Fe through mechanism II, all other plants acquire Fe from the soil through mechanism I (Liang, 2022; Cui et al, 2018; Song et al, 2022).

Fe signaling derived from Fe demand and Fe-deficient states leads to the induction of multiple clusters of co-expressed genes in the root and leaf (Ivanov et al, 2012). As is well known, transcription factors have become important checkpoints for controlling Fe homeostasis in the molecular-level study of plant Fe signal transduction (Le et al, 2016). *Arabidopsis thaliana*, a model plant for regulating Fe homeostasis through mechanism I, has a big family of basic helix-loop-helix (bHLH) transcription factors (Bailey et al, 2003). It was previously reported that under conditions of excess or adequate Fe availability, Fe acquisition is down-regulated to avoid the deleterious effects of excessive free Fe in the cells (Ivanov et al, 2014; Li et al, 2019). *Arabidopsis FIT/bHLH29* is a key transcription factor regulating Fe homeostasis and is a member of the bHLH family, which can balance the helpful and potentially detrimental roles of Fe in cells (Ling et al, 2002; Colangelo and Guerinot, 2004). Fe deficiency upregulates FIT at the transcriptional level, whereas FIT protein accumulation is tightly controlled by 26S proteasome-mediated degradation depending on the Fe status of the plant (Lingam et al, 2011; Rodríguez-Celma et al, 2019). It has been demonstrated that FIT transcription factor activity is controlled by protein–protein interactions, and that Fe-deficiency stress FIT must physically interact with subgroup Ib bHLH (bHLH38/39/100/101) transcriptional regulators to modulate the expression of the downstream targets, *IRT1* and *FRO2*, and thereby enhance plant adaptation to Fe-deficiency stress (Yuan et al, 2008; Wang et al, 2013). In addition, several critical genes have been demonstrated to be negative regulators of Fe uptake and FIT function, such as *bHLH18, bHLH19, bHLH20, bHLH25, DELLAs, MYC1, ZAT12, FBP*, and *BTSL1* (Wild et al, 2016; Le et al, 2016; Cui et al, 2018; Chen et al, 2018; Rodríguez-Celma et al, 2019; Song et al, 2022). Meanwhile, the protein kinase CIPK11 and EIN3/ EIL1 ethylene signaling transcription factors have been found to favor FIT stability and Fe acquisition (Lingam et al, 2011; Gratz et al, 2019). However, since FIT is a vital ingredient in the Fe-deficiency pathway in plants, the mechanism of action of FIT in regulating Fe homeostasis in plants under both Fe-sufficient and Fe-deficient conditions remains to be further explored.

MYB transcription factors are one of the largest families of transcription factors in plants, and it has been shown that many MYB family members function distinctly in different stresses (Palmer et al, 2013; Yan et al, 2021; Wu et al, 2024). Several studies have reported that MYB-mediated transcriptional cascade responses are associated with plant biotic and abiotic stress regulatory networks, such as pathogens (He et al, 2020), drought (Yang et al, 2022), salinity (He et al, 2012), cold (Kim et al, 2017), and heavy metal cadmium stress (Yan et al, 2021). Interestingly, the R2R3-MYB transcription factors *MYB10* and *MYB72* have been shown to be required for normal Fe uptake in plants, and the expression of MYB10 and MYB72 was significantly induced by Fe deficiency stress, whereas their expression was partially dependent on the activity of bHLH121 and FIT (Palmer et al, 2013; Zamioudis et al, 2014; Gao et al, 2024). Recently, the transcription factor *bZIP44* was reported to positively regulate the response of *Arabidopsis* to Fe-deficient stress (Wu et al, 2024). Under Fe deficiency stress, bZIP44 synergistically enhances its binding ability to the *NAS2/4* promoter by interacting with MYB10/72, thereby increasing the expression of *NAS2/4*, promoting NA synthesis, and thus enhancing plant tolerance to Fe deficiency stress (Wu et al, 2024). *MdMYB58* transcription factor was found to positively regulate apple Fe uptake and translocation by binding to the *MdMATE43* and *FRD3* promoters (Wang et al, 2018). In contrast, *MxMYB1* was found to be involved in Malus xiaojinensis Fe uptake and storage possibly as a negative regulator (Shen et al, 2008). Interactions between proteins are an important strategy in the regulatory network of plant adaptation to Fe deficiency (Lingam et al, 2011; Wild et al, 2016; Le et al, 2016; Cui et al, 2018; Chen et al, 2018; Rodríguez-Celma et al, 2019; Gratz et al, 2019; Song et al, 2022). However, there are no reports on MYB and bHLH transcription factors interacting in Fe deprivation in *Arabidopsis*.

Protein kinase-mediated phosphorylation is critical in signal transduction for plant growth and development and for defense against adverse environments (Zhang et al, 2023; Peng et al, 2024). By precisely phosphorylating critical components of the signal cascade, plants can dynamically regulate specific signal pathways depending on the situation (Zhang et al, 2023). Mitogen-activated protein kinase (MAPK), as an important component of the signal transduction pathway, is widely present in eukaryotes and plays an important role in regulating cell development and responding to external environmental stimuli (Meng et al, 2013). A recent study demonstrated that the MEKK1-MKK1/2-MPK4 cascade phosphorylates and stabilizes the zinc-finger transcription factor *STOP1*, thereby promoting aluminum resistance in *Arabidopsis* (Zhou et al, 2023). The MAPK cascade signaling system consists of three classes of protein kinases, MAPKKKs/MEKKs/MAP3Ks, MKKs/MEKs/MAP2Ks, and MAPKs (Zhang and Zhang, 2022). When cells sense signals through sensors or receptors, they activate MEKKs through various mechanisms, which then transmit signals through the MEKKs-MKKs-MAPKs cascade, followed by phosphorylation modification of downstream transcription factors or other substrates by activated MAPKs to achieve specific signal transduction (Zhang et al, 2023). There are two well-studied MAPK cascades, MEKK1-MKK1/2-MPK4 and MKK4/5-MPK3/6, which have been associated with plant resistance to adverse environments and development (Petersen et al, 2000; Teige et al, 2004; Meng et al, 2013; Pitzschke et al, 2014). Additionally, the MEKK1-MKK2-MPK4 cascade has been reported to positively regulate not only the cold stress response and freezing tolerance in plants (Zhao et al, 2017), but also involves the modulation of salt stress response (Teige et al, 2004). In recent years, the transcriptional repressor MYB15 has been shown to alter its biochemical function under cold stress by being phosphorylated by MPK6, thereby attenuating the binding ability of *MYB15* to the CBF promoter, which leads to the enhancement of CBF transcriptional activity and the increase of cold tolerance and frost tolerance in plants (Kim et al, 2017). Previous studies have shown that MPK3 and MPK6 respond to plant salt stress by phosphorylating modifications of the resistance salt-regulated genes *SOS1* and *ARR1/10/12* (Yu et al, 2010). Activated MPK3/MPK6 is involved in plant regulation of oxidative and salt stress by further phosphorylating the heat shock factor HSFA4A (Pérez-Salamó et al, 2014). Even though the function of the MAPK signaling cascade in diverse stresses has been extensively studied, whether MPK3 is involved in the FIT-mediated Fe uptake pathway via substrate phosphorylation modification remains to be discovered.

In this study, we elucidate a novel regulatory mechanism whereby the transcription factor *MYB4* acts as a negative regulator of Fe deficiency responses in *Arabidopsis*. Mechanistically, under Fe-sufficient conditions, MYB4 is moderately expressed and competes with bHLH38/39 for binding to FIT, thereby inhibiting the formation of functional FIT-bHLH38/39 heterodimers. As a result, the transcriptional activities of Fe-uptake genes *IRT1* and *FRO2* are inhibited, maintaining Fe homeostasis in plants under normal physiological conditions. Significantly, we identified post-translational modification pathways mediated by MAPK kinases: Under Fe deficiency stress, the induction of MPK3 protein expression enhances Ser4 phosphorylation of MYB4, thereby reducing MYB4 protein stability. This leads to the release of FIT, enabling its formation of functional complexes with bHLH38/39, thereby activating the expression of *IRT1* and *FRO2* and enhancing Fe uptake. Our research reveals that the MPK3-MYB4-FIT module represents an additional regulatory module contributing to the fine-tuning of plant Fe homeostasis.

## Results

### MYB4 proteins interact with FIT

**Figure 1:**
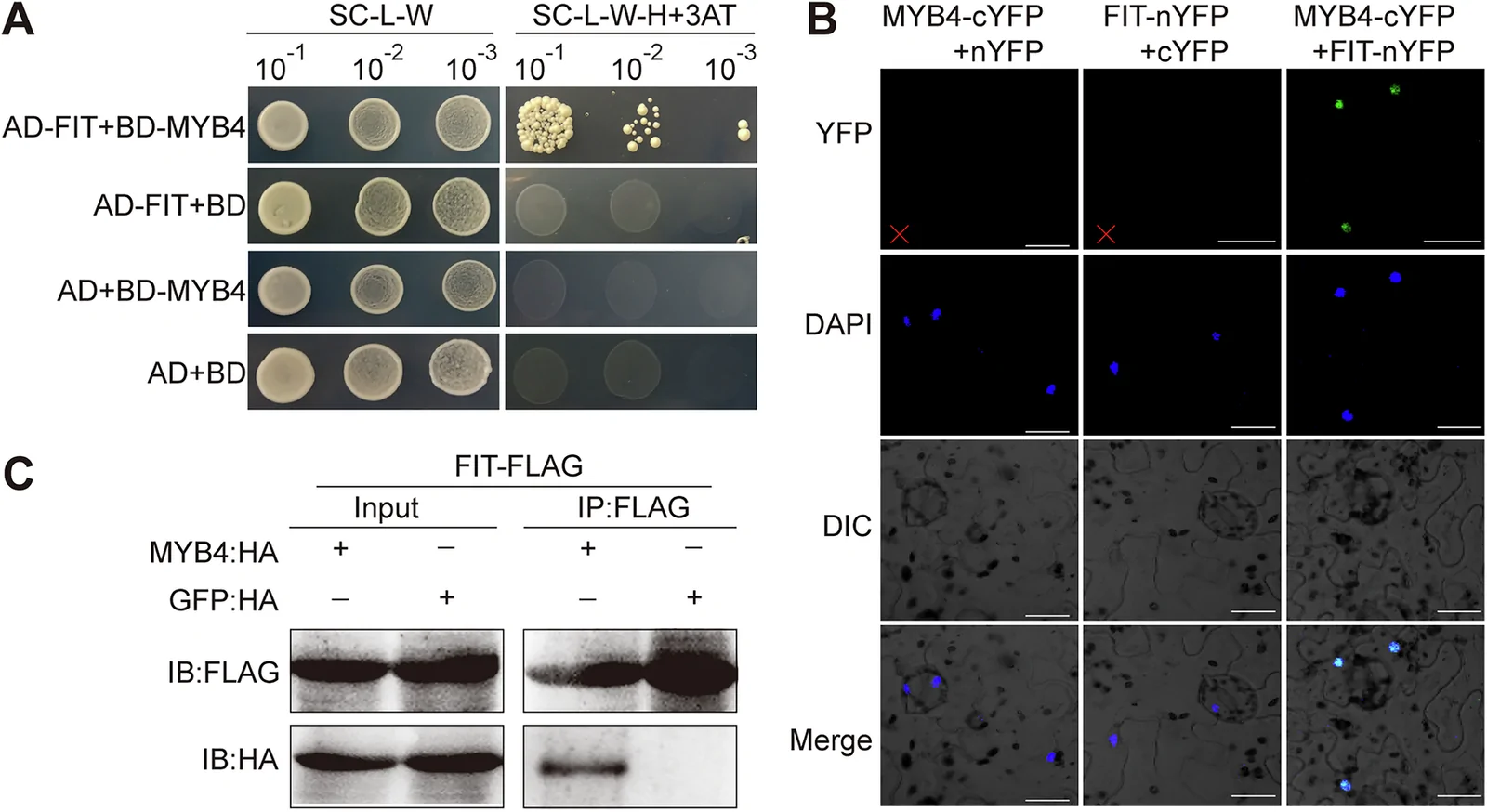
Interaction analysis of MYB4 with FIT. (**A**) Y2H assay to verify the interaction of MYB4 with FIT. (**B**) BiFC assay confirmed the interaction of FIT with MYB4 in plant cells. Scale bar:50 μm. The red marking indicates that no fluorescence signal was detected. (**C**) The co-immunoprecipitation analysis showed that FIT interacts with MYB4. Source data are available online for this figure. Source data are available online for this figure.

To elucidate the molecular mechanisms governing Fe homeostasis in plants, we conducted a yeast two-hybrid screening using a truncated version of FIT (lacking the N-terminal 99 amino acids to abolish self-activation activity) as bait. We identified MYB4 as a novel FIT-interacting transcription factor. Yeast two-hybrid assays (Y2H) confirmed a stronger interaction between MYB4 and FIT (Fig. 1A). To validate this interaction in planta, bimolecular fluorescence complementation (BiFC) assays were performed by co-expressing *35S:MYB4-cYFP* and *35S:FIT-nYFP* in *Nicotiana benthamiana* leaves. Distinct YFP fluorescence signals were detected in co-expressed tissues, while control combinations showed no signal (Fig. 1B). Co-immunoprecipitation (Co-IP) assays further showed that FIT-FLAG co-precipitated with MYB4-HA exclusively in co-expressed samples, as evidenced by western blot analysis (Fig. 1C). These results confirm the interaction between MYB4 and FIT.

### MYB4 negatively regulates Fe deficiency stress response

**Figure 2:**
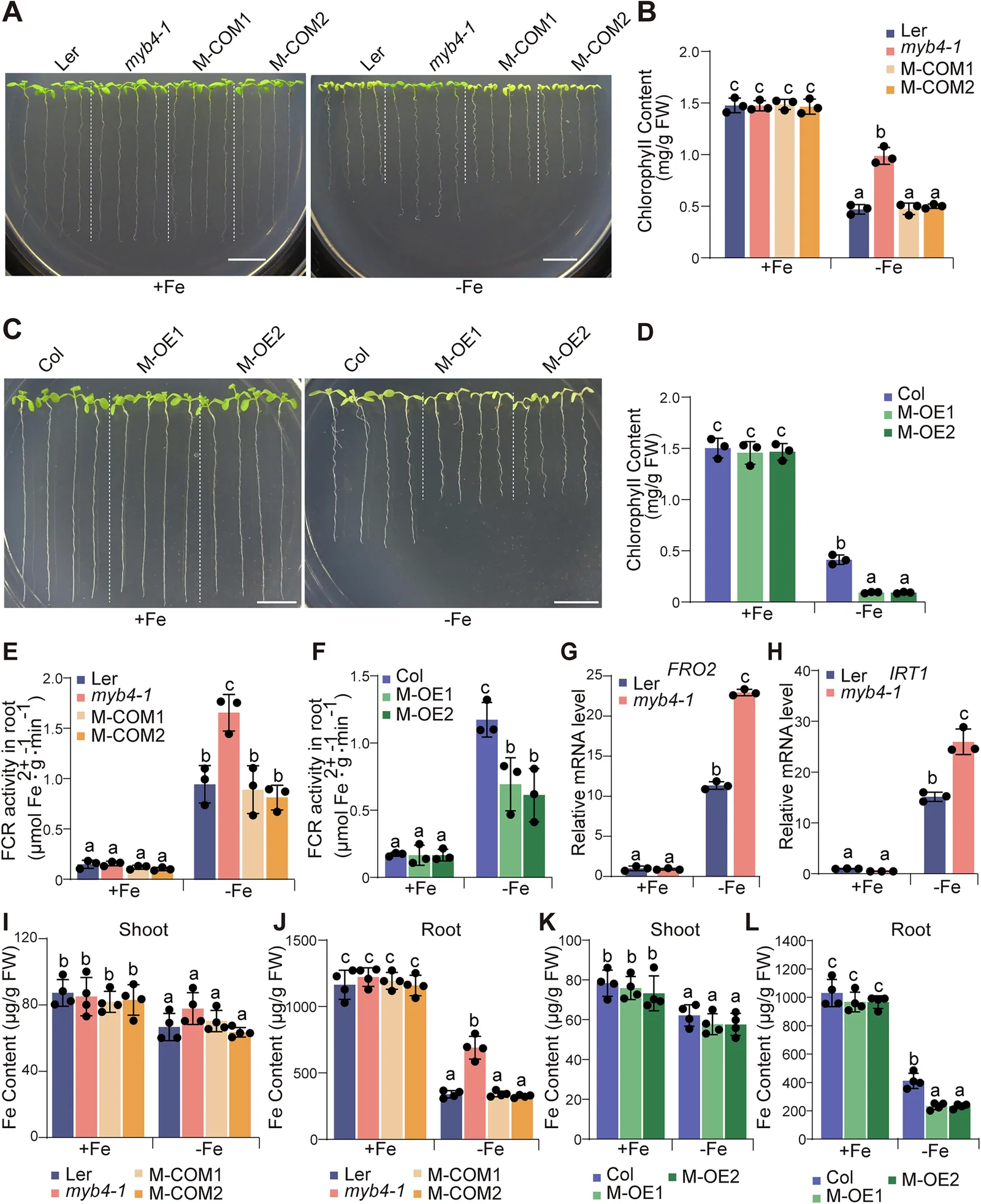
Phenotypic analysis of WT, *myb4* mutant, *MYB4-COM*, and *MYB4-OE* lines in response to Fe deficiency stress. (**A**) Fe deficiency stress tolerance analysis of Ler, *myb4-1*, and *MYB4-COM* line seedlings. Scale bar:1 cm. (**B**) The total chlorophyll content of Ler, *myb4-1*, and *MYB4-COM* under both normal and Fe deficiency stress conditions. Values shown are means ± SD (*n* = 3 biological replicates). Significance was determined using two-way ANOVA followed by Tukey’s test. *P* values >0.999 (+Fe Ler vs +Fe *myb4-1*), >0.999 (+Fe Ler vs +Fe *M-COM1*), >0.999 (+Fe Ler vs +Fe *M-COM2*), >0.999 (+Fe *myb4-1* vs +Fe *M-COM1*), >0.999 (+Fe *myb4-1* vs +Fe *M-COM2*), >0.999 (+Fe *M-COM1* vs +Fe *M-COM2*), < 0.0001 (-Fe Ler vs -Fe *myb4-1*), >0.999 (-Fe Ler vs -Fe *M-COM1*), >0.999 (-Fe Ler vs -Fe *M-COM2*), <0.0001 (-Fe *myb4-1* vs -Fe *M-COM1*), <0.0001 (-Fe *myb4-1* vs -Fe *M-COM2*), >0.999 (-Fe *M-COM1* vs -Fe *M-COM2*). (**C**) Fe deficiency stress tolerance analysis of Col and *MYB4-OE* line seedlings. Scale bar:1 cm. (**D**) The total chlorophyll content of Col and *MYB4-OE* under both normal and Fe deficiency stress conditions. Values shown are means ± SD (*n* = 3 biological replicates). Significance was determined using two-way ANOVA followed by Tukey’s test. *P* values = 0.9998 (+Fe Col vs +Fe *M-OE1*), >0.9999 (+Fe Col vs +Fe *M-OE2*), >0.9999 (+Fe *M-OE1* vs +Fe *M-OE2*), 0.002 (-Fe Col vs -Fe *M-OE1*), 0.0019 (-Fe Col vs -Fe *M-OE2*), >0.9999 (-Fe *M-OE1* vs -Fe *M-OE2*). (**E**) Root FCR activity of *Ler, myb4-1*, and *MYB4-COM*. Values shown are means ± SD (*n* = 3 biological replicates). Significance was determined using two-way ANOVA followed by Tukey’s test. *P* values >0.9999 (+Fe Ler vs +Fe *myb4-1*), >0.9999 (+Fe Ler vs +Fe *M-COM1*), 0.9998 (+Fe Ler vs +Fe *M-COM2*), >0.9999 (+Fe *myb4-1* vs +Fe *M-COM1*), 0.9995 (+Fe *myb4-1* vs +Fe *M-COM2*), >0.9999 (+Fe *M-COM1* vs +Fe *M-COM2*), 0.0001 (-Fe Ler vs -Fe *myb4-1*), 0.9996 (-Fe Ler vs -Fe *M-COM1*), 0.9186 (-Fe Ler vs -Fe *M-COM2*), <0.0001 (-Fe *myb4-1* vs -Fe *M-COM1*), <0.0001 (-Fe *myb4-1* vs -Fe *M-COM2*), 0.9951 (-Fe *M-COM1* vs -Fe *M-COM2*). (**F**) Root FCR activity of Col and *MYB4-OE* lines. Values shown are means ± SD (*n* = 3 biological replicates). Significance was determined using two-way ANOVA followed by Tukey’s test. *P* values >0.9999 (+Fe Col vs +Fe *M-OE1*), >0.9999 (+Fe Col vs +Fe *M-OE2*), >0.9999 (**+**Fe *M-OE1* vs +Fe *M-OE2*), 0.0112 (-Fe Col vs -Fe *M-OE1*), 0.0031 (-Fe Col vs -Fe *M-OE2*), 0.9643 (-Fe *M-OE1* vs -Fe *M-OE2*). (**G**,** H**) RT-qPCR analysis of *IRT1* and *FRO2* genes in Ler and *myb4-1* mutant roots under Fe deficiency stress. *ACTIN8* was used as an internal control. Values shown are means ± SD (*n* = 3 biological replicates). Significance was determined using one-way ANOVA followed by Tukey’s test. (**G**) *P* values = 0.787 (+Fe Ler vs +Fe *myb4-1*), <0.0001 (-Fe Ler vs -Fe *myb4-1*) (The following is the same order). (**H**) *P* values = 0.9645, <0.0001. (**I**–**L**) Fe content of the roots and shoots of WT, *myb4-1*, *MYB4-COM* and *MYB4-OE* lines under both normal and Fe-deficiency conditions. Values shown are means ± SD (*n* = 4 biological replicates). Significance was determined using two-way ANOVA followed by Tukey’s test. (**I**) *P* values = 0.9991 (+Fe Ler vs +Fe *myb4-1*), 0.9735 (+Fe Ler vs +Fe *M-COM1*), 0.9709 (+Fe Ler vs +Fe *M-COM2*), 0.9998 (+Fe *myb4-1* vs +Fe *M-COM1*), 0.9998 (+Fe *myb4-1* vs +Fe *M-COM2*), >0.9999 (+Fe *M-COM1* vs +Fe *COM2*), 0.5662 (-Fe Ler vs -Fe *myb4-1*), 0.9994 (-Fe Ler vs -Fe *M-COM1*), 0.9938 (-Fe Ler vs -Fe *M-COM2*), 0.9597 (-Fe *myb4-1* vs -Fe *M-COM1*), 0.1855 (-Fe *myb4-1* vs -Fe *M-COM2*), 0.8978 (-Fe *M- COM1* vs -Fe *COM2*) (The following is the same order). (**J**) *P* values = 0.9125, 0.9982, >0.9999, 0.9983, 0.872, 0.995, <0.0001, >0.9999, >0.9999, <0.0001, <0.0001, >0.9999. (**K**) *P* values = 0.9936 (+Fe Col vs +Fe *M-OE1*), 0.8583 (+Fe Col vs +Fe *M-OE2*), 0.9897 (+Fe *M-OE1* vs +Fe *M-OE2*), 0.9145 (-Fe Col vs -Fe *M-OE1*), 0.9009 (-Fe Col vs -Fe *M-OE2*), >0.9999 (-Fe *M-OE1* vs -Fe *M-OE2*) (The following is the same order). (**L**) *P* values = 0.6357, 0.623, >0.9999, 0.0038, 0.0038, >0.9999. Source data are available online for this figure.

**Figure EV1:**
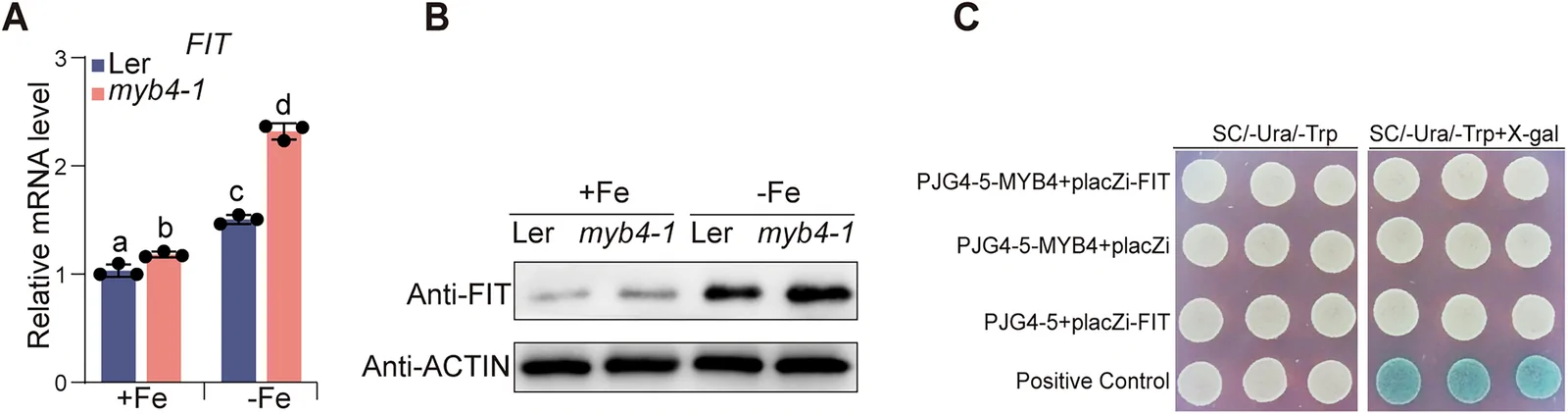
Quantitative analysis of *FIT* transcript and protein levels in WT and *myb4-1* plants under Fe-deficiency stress. (**A**) Expression levels of *FIT* in WT and *myb4-1* plants were detected by RT-qPCR under Fe deficiency conditions. Arabidopsis seedlings were grown under both normal and Fe-deficient conditions, and roots were harvested for RNA extraction followed by RT-qPCR analysis. *ACTIN8* was used as an internal control. Values shown are means ± SD (*n* = 3 biological replicates). Significance was determined using two-way ANOVA followed by Tukey’s. *P* values = 0.0352 (+Fe Ler vs +Fe *myb4-1*), <0.0001 (+Fe Ler vs -Fe Ler), <0.0001 (+Fe Ler vs -Fe *myb4-1*), 0.003 (+Fe *myb4-1* vs -Fe Ler), <0.0001 (+Fe *myb4-1* vs-Fe *myb4-1*), <0.0001 (-Fe Ler vs -Fe *myb4-1)*. (**B**) FIT protein levels in WT and *myb4-1* mutant plants were analyzed by Western blotting under Fe-deficiency conditions. Protein extracts from treated seedlings were analyzed by 10% SDS-PAGE and Western blotting assay using an anti-FIT antibody (upper panel). Total protein stained with Coomassie (lower panel), bright blue was used as the control. (**C**) MYB4 cannot interact with the *FIT* promoter in yeast. Y1H assays were performed on synthetic dropout (SD) medium lacking tryptophan and uracil (SD/-Trp/-Ura) to select yeast transformants carrying both bait and prey plasmids. Protein-DNA interactions were further assessed on SD/-Trp/-Ura medium supplemented with X-Gal (SD/-Trp/-Ura+X-Gal). The empty PJG4-5 and placZi prey vector is used as a negative control.

Given the demonstrated physical interaction between MYB4 and FIT, we investigated the role of MYB4 in regulating plant Fe-deficiency stress responses. Phenotypic analysis revealed that *myb4-1* mutants exhibited wild-type growth under Fe-sufficient conditions but showed significantly enhanced tolerance to Fe-deficiency stress, accompanied by elevated chlorophyll levels and Fe (III)-chelate reductase (FCR) activity compared to wild-type (Ler) plants (Fig. 2A,B,E). Genetic complementation (*MYB4-COM* lines) fully restored Fe-deficiency stress sensitivity to wild-type levels (Fig. 2A,B,E), confirming the causal relationship between MYB4 loss-of-function and enhanced Fe-deficiency stress tolerance. Conversely, *MYB4-OE* overexpression lines in Col background displayed hypersensitivity to Fe deficiency stress with reduced chlorophyll accumulation and FCR activity (Fig. 2C,D,F), establishing *MYB4* as a negative regulator of Fe-deficiency adaptation. We further analyzed the expression levels of *IRT1* and *FRO2* in Ler and *myb4-1* mutant plants under Fe deficiency stress. The results revealed that the expression levels of *IRT1* and *FRO2* in the *myb4-1* were significantly increased under Fe deficiency stress compared with Ler (Fig. 2G,H). Furthermore, as *MYB4* is a transcriptional regulatory factor, we also examined the expression levels of *FIT* at both the transcriptional and protein levels in the *myb4-1* mutant under Fe deficiency conditions (Fig. EV1A,B). We found that the protein and transcriptional levels of FIT were elevated in the *myb4-1* mutant compared to the Ler under Fe deficiency conditions. Furthermore, we conducted yeast one-hybrid assays and found that MYB4 does not bind to the *FIT* promoter (Fig. EV1C), indicating that MYB4 cannot directly regulate the transcription of *FIT*. These results suggest that under Fe deficiency stress, MYB4 may inhibit the expression of *IRT1* and *FRO2*. The elevated levels of FIT transcripts and proteins observed in the *myb4-1* mutant are likely an indirect consequence of altered Fe homeostasis and enhanced Fe deficiency signaling, rather than a direct regulation of *FIT* expression by MYB4. Next, we analyzed changes in Fe content in WT, *myb4-1*, *MYB4-COM*, and *MYB4-OE* plants under normal and Fe deficiency stress. Under normal conditions, the Fe content in WT roots and shoots was not significantly different from that in roots and shoots of *myb4-1*, *MYB4-COM*, and *MYB4-OE* plants (Fig. 2I–L); under Fe-deficiency stress, the Fe content in roots of *myb4-1* was higher compared with that of the Ler plants (Fig. 2J), that in roots of *MYB4-COM* plants was not significantly different from that of the Ler plants (Fig. 2J), and that in *MYB4-OE* plants had lower Fe content in roots compared to the Col plants (Fig. 2L). Notably, shoot Fe levels remained unaffected across all genotypes during Fe deprivation (Fig. 2I,K), demonstrating MYB4 specifically modulates root Fe homeostasis during stress adaptation through regulating FIT-dependent expression of *IRT1* and *FRO2*.

To further evaluate the specificity of *MYB4* function in micronutrient deficiency responses, we investigated the growth of Ler and *myb4-1* mutant plants under conditions of manganese (Mn), copper (Cu) and zinc (Zn) deficiency. Unlike the enhanced tolerance observed under Fe deficiency stress, *myb4-1* mutants showed no obvious phenotypic differences compared with Ler plants under Mn-, Cu-, and Zn-deficient conditions, suggesting that *MYB4* primarily functions in the regulation of Fe-deficiency responses (Appendix Fig. S1).

### Fe-deficiency stress-induced MYB4 degradation releases MYB4-mediated inhibition of FIT and bHLH38/39 heterodimer formation

**Figure 3:**
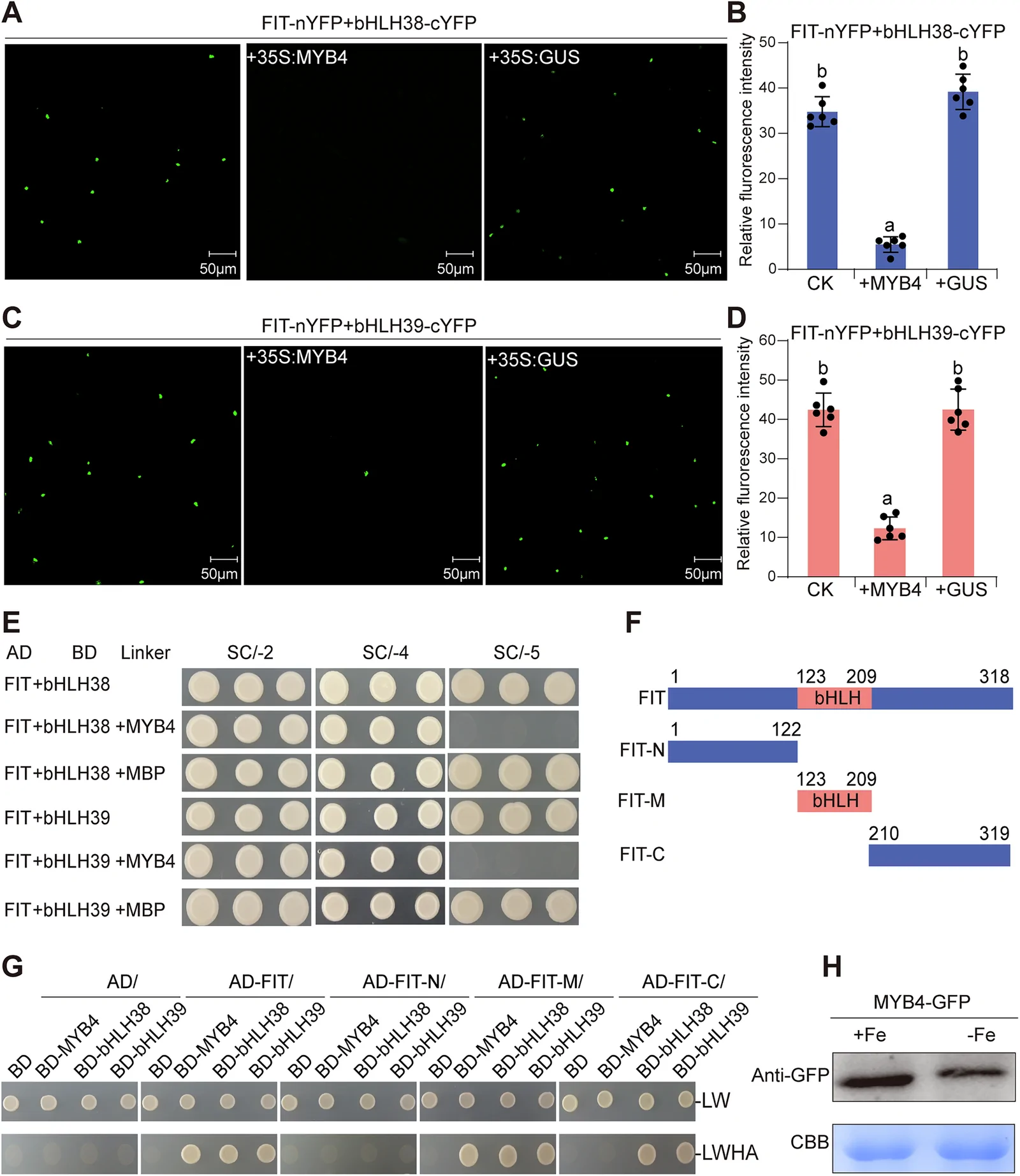
MYB4 interferes with the formation of FIT and bHLH38/39 heterodimers. (**A**,** C**) BiFC analysis showed that MYB4 attenuated the interaction of FIT with bHLH38 or bHLH39. The YFP fluorescence in each combination was detected after 48 h of coexpression. GUS was used as a negative control; FIT/bHLH38 and FIT/bHLH39 interaction were used as the positive control (CK). Scale bar: 50 μm. (**B**,** D**) Quantitative analysis of YFP fluorescence intensity. Values shown are means ± SD (*n* = 6 biological replicates). Significance was determined using Ordinary one-way ANOVA followed by Tukey’s test. (**B**) *P* values <0.0001 (FIT-nYFP+bHLH38-cYFP vs 35S::MYB4), 0.1351 (FIT-nYFP+bHLH38-cYFP vs FIT-nYFP+bHLH38-cYFP+35S:GUS). (**D**) *P* values <0.0001(FIT-nYFP+bHLH39-cYFP vs FIT-nYFP+bHLH39-cYFP+35S:MYB4), 0.9991(FIT-nYFP+bHLH39-cYFP vs FIT-nYFP+bHLH39-cYFP+35S:GUS). (**E**) The yeast three-hybrid (Y3H) assay showed that MYB4 competes with either bHLH38 or bHLH39 for interaction with FIT. MBP fused to bHLH38 or MBP fused to bHLH39 was used alone as a negative control. AD activation domain, BD binding domain. (**F**) Schematic representation of the full-length and truncated form of the FIT protein. (**G**) Yeast two-hybrid analysis indicates that the M-terminal region of FIT interacts with MYB4, bHLH38, and bHLH39. (**H**) MYB4 protein accumulation under Fe deficiency stress. Source data are available online for this figure.

Previous studies have shown that FIT must interact with bHLH38/39/100/101 to regulate the transcription of *FRO2* and *IRT1* under Fe deficiency stress (Wild et al, 2016), and we found that FIT can interact with MYB4. To unravel the mechanism of MYB4 regulation of FIT-dependent Fe deficiency response, we further investigated whether MYB4 affects the formation of heterodimers of FIT with bHLH38/39 by using the BiFC assay. A strong YFP fluorescence signal could be observed when FIT-nYFP was expressed together with bHLH38/39-cYFP in *N. benthamiana* leaves (Fig. 3A–D); however, YFP fluorescence signals were significantly weaker when *35S:MYB4* and FIT-nYFP were co-expressed with bHLH38/39-cYFP in *N. benthamiana* leaves (Fig. 3A–D). A yeast three-hybrid (Y3H) assay was also used to analyze the effect of MYB4 on the interactions between FIT and bHLH38/39. Protein–protein interactions could be observed when FIT was co-expressed with bHLH38/39 in SD/4 and SD/5 yeast culture plates (Fig. 3E); however, protein–protein interactions were eliminated when MYB4, FIT and bHLH38/39 were co-expressed in SD/4 and SD/5 yeast culture plates (Fig. 3E). To identify the specific domains of FIT involved in its interaction with MYB4, bHLH38, and bHLH39, we constructed truncated FIT vectors based on the conserved bHLH domain of FIT: the N-terminal fragment (F-N), the M-terminal fragment (F-M), and the C-terminal fragment (F-C), as illustrated in Fig. 3F. Yeast two-hybrid assays revealed that MYB4 specifically interacts with FIT-M, whereas bHLH38 and bHLH39 specifically interact with both FIT-M and FIT-C, but not with FIT-N (Fig. 3G). These results indicate that the M-terminal region of FIT is crucial for mediating the interactions between MYB4, bHLH38, and bHLH39 and FIT. They also confirm that MYB4 inhibits the interaction between FIT and bHLH38/bHLH39 by competitively binding to the M-terminal region of FIT. We further tested whether MYB4 could also interact with bHLH38/39/100/101, and the results showed that MYB4 could not interact with bHLH38/39/100/101 (Appendix Fig. S2B). Due to the fact that MYB7 and MYB32 are homologs of MYB4, we also analyzed whether there is an interaction between FIT and MYB7/32 using the Y2H assay and found that there is no interaction between FIT and MYB7/32 (Appendix Fig. S2A). These results suggest that MYB4 can specifically influence the formation of heterodimers of FIT with bHLH38/39.

To investigate the post-translational regulation of MYB4 under Fe-limited conditions, we monitored changes in MYB4 protein levels in *35S:MYB4-GFP* transgenic plants under normal and Fe-deficient conditions. Immunoblot analysis showed that protein accumulation of MYB4 was significantly reduced under Fe-deficient conditions compared to normal conditions (Fig. 3H). To elucidate the degradation pathway, pharmacological inhibition assays were performed, and the results showed that MG132 could inhibit the degradation of MYB4 (Appendix Fig. S2C). To determine whether MYB4 is degraded by ubiquitination via the ubiquitin-proteasome system (UPS), we performed Co-IP experiments. A dense smear band of the polyubiquitinated form of MYB4 was detected when Ub-HA was transiently co-expressed with MYB4-FLAG (Appendix Fig. S2D), suggesting that MYB4 can be degraded via the 26S/ubiquitin proteasomal pathway under Fe-deficiency stress. Collectively, these findings suggest that Fe-deficiency stress may induce the degradation of the MYB4 protein via the ubiquitin-proteasome pathway, thereby attenuating its inhibitory effect on FIT and bHLH38/39 heterodimer formation.

### MYB4 acts upstream of FIT to regulate plant response to Fe deficiency stress by repressing the transcriptional activity of FIT and bHLH38/39 complexes

**Figure 4:**
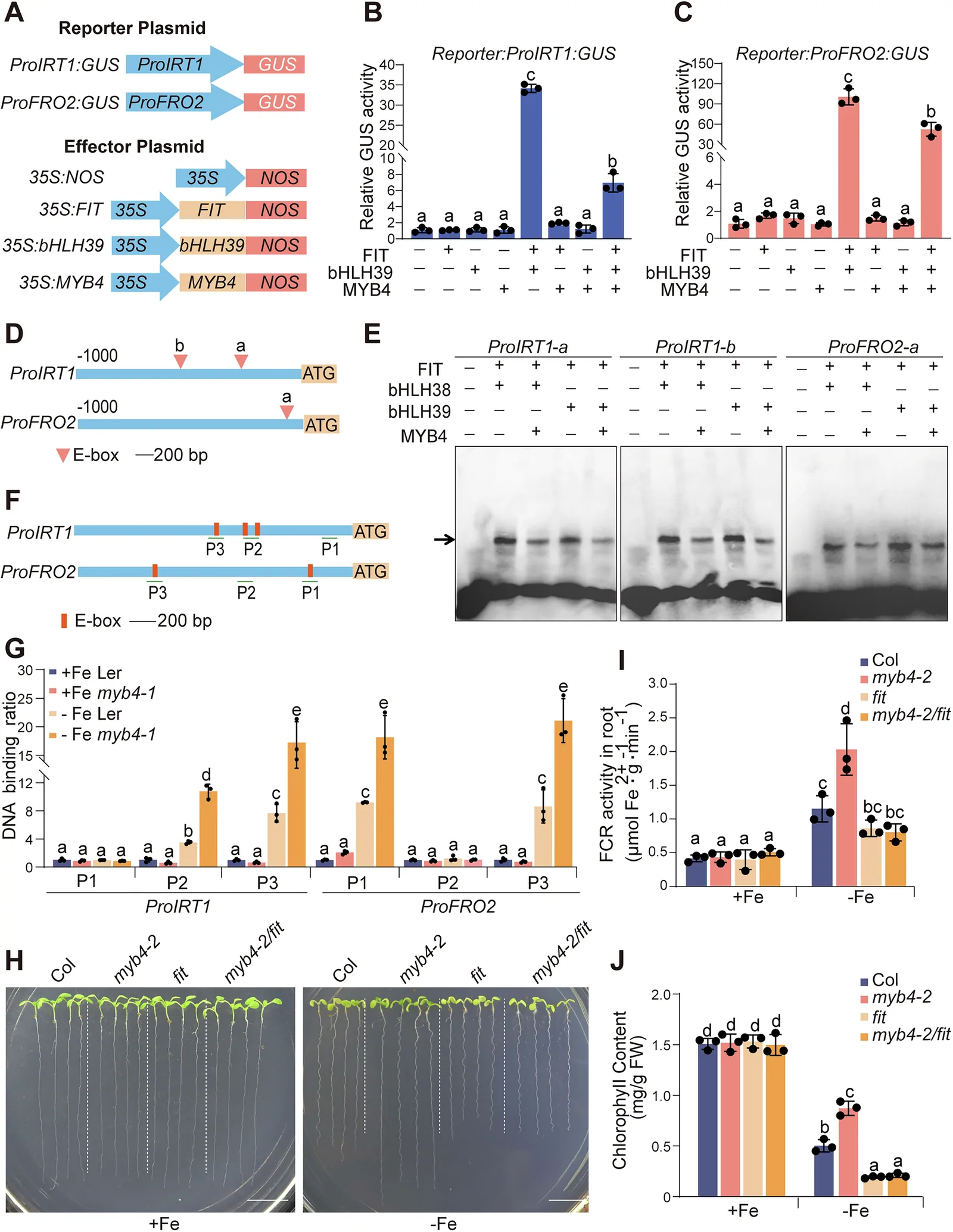
MYB4 suppresses the DNA-binding capability of FIT-bHLH38 and FIT-bHLH39 heterodimeric complexes to the promoters of their downstream targets *IRT1* and *FRO2.* (**A**) Schematic representation of *IRT1* and *FRO2* promoter reporter constructs, effector plasmids and transfection control plasmids. (**B**,** C**) Relative GUS activity of the *ProIRT1:GUS* and *ProFRO2:GUS* reporter genes after expression of FIT or bHLH39 or MYB4 alone or in combination in *N. benthamiana* leaves. 35S-empty vector was used as effector plasmid control. Values shown are means ± SD (*n* = 3 biological replicates). Significance was determined using Ordinary one-way ANOVA followed by Tukey’s test. (**B**) *P* values >0.9999 (*ProIRT1*:GUS vs 35S:FIT+*ProIRT1*:GUS), >0.9999 (ProIRT1:GUS vs 35S:bHLH39+*ProIRT1:GUS*), >0.9999 (*ProIRT1:GUS* vs 35S:MYB4+*ProIRT1*:GUS), <0.0001 (*ProIRT1*:GUS vs 35S:FIT + 35S:bHLH39+*ProIRT1*:GUS), 0.3703 (*ProIRT1*:GUS vs 35S:FIT + 35S:MYB4+*ProIRT1*:GUS), >0.9999 (*ProIRT1*:GUS vs 35S:MYB4 + 35S:bHLH39+*ProIRT1*:GUS), <0.0001 (*ProIRT1*:GUS vs 35S:FIT + 35S:bHLH39 + 35S:MYB4+*ProIRT1*:GUS), <0.0001 (35S:FIT + 35S:bHLH39+*ProIRT1*:GUS vs 35S:FIT + 35S:MYB4+*ProIRT1*:GUS), <0.0001 (35S:FIT + 35S:bHLH39+*ProIRT1*:GUS vs 35S:MYB4 + 35S:bHLH39+*ProIRT1*:GUS), <0.0001 (35S:FIT + 35S:bHLH39+*ProIRT1*:GUS vs 35S:FIT + 35S:bHLH39 + 35S:MYB4+*ProIRT1*:GUS), 0.7202 (35S:FIT + 35S:MYB4+*ProIRT1*:GUS vs 35S:MYB4 + 35S:bHLH39+*ProIRT1*:GUS), <0.0001 (35S:FIT + 35S:MYB4+*ProIRT1*:GUS vs 35S:FIT + 35S:bHLH39 + 35S:MYB4+*ProIRT1*:GUS), <0.0001 (35S:MYB4 + 35S:bHLH39+*ProIRT1*:GUS vs 35S:FIT + 35S:bHLH39 + 35S:MYB4+*ProIRT1*:GUS). (**C**) *P* values >0.9999 (*ProFRO2*:GUS vs 35S:FIT+*ProFRO2*:GUS), >0.9999 (*ProFRO2*:GUS vs 35S:bHLH39+ProFRO2:GUS), >0.9999 (*ProFRO2*:GUS vs 35S:MYB4+*ProFRO2*:GUS), <0.0001 (*ProFRO2*:GUS vs 35S:FIT + 35S:bHLH39+*ProFRO2*:GUS), >0.9999 (*ProFRO2*:GUS vs 35S:FIT + 35S:MYB4+*ProFRO2*:GUS), >0.9999 (*ProFRO2*:GUS vs 35S:MYB4 + 35S:bHLH39+*ProFRO2*:GUS), <0.0001 (*ProFRO2*:GUS vs 35S:FIT + 35S:bHLH39 + 35S:MYB4+*ProFRO2*:GUS), <0.0001 (35S:FIT + 35S:bHLH39+*ProFRO2*:GUS vs 35S:FIT + 35S:MYB4+*ProFRO2*:GUS), <0.0001 (35S:FIT + 35S:bHLH39+*ProFRO2*:GUS vs 35S:MYB4 + 35S:bHLH39+*ProFRO2*:GUS), <0.0001 (35S:FIT + 35S:bHLH39+*ProFRO2*:GUS vs 35S:FIT + 35S:bHLH39 + 35S:MYB4+*ProFRO2*:GUS), >0.9999 (35S:FIT + 35S:MYB4+*ProFRO2*:GUS vs 35S:MYB4 + 35S:bHLH39+*ProFRO2*:GUS), <0.0001 (35S:FIT + 35S:MYB4+*ProFRO2*:GUS vs 35S:FIT + 35S:bHLH39 + 35S:MYB4+*ProFRO2*:GUS), <0.0001 (35S:MYB4 + 35S:bHLH39+*ProFRO2*:GUS vs 35S:FIT + 35S:bHLH39 + 35S:MYB4+*ProFRO2*:GUS). (**D**) Schematic representation of FIT forming a heterodimer with bHLH38/39 to bind to the *IRT1* and *FRO2* promoter sites of the target genes (a and b). (**E**) EMSA assay to analyse the effect of MYB4 on the ability of FIT and bHLH38/39 to bind to the *IRT1* and *FRO2* promoter regions. Black arrows indicate binding probes. (**F**) Schematic representation of the E-box (red line) binding sites on the *IRT1* and *FRO2* promoters. The red line represents the position of the E-box (CANNTG). The green line denotes the DNA region amplified by ChIP-qPCR. (**G**) ChIP-qPCR was used to detect the effect of MYB4 on the ability of FIT to bind to the *IRT1* promoter regions. The promoters of *ProIRT1-P1* was served as negative controls. Values shown are means ± SD (*n* = 3 biological replicates). Significance was determined using Ordinary one-way ANOVA followed by Tukey’s test. *ACTIN2* was used as an internal control. *P* values = 0.5682 (+Fe P1-Ler vs +Fe P1-*myb4-1*), 0.9939 (+Fe P1-Ler vs -Fe P1-Ler), 0.3887 (+Fe P1-Ler vs -Fe P1-*myb4-1*), 0.7088 (+Fe P1-*myb4-1* vs -Fe P1-Ler), 0.9839 (+Fe P1-*myb4-1* vs -Fe P1-*myb4-1*), 0.514 (-Fe P1-Ler vs-Fe P1-*myb4-1*); 0.8416 (+Fe P2-Ler vs +Fe P2-*myb4-1*), 0.0008 (+Fe P2-Ler vs -Fe P2-Ler), <0.0001 (+Fe P2-Ler vs -Fe P2-*myb4-1*), 0.0003 (+Fe P2-*myb4-1* vs -Fe P2-Ler), <0.0001 (+Fe P2-*myb4-1* vs -Fe P2-*myb4-1*), <0.0001 (-Fe P2-Ler vs-Fe P2-*myb4-1*); *P* values 0.9952 (+Fe P3-Ler vs +Fe P3-*myb4-1*), 0.0129 (+Fe P3-Ler vs -Fe P3-Ler), <0.0001 (+Fe P3-Ler vs -Fe P3-*myb4-1*), 0.0095 (+Fe P3-*myb4-1* vs -Fe P3-Ler), <0.0001 (+Fe P3-*myb4-1* vs -Fe P3-*myb4-1*), 0.0014 (-Fe P3-Ler vs-Fe P3-*myb4-1*) (The following is the same order). ChIP-qPCR was used to detect the effect of MYB4 on the ability of FIT to bind to the *FRO2* promoter regions. The promoters of *ProFRO2-P2* served as negative controls. *P* values = 0.998, 0.0033, <0.0001, 0.0041, <0.0001, 0.0019; 0.8516, 0.6036, >0.9999 0.2398, 0.8299, 0.6298; 0.9986, 0.0142, <0.0001, 0.0116, <0.0001, 0.0015. (**H**) Tolerance analysis of Col, *myb4-2*, *fit* and *myb4-2/fit* double mutant plants to Fe deficiency stress. Bar = 1 cm. (**I**, **J**) Root FCR activity I and total chlorophyll content J of Col, *myb4-2*, *fit*, and *myb4-2/fit* double mutant plants under normal and Fe-deficient stress conditions. (**I**) Values shown are means ± SD (*n* = 3 biological replicates). Significance was determined using two-way ANOVA followed by Tukey’s test. *P* values >0.9999 (+Fe Col vs +Fe *myb4-2*), >0.9999 (+Fe Col vs +Fe *fit*), 0.9995 (+Fe Col vs +Fe *myb4-2/fit*), >0.9999 (+Fe *myb4-2* vs +Fe *fit*), 0.9999 (+Fe *myb4-2* vs +Fe *myb4-2/fit*), 0.9978 (+Fe *fit* vs +Fe *myb4-2/fit*), 0.0003 (-Fe Col vs -Fe *myb4-2*), 0.3127 (-Fe Col vs -Fe *fit*), 0.2664 (-Fe Col vs -Fe *myb4-2/fit*), <0.0001 (-Fe *myb4-2* vs -Fe *fit*), <0.0001 (-Fe *myb4-2* vs -Fe *myb4-2/fit*), >0.9999(-Fe *fit* vs -Fe *myb4-2/fit*), (The following is the same order). (**J**) *P* values >0.9999, 0.9965, >0.9999, 0.9998, >0.9999, 0.991, <0.0001, 0.0008, 0.0016, <0.0001, <0.0001, >0.9999. Source data are available online for this figure.

Having found that MYB4 proteins can influence the formation of heterodimers of FIT with bHLH38/39, we further investigated the effect of these protein interactions on the transcriptional activity of FIT and bHLH38/39 for the target genes *IRT1* and *FRO2*. To this end, we carried out transient transcriptional activation assays in *N. benthamiana* leaves. There was no effect on *ProFRO2:GUS* or *ProIRT1:GUS* activity when MYB4, FIT, or bHLH39 were expressed alone in *N. benthamiana* leaves (Fig. 4A–C). Nevertheless, simultaneous expression of FIT and bHLH39 resulted in a significant increase in *ProIRT1:GUS* or *ProFRO2:GUS* reporter activity (Fig. 4B,C). Conversely, simultaneous expression of MYB4, FIT, and bHLH39 in *N. benthamiana* leaves resulted in a significant reduction in the reporter activity of *ProIRT1:GUS* or *ProFRO2:GUS* (Fig. 4B,C). This result suggests that MYB4 represses the transcriptional activity of FIT/bHLH39 on the downstream target genes *IRT1/FRO2* in *N. benthamiana* leaves.

Previous studies reported that FIT forms a heterodimer with bHLH38/39 capable of binding to the E-box (CANNTG) region on the *IRT1* or *FRO2* promoters (Fig. 4D) (Wild et al, 2016). Next, we employed electrophoretic mobility shift assay (EMSA) with recombinant proteins to examine if MYB4 alters the binding of FIT/bHLH38 and FIT/bHLH39 heterodimers to the target promoters of *IRT1* and *FRO2*. EMSA assays confirmed that the FIT/bHLH38 heterodimer binds to each of the selected motifs, and that the presence of MYB4 diminishes the binding capacity of both FIT/bHLH38 and FIT/bHLH39 heterodimers to specific E-box motifs (Fig. 4E). We further performed ChIP-qPCR assays on Ler and *myb4-1* plants grown under normal and Fe-deficient conditions (Fig. 4F,G). There was no significant difference in the binding of FIT with the *IRT1* and *FRO2* promoters between Ler and *myb4-1* plants under normal conditions, but under Fe-deficient conditions, the binding of FIT with the *IRT1* and *FRO2* promoters was significantly enhanced in *myb4-1* plants compared with Ler plants (Fig. 4F,G). In addition, we also investigated whether MYB4 can directly bind to the promoters of *IRT1* and *FRO2*. The results of yeast one-hybrid experiments revealed that MYB4 could not bind directly to the *IRT1* and *FRO2* promoters (Appendix Fig. S2E). Collectively, these results suggest that MYB4 can inhibit the binding of FIT and bHLH38/39 heterodimers to the *IRT1* and *FRO2* promoters, thereby negatively regulating their expression.

To examine the genetic relationship between FIT and MYB4, we crossed *myb4-2* knockout mutant plants (Appendix Fig. S3A) with *fit* (Appendix Fig. S3B) knockdown mutant plants to obtain the *myb4-2/fit* double mutant. Previous studies have shown that *fit* mutants exhibit stunted growth and serious Fe deficiency signs (Colangelo and Guerinot, 2004). Likewise, we also found that the *fit* mutant plants showed a sensitive phenotype under Fe deficiency stress (Fig. 4H). At the same time, the *myb4-2/fit* double mutant exhibited the same Fe-deficiency-sensitive phenotype as the *fit* mutant plants, which can be demonstrated by their similar FCR activity (Fig. 4I) and total chlorophyll content (Fig. 4J). These results suggest that MYB4 functions upstream of FIT to regulate plant response to Fe deficiency stress.

To investigate whether *MYB4* broadly affects additional previously reported target genes under Fe-deficient conditions, we examined the expression of *ADT6* and *MAN3*, two established *MYB4*-regulated genes involved in flavonoid biosynthesis and cadmium stress response, respectively (Wang et al, 2020; Yan et al, 2021). Consistent with previous reports, *ADT6* transcript levels were significantly elevated in th*e myb4-2* mutant under both Fe-sufficient and Fe-deficient conditions (Appendix Fig. S3G), whereas *MAN3* expression was reduced relative to Col plants (Appendix Fig. S3H). However, neither gene displayed expression patterns that paralleled the enhanced Fe-deficiency tolerance phenotype of the *myb4-2* mutant. These observations indicate that MYB4 retains its previously characterized regulatory activities under Fe-deficient conditions but suggest that the enhanced Fe uptake phenotype of *myb4* mutants cannot be readily explained by alterations in these established MYB4 target pathways. Taken together with the genetic and biochemical evidence provided above, these findings further support the conclusion that MYB4 regulates the Fe-deficiency response primarily by modulating FIT-dependent pathways.

### MPK3 interacts with MYB4

**Figure 5:**
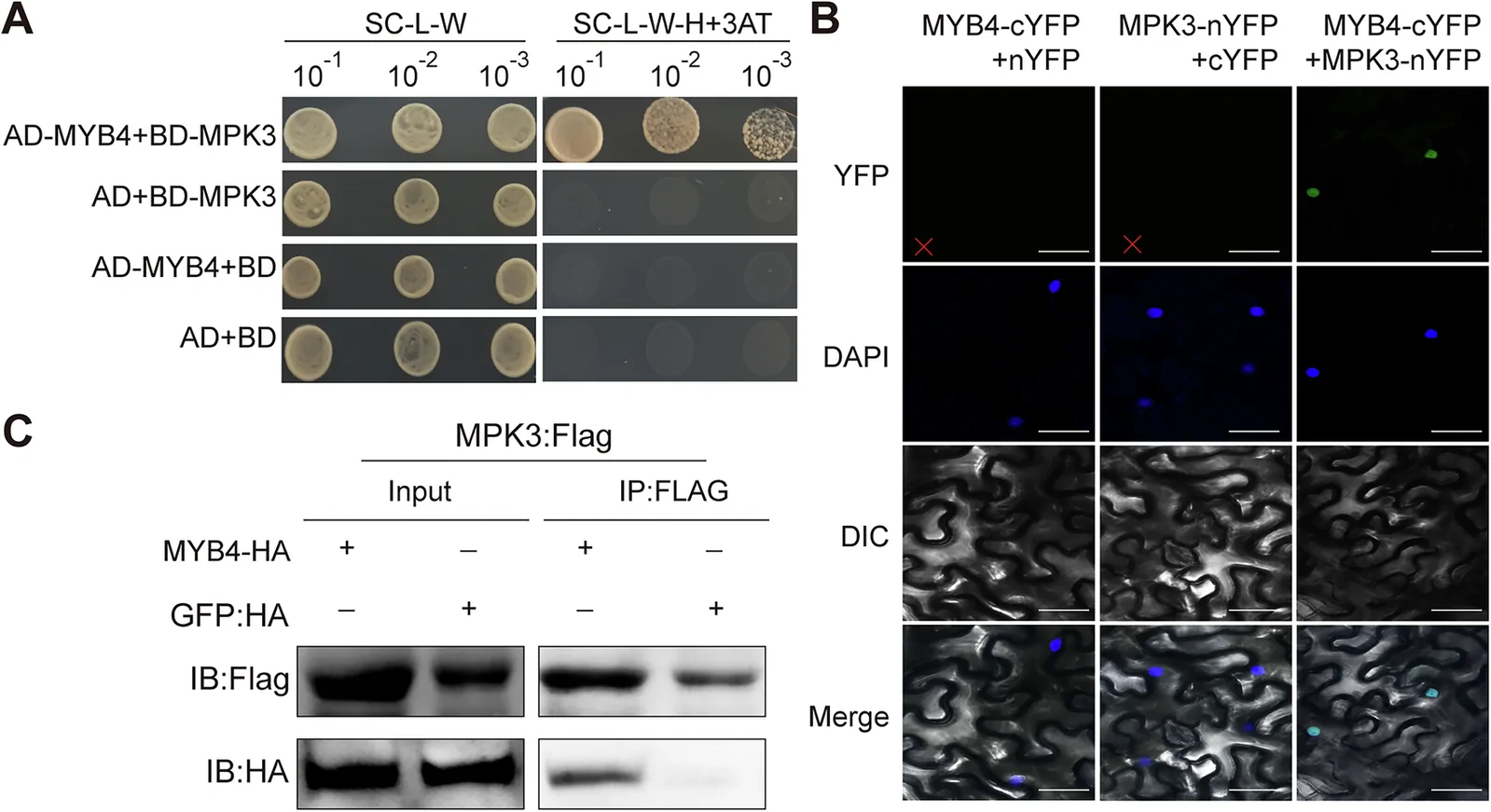
Analysis of the interactions between MPK3 and MYB4 proteins. (**A**) Y2H assay to detect interactions between MPK3 and MYB4 proteins. (**B**) The BiFC assay confirms the interaction between MPK3 and MYB4 proteins. Scale bar: 50 μm. The red marking indicates that no fluorescence signal was detected. (**C**) The Co-IP assay analyses the interaction between MPK3 and MYB4 in plant cells. Source data are available online for this figure.

To elucidate the molecular mechanism underlying MYB4-mediated Fe deficiency responses in plants, we further performed a Y2H screening with MYB4 as bait, which identified potential interactions with MPK3 kinase. Y2H assay confirmed direct physical interactions between MYB4 and both MPK3 (Fig. 5A). BiFC and Co-IP assays were performed to further validate these interactions (Fig. 5B,C). These results confirm that MYB4 interacts with MPK3 both in vivo and in vitro.

### MPK3 functions upstream of MYB4 to mediate plant responses to Fe deficiency stress through the FIT-dependent pathway

**Figure 6:**
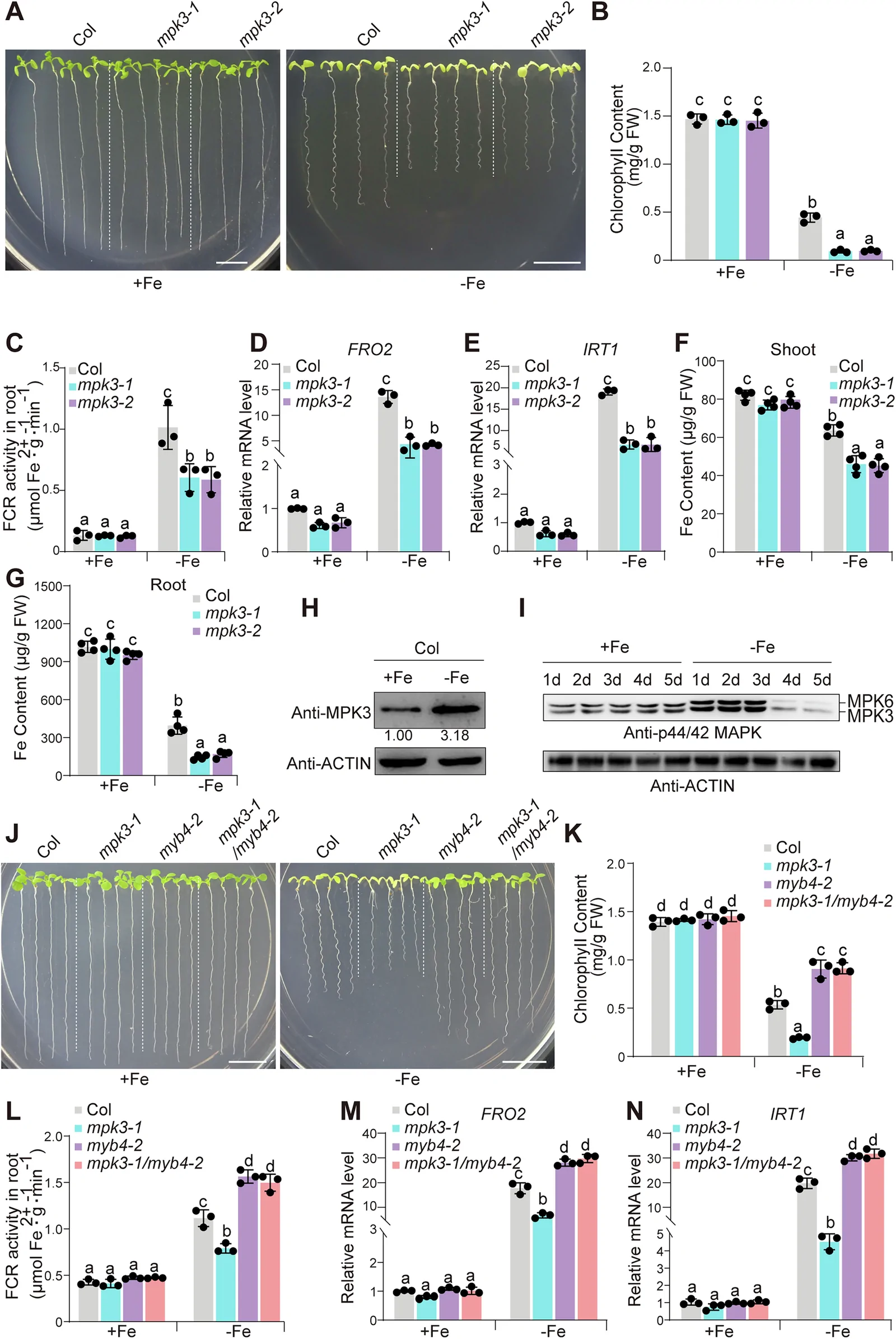
MPK3 mediates plant responses to Fe deficiency stress via FIT-dependent signaling pathways. (**A**) Phenotypic analysis of Col, *mpk3-1* and *mpk3-2* mutant plants under Fe deficiency stress. Scale bar: 1 cm. (**B**,** C**) The total chlorophyll content and root FCR activity of Col, *mpk3-1* and *mpk3-2* mutant plants under both normal and Fe deficiency stress conditions. (**B**) Values shown are means ± SD (*n* = 3 biological replicates). Significance was determined using two-way ANOVA followed by Tukey’s test. *P* values >0.9999 (+Fe Col vs +Fe *mpk3-1*), 0.9978 (+Fe Col vs +Fe *mpk3-2*), 0.9998 (+Fe *mpk3-1* vs +Fe *mpk3-2*), <0.0001 (-Fe Col vs -Fe *mpk3-1*), <0.0001(-Fe Col vs -Fe *mpk3-2*), >0.9999 (-Fe *mpk3-1* vs -Fe *mpk3-2*) (The following is the same order). (**C**) *P* values >0.9999, >0.9999, >0.9999, 0.0028, 0.0021, >0.9999. (**D**,** E**) RT-qPCR analysis of *IRT1* and *FRO2*, key genes for Fe uptake in roots of Col and *mpk3* mutant plants under normal and Fe-deficient stress. *ACTIN8* was used as an internal control. Values shown are means ± SD (*n* = 3 biological replicates). Significance was determined using two-way ANOVA followed by Tukey’s test. (**D**) *P* values = 0.988, 0.9947, >0.9999, <0.0001, <0.0001, 0.9997. (**E**) *P* values = 0.995, 0.9932, >0.9999, <0.0001, <0.0001, >0.9999. (**F**,** G**) Fe content of the roots and shoots of Col and *mpk3* mutant plants under both normal and Fe-deficiency conditions. Values shown are means ± SD (*n* = 4 biological replicates). Significance was determined using two-way ANOVA followed by Tukey’s test. (**F**) *P* values = 0.5012, 0.5106, >0.9999, <0.0001, <0.0001, 0.9994. (**G**) *P* values = 0.9949, 0.4594, 0.7603, <0.0001, <0.0001, 0.979. (**H**) Western blotting to analyze changes in MPK3 protein levels in Col plants under normal and Fe deficiency stress. Proteins were extracted from treated seedlings and analyzed by Western blotting using anti-MPK3 antibodies (upper panel). Actin was used as a control. (**I**) MAPK activities in Col plants under Fe deficiency stress were measured by the immunoprecipitation kinase assay. Col seedlings were harvested for protein extraction, followed by the detection of MAPK kinase activity using anti-p44/42 MAPK antibody. Actin was used as a control. (**J**) Tolerance analysis of Col, *mpk3-1*, *myb4-2* and *mpk3-1/myb4-2* double mutant plants to Fe deficiency stress. Scale bar: 1 cm. (**K**,** L**) Total chlorophyll content (**K**) and root FCR activity (**L**) of Col, *mpk3-1*, *myb4-2* and *mpk3-1****/****myb4-2* double mutant plants under normal and Fe-deficient stress conditions. Values shown are means ± SD (*n* = 3 biological replicates). Significance was determined using two-way ANOVA followed by Tukey’s test. (**K**) *P* values = 0.9993 (+Fe Col vs +Fe *mpk3-1*), 0.9935 (+Fe Col vs +Fe *myb4-2*), 0.8645 (+Fe Col vs +Fe *mpk3-1/ myb4-2*), >0.9999 (+Fe *mpk3-1* vs +Fe *myb4-2*), 0.9886 (+Fe *mpk3-1* vs +Fe *mpk3-1/ myb4-2*), 0.9983 (+Fe *myb4-2* vs +Fe *mpk3-1/ myb4-2*), <0.0001 (-Fe Col vs -Fe *mpk3-1*), <0.0001 (-Fe Col vs -Fe *myb4-2*), <0.0001 (-Fe Col vs -Fe *mpk3-1/ myb4-2*), <0.0001 (-Fe *mpk3-1* vs -Fe c*myb4-2*), <0.0001 (-Fe *mpk3-1* vs *-*Fe *mpk3-1/ myb4-2*), >0.9999(-Fe *myb4-2* vs -Fe *mpk3-1/ myb4-2*), (The following is the same order). (**L**) *P* values >0.9999, 0.9676, 0.9664, 0.8894, 0.8868, >0.9999, <0.0001, <0.0001, <0.0001, <0.0001, <0.0001, 0.8761. (**M**,** N**) RT-qPCR analysis of *FRO2* and *IRT1*, key genes for Fe uptake in roots of Col, *mpk3-1*, *myb4-2* and *mpk3-1/myb4-2* double mutant plants under normal and Fe-deficient stress. *ACTIN8* was used as an internal control. Values shown are means ± SD (*n* = 3 biological replicates). Significance was determined using two-way ANOVA followed by Tukey’s test. (**M**) *P* values > 0.9999, >0.9999, **>**0.9999, >0.9999, >0.9999, >0.9999, <0.0001, <0.0001, <0.0001, <0.0001, <0.0001, 0.6462. (**N**) *P* values >0.9999, >0.9999, >0.9999, >0.9999, >0.9999, >0.9999, <0.0001, <0.0001, <0.0001, <0.0001, <0.0001, 0.611. Source data are available online for this figure.

To further investigate whether MPK3 plays a role in the response to Fe deficiency stress, we performed Fe deficiency stress assays using two Arabidopsis gene loss-of-function mutants: *mpk3-1* and *mpk3-2*. Under normal conditions, the growth phenotype of the *mpk3* mutant is comparable to that of Col plants. However, under Fe deficiency stress, the *mpk3* mutant plants exhibited significantly reduced tolerance compared to Col plants (Fig. 6A). Furthermore, their total chlorophyll content and FCR activity were markedly lower than those observed in Col plants (Fig. 6B,C). We next investigated whether MPK3 mediates Fe-deficiency stress in plants through the FIT-dependent pathway by analyzing the expression of Fe uptake-related genes *IRT1* and *FRO2* in *mpk3* mutants. The results demonstrated that under Fe deficiency stress, the transcription levels of *IRT1* and *FRO2* were significantly reduced in *mpk3* mutant plants compared with Col plants (Fig. 6D,E). Furthermore, we analyzed the Fe content in *mpk3* mutants. Under Fe-sufficient conditions, no significant differences in Fe content were detected between the roots and shoots of Col plants and those of *mpk3* mutant plants (Fig. 6F,G). In contrast, under Fe-deficient stress conditions, the Fe content in both roots and shoots of *mpk3* mutant plants was significantly lower than that in Col plants (Fig. 6F,G).

**Figure EV2:**
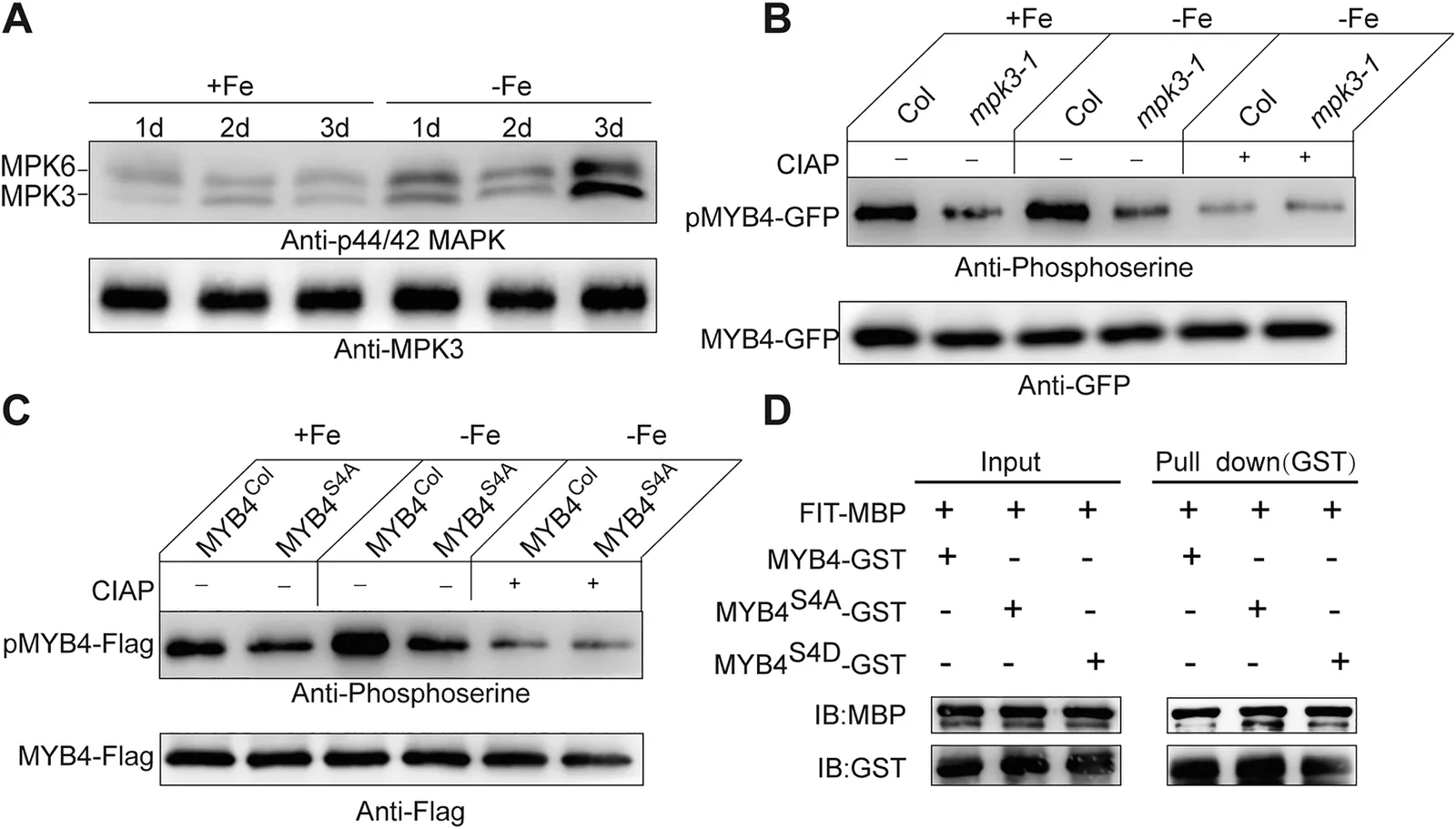
Under Fe-deficiency stress, MPK3 regulates MYB4 accumulation by phosphorylating MYB4 at Ser4. (**A**) Fe deficiency enhances MPK3 kinase activity. Proteins were extracted from wild-type Arabidopsis seedlings grown under Fe-sufficient or Fe-deficient conditions and subjected to gel-based kinase assays. MPK3 kinase activity was elevated under Fe-deficient conditions. MAPK activation was verified by immunoblotting with an anti-phospho-p44/42 MAPK antibody. Equivalent amounts of MPK3 protein were used for kinase activity measurements to evaluate changes in intrinsic kinase activity. (**B**) The effect of *mpk3-1* mutation on the phosphorylation of MYB4. The seedlings of the Col plants carrying the *35S:MYB4-GFP* gene and the *mpk3-1* plants were all cultivated on a medium with sufficient Fe for 10 days. Then, they were transferred to either Fe-sufficient or Fe-deficient medium for 3 days and immediately harvested. The seedlings were collected and processed for immunoprecipitation of MYB4-GFP, and subsequently, phosphorylation detection was performed using anti-Phosphoserine. (**C**) Effect of Ser4A mutation on MYB4 phosphorylation. The seedlings in the *myb4-2* background that carried the *ProMYB4:MYB4-Flag* (MYB4^Col^) or *ProMYB4:MYB4*^S4A^*-Flag* (MYB4^S4A^) genes were all cultivated on a medium containing sufficient Fe for 10 days. Then, they were transferred to either Fe-sufficient or Fe-deficient medium for 3 days and immediately harvested. The seedlings were collected and processed for immunoprecipitation of MYB4-Flag, and subsequently, phosphorylation detection was performed using anti-Phosphoserine. (**D**) The effects of the Ser4A and Ser4D mutations on the interaction between MYB4 and FIT were examined using pull-down assays. For the pull-down assays, recombinant FIT-MBP protein was incubated with MYB4-GST, MYB4^S4A^-GST, or MYB4^S4D^-GST proteins at 4 °C for 5 h.

To further elucidate the functional involvement of MPK3 in the plant response to Fe-deficiency stress, we analyzed changes in MPK3 protein levels in wild-type Arabidopsis seedlings under Fe-deficient conditions. The results showed that MPK3 protein accumulation was markedly increased under Fe deficiency stress (Fig. 6H). Consistent with this observation, kinase assays performed using equal amounts of total protein revealed substantially higher MPK3 kinase activity in Fe-deficient seedlings than in Fe-sufficient seedlings (Fig. 6I). To further determine whether this increase was solely attributable to elevated MPK3 abundance, we performed an additional kinase assay using equivalent amounts of MPK3 protein from Fe-sufficient and Fe-deficient seedlings. Under these normalized conditions, MPK3 derived from Fe-deficient seedlings still exhibited markedly higher kinase activity than MPK3 from Fe-sufficient seedlings (Fig. EV2A). These results indicate that Fe deficiency not only promotes MPK3 protein accumulation but also enhances its intrinsic kinase activity. Therefore, the increased MPK3 signaling output under Fe deficiency is attributable to both elevated protein abundance and increased kinase activation. These results indicate that MPK3 plays a positive regulatory role in the response to Fe-deficiency stress in *Arabidopsis* via modulating the expression of FIT-regulated Fe uptake genes.

To explore the genetic relationship between MYB4 and MPK3, we generated *mpk3-1/myb4-2* double mutant plants. We found that the *mpk3-1/myb4-2* double mutant exhibited the same Fe-deficiency-tolerant phenotype as the *myb4-2* mutant plants, which can be demonstrated by their similar total chlorophyll content and FCR activity (Fig. 6J–L). Further, we examined the expression of Fe uptake-related genes *IRT1* and *FRO2* in Col, *myb4-2*, *mpk3-1*, and the *mpk3-1/myb4-2* double mutant under Fe-deficient conditions. The results revealed that, under Fe deficiency stress, the transcript levels of *IRT1* and *FRO2* were significantly reduced in *mpk3-1* mutants relative to Col plants (Fig. 6M,N). Notably, the expression of both genes was markedly upregulated in the *myb4-2* single mutant and the *mpk3-1/myb4-2* double mutant compared to Col plants (Fig. 6M,N). Collectively, these findings indicate that MPK3 acts upstream of MYB4 and mediates plant responses to Fe deficiency through the FIT-dependent pathway.

### MPK3 phosphorylates the Ser4 residue of MYB4 to regulate its stability under Fe-deficient conditions

**Figure 7:**
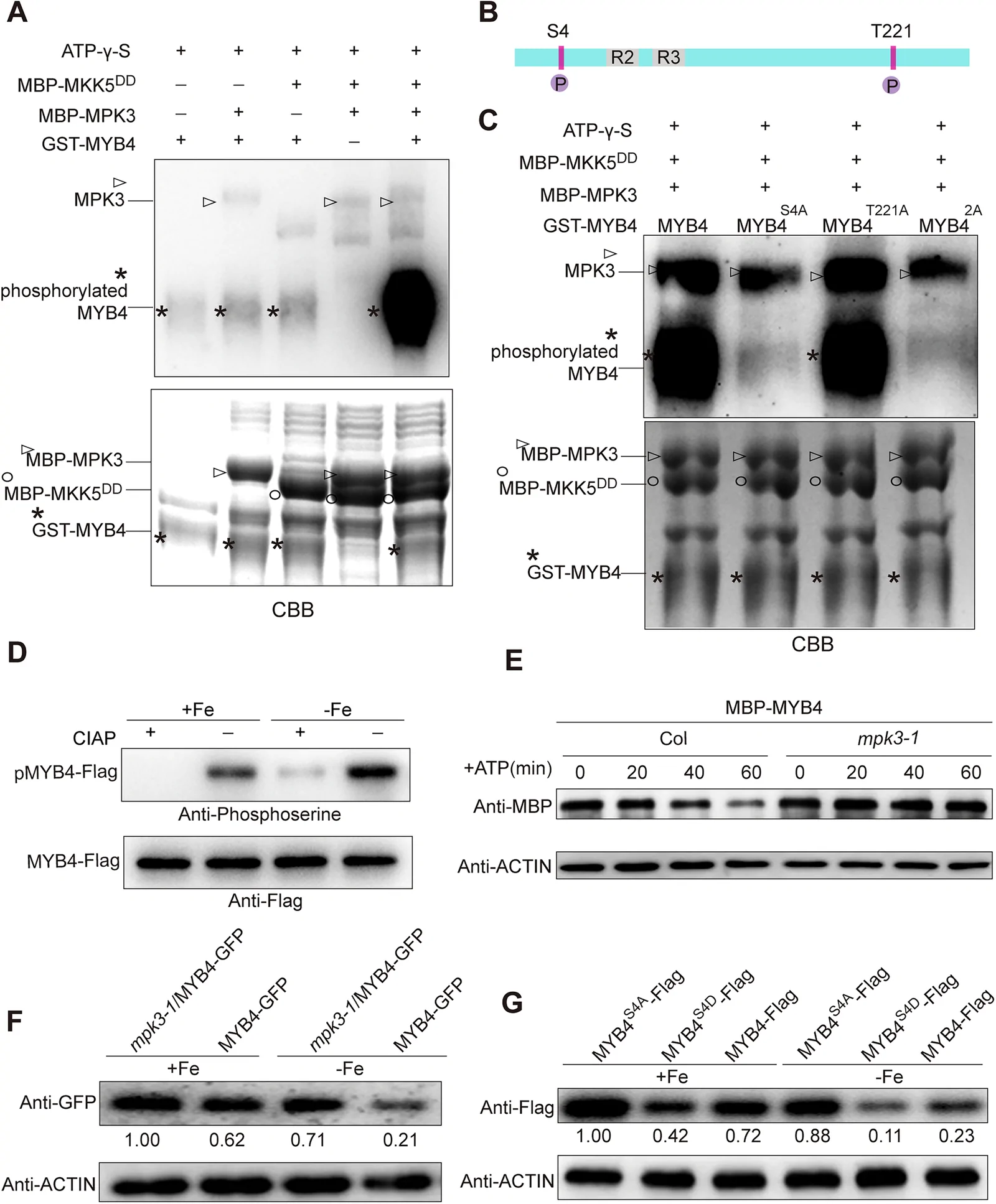
MPK3-mediated phosphorylation at Ser4 attenuates MYB4 stability under Fe-deficiency stress. (**A**) In vitro phosphorylation assays demonstrate that MPK3 phosphorylates MYB4. CBB, Coomassie Brilliant Blue. (**B**) Diagrammatic representation of putative MAPK phosphorylation sites in the MYB4 protein sequence. (**C**) In vitro phosphorylation of wild-type and mutant MYB4 by MPK3. CBB, Coomassie Brilliant Blue. (**D**) In vivo phosphorylation of MYB4 is enhanced by Fe deficiency stress. *ProMYB4:MYB4-Flag/myb4-2* seedlings were germinated on Fe-sufficient medium for 10 days. Then, they were transferred to either Fe-sufficient or Fe-deficient medium for 3 days and immediately harvested. (**E**) In a cell-free degradation assay, MPK3 promoted the degradation of MYB4. MYB4 was detected using an anti-MBP antibody. Actin was utilized as a control. (**F**) Effects of MPK3 on MYB4 stability under normal and Fe-deficient conditions. MYB4 was detected using an anti-GFP antibody. Actin was utilized as a control. (**G**) The stability of MYB4, MYB4^S4A^ and MYB4^S4D^ proteins in vivo. *ProMYB4:MYB4-Flag/myb4-2, ProMYB4:MYB4-Flag*^*S4A*^*/myb4-2* and *ProMYB4:MYB4-Flag*^*S4D*^*/myb4-2* transgenic plants were grown vertically on normal medium for 10 days and then transferred to normal and Fe-deficient medium for 3 days and immediately harvested. Proteins were extracted from treated seedlings and analyzed by Western blotting using anti-Flag antibody (upper panel). Actin was used as a control. Source data are available online for this figure.

Given that MYB4 interacts with MPK3, we performed in vitro phosphorylation assays to determine whether MPK3 directly phosphorylates MYB4. The results revealed that MYB4 is effectively phosphorylated by MPK3 (Fig. 7A). Notably, the amino acid sequence of MYB4 was predicted to have two potential MAPK phosphorylation sites, Ser4 and Thr221 (Fig. 7B). To further confirm the MAPK phosphorylation site of MYB4, two Ser4 and Thr221 residues were single or double-site mutated to Ala (MYB4^S4A^, MYB4^T221A^, and MYB4^2A^). It was found that MYB4^T221A^ could still be phosphorylated by MPK3, whereas MYB4^S4A^ and MYB4^2A^ were no longer phosphorylated by MPK3, suggesting that Ser4 is the primary MAPK phosphorylation site on MYB4 (Fig. 7C). Furthermore, considering that MPK3 functions as a protein kinase in regulating the Fe deficiency response, we investigated whether MYB4 is phosphorylated under Fe-deficient conditions. The in vivo phosphorylation assay revealed that the phosphorylation signal of MYB4-FLAG was significantly enhanced following Fe deficiency treatment, and this enhancement was abolished by calf intestinal alkaline phosphatase (CIAP) (Fig. 7D), demonstrating that MYB4 undergoes phosphorylation in planta under Fe-deficient conditions. To test whether MYB4 phosphorylation depends on MPK3 in planta, we analyzed the phosphorylation status of MYB4 in the *mpk3* mutant background. The results show a clear reduction in the phosphorylation signal of MYB4 in *mpk3* compared with Col plants under Fe-deficient conditions (Fig. EV2B). This trend indicates that MPK3 is a major kinase responsible for MYB4 phosphorylation in vivo. Furthermore, to assess the effect of the Ser4A mutation, we examined the phosphorylation level of *ProMYB4:MYB4*^*S4A*^*-Flag/myb4-2* in transgenic plants. It was found that the phosphorylation signal of MYB4^S4A^ was significantly lower than that of the wild-type MYB4 (Fig. EV2C). This result further confirmed that Ser4 is the main phosphorylation site of MYB4 in plants.

Given that protein phosphorylation is closely associated with protein stability (Ding et al, 2015; Chen et al, 2020) and MYB4 levels decrease under Fe-deficiency conditions (Fig. 3H), we investigated whether MYB4 stability is regulated by MPK3-mediated phosphorylation. We performed a cell-free protein degradation assay to investigate MYB4 protein stability in *mpk3-1* mutant plants. We observed that the prolonged incubation period with ATP progressively enhanced MBP-MYB4 protein degradation in Col plants, whereas MBP-MYB4 protein was degraded at a slower rate in *mpk3-1* plants (Fig. 7E). Furthermore, we generated transgenic lines expressing *35S:GFP-MYB4* in both the *mpk3-1* mutant and Col backgrounds. The Western blotting analysis results showed that under normal conditions, the accumulation level of MYB4 protein in *35S:GFP-MYB4/mpk3-1* plants was significantly higher than that in *35S:GFP-MYB4* plants; under Fe deficiency conditions, the accumulation amount of MYB4 protein in *35S:GFP-MYB4/mpk3-1* plants was still significantly higher than that of the *35S:GFP-MYB4* plants (Fig. 7F). To further investigate the effect of phosphorylation on the protein abundance of MYB4 in plants under Fe deficiency stress, we analyzed the protein levels of MYB4-Flag in the transgenic lines *ProMYB4:MYB4-Flag/myb4-2*, *ProMYB4:MYB4*^*S4A*^*-Flag/myb4-2* (phosphorylation-inactive mutant) and *ProMYB4:MYB4*^*S4D*^*-Flag/myb4-2* (simulated phosphorylation mutant). The results showed that under both normal and Fe-deficient conditions, the dephosphorylation-mimicking *MYB4*^*S4A*^*-Flag* exhibited greater stability than *MYB4-Flag*, whereas the phosphorylation-mimicking *MYB4*^*S4D*^*-Flag* showed the opposite trend (Fig. 7G). Overall, these data demonstrate that MPK3-mediated phosphorylation of MYB4 at Ser4 destabilizes the protein under Fe-deficient conditions.

The preliminary experiments demonstrated that MYB4 is degraded via the 26S ubiquitin-proteasome pathway under Fe-deficient conditions (Appendix Fig. S2C,D). To further investigate the molecular mechanism underlying this degradation, we conducted ubiquitination assays using anti-ubiquitin antibodies to detect the ubiquitination levels of MYB4 and MYB4^S4A^ in *ProMYB4:MYB4-Flag/myb4-2* and *ProMYB4:MYB4*^*S4A*^*-Flag/myb4-2* transgenic plants. We observed that more high-density smear bands corresponding to polyubiquitinated forms of MYB4-Flag were detected in *ProMYB4:MYB4-Flag/myb4-2* transgenic lines after Fe-deficiency stress treatment compared with before treatment (Appendix Fig. S3C). In contrast, the ubiquitination of MYB4^S4A^ was significantly reduced in *ProMYB4:MYB4*^*S4A*^*-Flag/myb4-2* transgenic lines under Fe-deficiency stress compared with *ProMYB4:MYB4-Flag/myb4-2* transgenic plants (Appendix Fig. S3C). These combined results suggest that MPK3-mediated phosphorylation of MYB4 may be associated with enhanced ubiquitination and proteasome-dependent degradation under Fe-deficiency conditions.

### Phosphorylation of MYB4 at Ser4 mediated by MPK3 is essential for the function of MYB4 in response to Fe-deficiency stress

**Figure 8:**
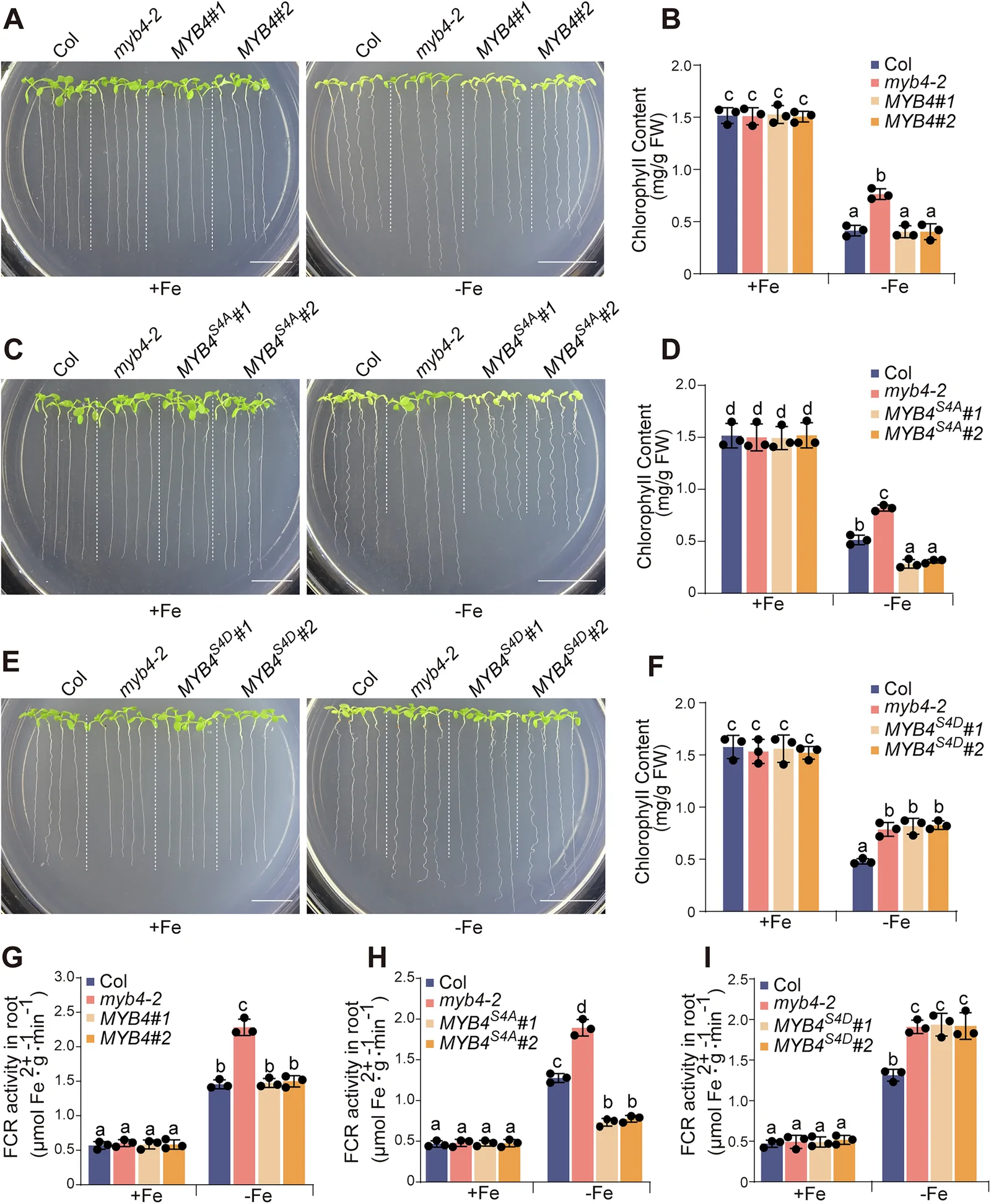
MPK3-mediated MYB4 phosphorylation modulates MYB4 function in the Fe-deficient signaling pathway. (**A**) Phenotypic analysis of Col, *myb4-2*, and *ProMYB4:MYB4-Flag/myb4-2* transgenic plants under Fe deficiency stress. Scale bar: 1 cm. (**B**) The total chlorophyll content of Col, *myb4-2*, and *ProMYB4:MYB4-Flag/myb4-2* transgenic plants under both normal and Fe deficiency stress conditions. Values shown are means ± SD (*n* = 3 biological replicates). Significance was determined using two-way ANOVA followed by Tukey’s test. *P* values >0.9999 (+Fe Col vs +Fe *myb4-2*), >0.9999 (+Fe Col vs +Fe *MYB4#1*), >0.9999 (+Fe Col vs +Fe *MYB4#2*), >0.9999 (+Fe *myb4-2* vs +Fe *MYB4#1*), >0.9999 (+Fe *myb4-2* vs +Fe *MYB4#2*), >0.9999 (+Fe *MYB4#1* vs +Fe *MYB4#2*), 0.0002 (-Fe Col vs -Fe *myb4-2*), >0.9999 (-Fe Col vs -Fe *MYB4#1*), >0.9999 (-Fe Col vs -Fe *MYB4#2*), 0.0002 (-Fe *myb4-2* vs -Fe *MYB4#1*), 0.0002 (-Fe *myb4-2* vs -Fe *MYB4#2*), >0.9999 (-Fe *MYB4#1* vs -Fe *MYB4#2*). (**C**) Tolerance analysis of Col, *myb4-2*, and *ProMYB4:MYB4*^*S4A*^*-Flag/myb4-2* transgenic plants to Fe deficiency stress. Scale bar: 1 cm. (**D**) The total chlorophyll content of Col, *myb4-2*, and *ProMYB4:MYB4*^*S4A*^*-Flag/myb4-2* transgenic plants under both normal and Fe deficiency stress conditions. Values shown are means ± SD (*n* = 3 biological replicates). Significance was determined using two-way ANOVA followed by Tukey’s test. *P* values >0.9999 (+Fe Col vs +Fe *myb4-2*), >0.9999 (+Fe Col vs +Fe *MYB4*^*S4A*^*#1*), >0.9999 (+Fe Col vs +Fe *MYB4*^*S4A*^*#2*), >0.9999 (+Fe *myb4-2* vs +Fe *MYB4*^*S4A*^*#1*), >0.9999 (+Fe *myb4-2* vs +Fe *MYB4*^*S4A*^*#2*), >0.9999 (+Fe *MYB4*^*S4A*^*#1* vs +Fe *MYB4*^*S4A*^*#2*), 0.0109 (-Fe Col vs -Fe *myb4-2*), 0.0328 (-Fe Col vs -Fe *MYB4*^*S4A*^*#1*), 0.0343 (-Fe Col vs -Fe *MYB4*^*S4A*^*#2*), <0.0001 (-Fe *myb4-2* vs -Fe *MYB4*^*S4A*^*#1*), <0.0001 (-Fe *myb4-2* vs -Fe *MYB4*^*S4A*^*#2*), >0.9999 (-Fe *MYB4*^*S4A*^*#1* vs -Fe *MYB4*^*S4A*^*#2*). (**E**) Fe deficiency stress tolerance analysis of Col, *myb4-2* and *ProMYB4:MYB4*^*S4D*^*-Flag/myb4-2* transgenic plants. Scale bar: 1 cm. (**F**) The total chlorophyll content of Col, *myb4-2*, and *ProMYB4:MYB4*^*S4D*^*-Flag****/****myb4-2* under both normal and Fe deficiency stress conditions. Values shown are means ± SD (*n* = 3 biological replicates). Significance was determined using two-way ANOVA followed by Tukey’s test. *P* values = 0.998 (+Fe Col vs +Fe *myb4-2*), >0.9999 (+Fe Col vs +Fe *MYB4*^*S4D*^*#1*), 0.9901 (+Fe Col vs +Fe *MYB4*^*S4D*^*#2*), 0.9999 (+Fe *myb4-2* vs +Fe *MYB4*^*S4D*^*#1*), >0.9999 (+Fe *myb4-2* vs +Fe *MYB4*^*S4D*^*#2*), >0.9999 (+Fe *MYB4*^*S4D*^*#1* vs +Fe *MYB4*^*S4D*^*#2*), 0.0084 (-Fe Col vs -Fe *myb4-2*), 0.0036 (-Fe Col vs -Fe *MYB4*^*S4D*^*#1*), 0.0027 (-Fe Col vs -Fe *MYB4*^*S4D*^*#2*), 0.9998 (-Fe *myb4-2* vs -Fe *MYB4*^*S4D*^*#1*), 0.9987 (-Fe *myb4-2* vs -Fe *MYB4*^*S4D*^*#2*), >0.9999 (-Fe *MYB4*^*S4D*^*#1* vs -Fe *MYB4*^*S4D*^*#2*). (**G-I**) Root FCR activity of Col, *myb4-2*, *ProMYB4:MYB4-Flag/myb4-2, ProMYB4:MYB4*^*S4A*^*-Flag/myb4-2*, and *ProMYB4:MYB4*^*S4D*^*-Flag/myb4-2* transgenic plants under both normal and Fe deficiency stress conditions. Values shown are means ± SD (*n* = 3 biological replicates). Significance was determined using two-way ANOVA followed by Tukey’s test. (**G**) *P* values = 0.9988 (+Fe Col vs +Fe *myb4-2*), >0.9999 (+Fe Col vs +Fe *MYB4#1*), >0.9999 (+Fe Col vs +Fe *MYB4#2*), >0.9999 (+Fe *myb4-2* vs +Fe *MYB4#1*), >0.9999 (+Fe *myb4-2* vs +Fe *MYB4#2*), >0.9999 (+Fe *MYB4#1* vs +Fe *MYB4#2*), <0.0001 (-Fe Col vs -Fe *myb4-2*), >0.9999 (-Fe Col vs -Fe *MYB4#1*), 0.9927 (-Fe Col vs -Fe *MYB4#2*), <0.0001 (-Fe *myb4-2* vs -Fe *MYB4#1*), <0.0001 (-Fe *myb4-2* vs -Fe *MYB4#2*), 0.9998 (-Fe *MYB4#1* vs -Fe *MYB4#2*). (**H**) *P* values >0.9999 (+Fe Col vs *myb4-2*), >0.9999 (+Fe Col vs +Fe *MYB4*^*S4A*^*#1*), >0.9999 (+Fe Col vs +Fe *MYB4*^*S4A*^*#2*), >0.9999 (+Fe *myb4-2* vs +Fe *MYB4*^*S4A*^*#1*), >0.9999 (+Fe *myb4-2* vs +Fe *MYB4*^*S4A*^*#2*), >0.9999 (+Fe *MYB4*^*S4A*^*#1* vs +Fe *MYB4*^*S4A*^*#2*), <0.0001 (-Fe Col vs -Fe *myb4-2*), <0.0001 (-Fe Col vs -Fe *MYB4*^*S4A*^*#1*), <0.0001 (-Fe Col vs -Fe *MYB4*^*S4A*^*#2*), <0.0001 (-Fe *myb4-2* vs -Fe *MYB4*^*S4A*^*#1*), <0.0001 (-Fe *myb4-2* vs -Fe *MYB4*^*S4A*^*#2*), 0.9755 (-Fe *MYB4*^*S4A*^*#1* vs -Fe *MYB4*^*S4A*^*#2*). (**I**) *P* values >0.9999 (+Fe Col vs +Fe *myb4-2*), >0.9999 (+Fe Col vs +Fe *MYB4*^*S4D*^*#1*), 0.9991 (+Fe Col vs +Fe *MYB4*^*S4D*^*#2*), >0.9999 (+Fe *myb4-2* vs +Fe *MYB4*^*S4D*^*#1*), >0.9999 (+Fe *myb4-2* vs +Fe *MYB4*^*S4D*^*#2*), >0.9999 (+Fe *MYB4*^*S4D*^*#1* vs +Fe *MYB4*^*S4D*^*#2*), <0.0001 (-Fe Col vs -Fe *myb4-2*), <0.0001 (-Fe Col vs -Fe *MYB4*^*S4D*^*#1*), <0.0001 (-Fe Col vs -Fe *MYB4*^*S4D*^*#2*), >0.9999 (-Fe *myb4-2* vs -Fe *MYB4*^*S4D*^*#1*), >0.9999 (-Fe *myb4-2* vs -Fe *MYB4*^*S4D*^*#2*), >0.9999 (-Fe *MYB4*^*S4D*^*#1* vs -Fe *MYB4*^*S4D*^*#2*). Source data are available online for this figure.

To investigate the biological significance of MYB4 phosphorylation by MPK3, we generated transgenic lines expressing wild-type and mutant MYB4 tagged with Flag under the control of its native promoter in the *myb4-2* mutant background (Appendix Fig. S3D–F). These included *ProMYB4:MYB4-Flag*, *ProMYB4:MYB4*^*S4A*^*-Flag* (phosphorylation-inactivated mutant), and *ProMYB4:MYB4*^*S4D*^*-Flag* (phosphorylation-mimic mutant). It was observed that *ProMYB4:MYB4-Flag/myb4-2* plants exhibited a Fe-deficiency phenotype similar to that of Col (Fig. 8A), whereas *ProMYB4:MYB4*^*S4A*^*-Flag/myb4-2* plants displayed a more sensitive Fe-deficiency phenotype compared with Col (Fig. 8C). In contrast, *ProMYB4:MYB4*^*S4D*^*-Flag/myb4-2* plants exhibited a Fe-deficiency tolerance phenotype similar to that of the *myb4-2* (Fig. 8E), as evidenced by their comparable total chlorophyll content (Fig. 8B,D,F) and FCR activity (Fig. 8G–I). Collectively, these results demonstrate that MPK3-mediated phosphorylation of MYB4 at Ser4 is essential for MYB4 function under Fe deficiency stress.

### MPK3-mediated phosphorylation at Ser4 attenuates the interaction between MYB4 and FIT

**Figure 9:**
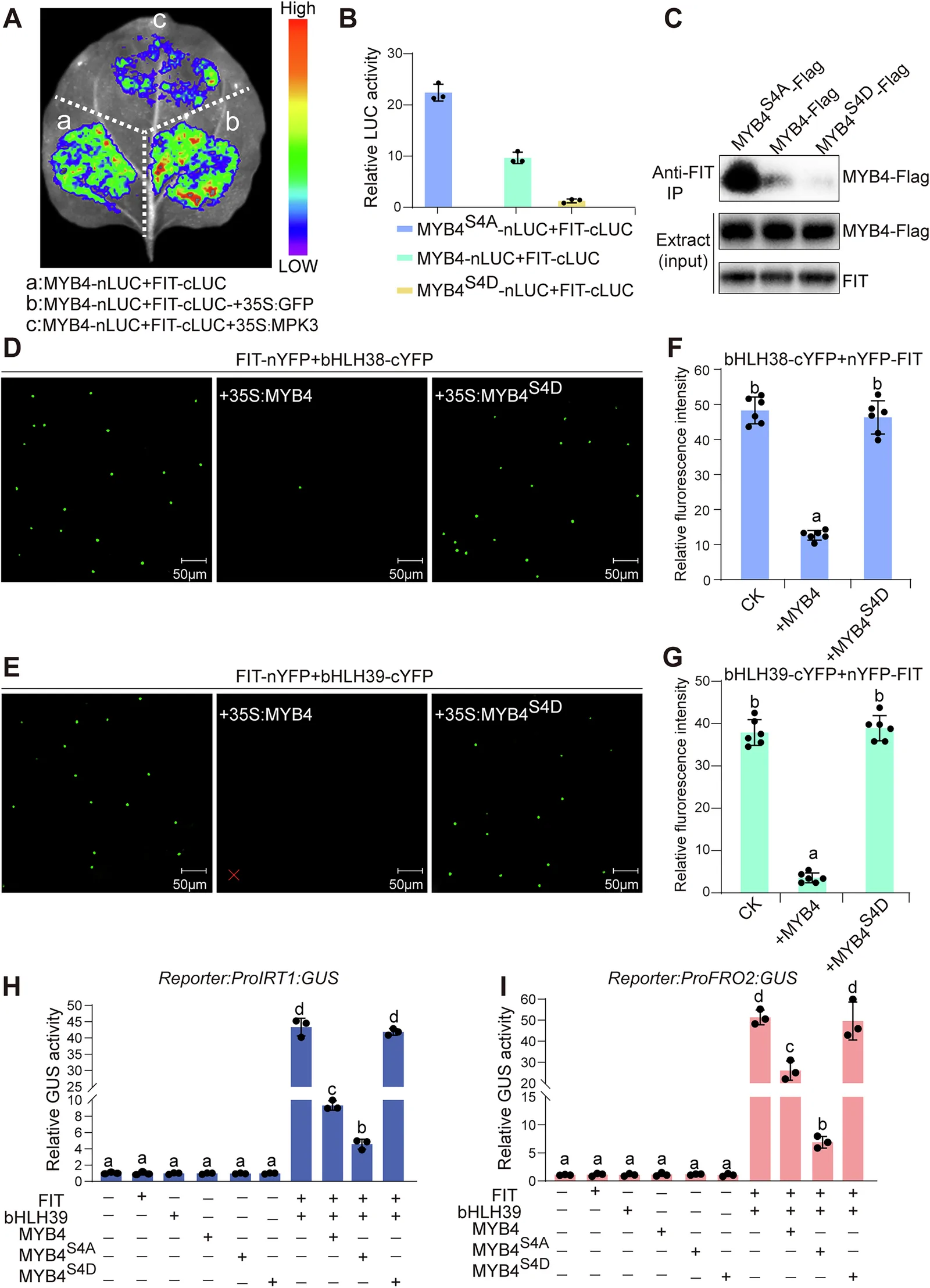
MPK3-mediated phosphorylation at Ser4 attenuates the interaction between MYB4 and FIT. (**A**) LCI assay showing the effect of MPK3 on the interaction between MYB4 and FIT in *N. benthamiana leaves*. (**B**) LCI assays demonstrate that phosphorylation of MYB4 at Ser4 attenuates its interaction with FIT in *N. benthamiana leaves*. Values shown are means ± SD (*n* = 3 biological replicates). Significance was determined using Ordinary one-way ANOVA followed by Tukey’s test. *P* values <0.0001 (MYB4^S4A^-nLUC+FIT-cLUC vs MYB4-nLUC+FIT-cLUC), <0.0001 (MYB4^S4A^-nLUC+FIT-cLUC vs MYB4^S4D^-nLUC+FIT-cLUC). (**C**) Co-IP assays verifying the interaction of three MYB4 versions with FIT in planta. Protein extracts from the indicated genotypes were immunoprecipitated using anti-FIT agarose beads. Input and immunoprecipitated proteins were detected by anti-FIT or anti-FLAG antibodies. (**D**,** E**) BiFC was employed to assess the effects of wild-type MYB4 and the phosphorylation-mimicking mutant MYB4^S4D^ on the interaction between FIT and either bHLH38 or bHLH39. FIT/bHLH38 and FIT/bHLH39 interaction were used as the positive control (CK). Scale bar: 50 μm. The red marking indicates that no fluorescence signal was detected. (**F**,** G**) Quantitative analysis of YFP fluorescence intensity. Fluorescence intensity was evaluated for six independent microscopic fields of view. Values shown are means ± SD (*n* = 6 biological replicates). Significance was determined using Ordinary one-way ANOVA followed by Tukey’s. (**F**) *P* values <0.0001 (FIT-nYFP+bHLH38-cYFP vs FIT-nYFP+bHLH38-cYFP+35S:MYB4), 0.5413 (FIT-nYFP+bHLH38-cYFP vs FIT-nYFP+bHLH38-cYFP+35S:MYB4^S4D^). (**G**) *P* values <0.0001 (FIT-nYFP+bHLH39-cYFP vs FIT-nYFP+bHLH39-cYFP+35S:MYB4), 0.7213 (FIT-nYFP+bHLH39-cYFP vs FIT-nYFP+bHLH39-cYFP+35S:MYB4^S4D^). (**H**,** I**) Relative GUS activity of the *ProIRT1:GUS* and *ProFRO2:GUS* reporter genes after expression of FIT, bHLH39, MYB4, MYB4^S4A^, or MYB4^S4D^ alone or in combination in *N. benthamiana leaves*. *35S*-empty vector was used as effector plasmid control. Values shown are means ± SD (*n* = 3 biological replicates). Significance was determined using Ordinary one-way ANOVA followed by Tukey’s. (**H**) *P* values >0.9999 (*ProIRT1*:GUS vs 35S:FIT*+ProIRT1*:GUS), >0.9999 (*ProIRT1*:GUS vs 35S: bHLH39+*ProIRT1*:GUS), >0.9999 (*ProIRT1*:GUS vs 35S:MYB4+*ProIRT1*:GUS), >0.9999 (*ProIRT1*:GUS vs 35S:MYB4^S4A^+*ProIRT1*:GUS), >0.9999 (*ProIRT1*:GUS vs 35S:MYB4^S4D^+*ProIRT1*:GUS), <0.0001 (*ProIRT1*:GUS vs 35S:FIT + 35S:bHLH39+*ProIRT1*:GUS), <0.0001 (*ProIRT1*:GUS vs 35S:FIT + 35S:bHLH39 + 35S:MYB4+*ProIRT1*:GUS), 0.0015 (*ProIRT1*:GUS vs 35S:FIT + 35S:bHLH39 + 35S:MYB4^S4A^+*ProIRT1*:GUS), <0.0001 (*ProIRT1*:GUS vs 35S:FIT + 35S:bHLH39 + 35S:MYB4^S4D^+*ProIRT1*:GUS), <0.0001 (35S:FIT + 35S:bHLH39+*ProIRT1*:GUS vs 35S:FIT + 35S:bHLH39 + 35S:MYB4+*ProIRT1*:GUS), <0.0001 (35S:FIT + 35S:bHLH39+*ProIRT1*:GUS vs 35S:FIT + 35S:bHLH39 + 35S:MYB4^S4A^+*ProIRT1*:GUS), 0.7117 (35S:FIT + 35S:bHLH39+*ProIRT1*:GUS vs 35S:FIT + 35S:bHLH39 + 35S:MYB4^S4D^+*ProIRT1*:GUS), 0.0002 (35S:FIT + 35S:bHLH39 + 35S:MYB4+*ProIRT1*:GUS vs 35S:FIT + 35S:bHLH39 + 35S:MYB4^S4A^+*ProIRT1*:GUS), <0.0001 (35S:FIT + 35S:bHLH39 + 35S:MYB4+*ProIRT1*:GUS vs 35S:FIT + 35S:bHLH39 + 35S:MYB4^S4D^+*ProIRT1*:GUS), <0.0001 (35S:FIT + 35S:bHLH39 + 35S:MYB4^S4A^+*ProIRT1*:GUS vs 35S:FIT + 35S:bHLH39 + 35S:MYB4^S4D^+*ProIRT1*:GUS). (**I**) *P* values >0.9999 (*Pro FRO2*:GUS vs 35S:FIT+*ProFRO2*:GUS), >0.9999 (*ProFRO2*:GUS vs 35S: bHLH39+*ProFRO2*:GUS), >0.9999 (*ProFRO2*:GUS vs 35S:MYB4+*ProFRO2*:GUS), >0.9999 (*ProFRO2*:GUS vs 35S:MYB4^S4A^+*ProFRO2*:GUS), >0.9999 (*ProFRO2*:GUS vs 35S:MYB4^S4D^+*ProFRO2*:GUS), <0.0001 (*ProFRO2*:GUS vs 35S:FIT + 35S:bHLH39+*ProFRO2*:GUS), <0.0001 (*ProFRO2*:GUS vs 35S:FIT + 35S:bHLH39 + 35S:MYB4+*ProFRO2*:GUS), 0.2533 (*ProFRO2*:GUS vs 35S:FIT + 35S:bHLH39 + 35S:MYB4^S4A^+*ProFRO2*:GUS), <0.0001 (*ProFRO2*:GUS vs 35S:FIT + 35S:bHLH39 + 35S:MYB4^S4D^+*ProFRO2*:GUS), <0.0001 (35S:FIT + 35S:bHLH39+*ProIRT1*:GUS vs 35S:FIT + 35S:bHLH39 + 35S:MYB4+*ProFRO2*:GUS), <0.0001 (35S:FIT + 35S:bHLH39+*ProIRT1*:GUS vs 35S:FIT + 35S:bHLH39 + 35S:MYB4^S4A^+*ProFRO2*:GUS), 0.9996 (35S:FIT + 35S:bHLH39+*ProIRT1*:GUS vs 35S:FIT + 35S:bHLH39 + 35S:MYB4^S4D^+*ProFRO2*:GUS), <0.0001 (35S:FIT + 35S:bHLH39 + 35S:MYB4+*ProIRT1*:GUS vs 35S:FIT + 35S:bHLH39 + 35S:MYB4^S4A^+*ProFRO2*:GUS), <0.0001 (35S:FIT + 35S:bHLH39 + 35S:MYB4+*ProIRT1*:GUS vs 35S:FIT + 35S:bHLH39 + 35S:MYB4^S4D^+*ProFRO2*:GUS), <0.0001 (35S:FIT + 35S:bHLH39 + 35S:MYB4^S4A^+*ProIRT1*:GUS vs 35S:FIT + 35S:bHLH39 + 35S:MYB4^S4D^+*ProFRO2*:GUS). Source data are available online for this figure.

Given that MYB4 undergoes phosphorylation (Fig. 7), we next investigated whether MPK3-mediated phosphorylation of MYB4 at Ser4 modulates its interaction with FIT. LCI assays revealed that the MYB4-FIT interaction was significantly attenuated upon coexpression with MPK3 (Fig. 9A). In addition, the dephosphorylation-mimicking mutant MYB4^S4A^ exhibited a significantly stronger interaction with FIT compared to native MYB4, whereas the phosphorylation-mimicking mutant MYB4^S4D^ showed a markedly reduced interaction with FIT (Fig. 9B). These data support the notion that phosphorylation of MYB4 at Ser4 attenuates its interaction with FIT. To further validate this observation, Co-IP assays were conducted, and Western blotting analysis revealed that MYB4^S4A^-HA interacts more strongly with FIT compared to MYB4-HA, whereas no interaction was detected between MYB4^S4D^-HA and FIT (Fig. 9C). Figure 3 shows that MYB4 can inhibit the formation of the FIT-bHLH38/39 complex. Therefore, we investigated whether phosphorylation at Ser4 affects the formation of the FIT-bHLH38/39 heterodimer using the BiFC assay. A strong YFP fluorescence signal could be observed when FIT-nYFP was expressed together with bHLH38/39-cYFP in *N. benthamiana* leaves (Fig. 9D–G); however, YFP fluorescence signals were significantly weaker when *35S:MYB4* and FIT-nYFP were co-expressed with bHLH38/39-cYFP in *N. benthamiana* leaves (Fig. 9D–G). Notably, when the phosphorylation-mimicking mutant *35S:MYB4*^*S4D*^ was co-expressed with FIT-nYFP and bHLH38/39-cYFP in *N. benthamiana* leaves (Fig. 9D–G), YFP fluorescence signals were significantly enhanced. Collectively, these data suggest that MPK3-mediated phosphorylation of MYB4 at Ser4 attenuates its interaction with FIT. To determine whether Ser4 phosphorylation directly affects the intrinsic binding ability of MYB4 toward FIT, we further performed in vitro pull-down assays using purified recombinant proteins. The results showed that wild-type MYB4, the dephosphorylation-mimicking mutant MYB4^S4A^, and the phosphorylation-mimicking mutant MYB4^S4D^ exhibited comparable binding capacities toward FIT, indicating that alteration of the Ser4 phosphorylation status does not directly impair the physical association between MYB4 and FIT. These findings suggest that the reduced interaction observed in planta is more likely attributed to phosphorylation-dependent changes in MYB4 protein abundance or stability rather than altered intrinsic binding affinity (Fig. EV2D).

Furthermore, we investigated whether MPK3-mediated phosphorylation of MYB4 at Ser4 modulates the transcriptional activity of the FIT/bHLH39 complex on its downstream target genes, *IRT1* and *FRO2*. To this end, transient transcriptional activation assays were performed in *N. benthamiana* leaves. Expression of FIT, bHLH39, MYB4, MYB4^S4A^, or MYB4^S4D^ alone did not alter the activity of *ProFRO2:GUS* or *ProIRT1:GUS* (Fig. 9H,I). In contrast, coexpression of FIT and bHLH39 significantly enhanced the reporter activity of both *ProIRT1:GUS* and *ProFRO2:GUS*. Coexpression of wild-type MYB4 with FIT and bHLH39 markedly suppressed this activation, indicating that MYB4 represses the transcriptional activity of the complex. Notably, the dephosphorylation-mimicking mutant MYB4^S4A^ exhibited a stronger repressive effect than wild-type MYB4, while the phosphorylation-mimicking mutant MYB4^S4D^ showed a significantly reduced repression, resulting in higher reporter activity (Fig. 9H,I). These preliminary results in *N. benthamiana* suggest that phosphorylation of MYB4 at Ser4 may impair its ability to inhibit FIT/bHLH39-mediated transcription of *IRT1* and *FRO2*.

## Discussion

Fe acquisition in *Arabidopsis* relies on the tightly controlled activity of the central transcription factor FIT, whose function is modulated through dynamic interactions with both positive and negative regulators (Gao et al, 2021). In this study, we identify MYB4 as a previously unrecognized component of this regulatory network. Unlike previously characterized MYB proteins involved in Fe homeostasis, such as MYB10/MYB72 that positively regulate Fe mobilization and nicotianamine-associated responses, and MYB30 positively regulate Fe homeostasis by maintaining the stability of the FIT, MYB4 functions as a negative regulator that directly associates with FIT and restricts the formation of transcriptionally active FIT-bHLH complexes (Palmer et al, 2013; Wu et al, 2024; Zhao et al, 2025).

Increasing evidence indicates that FIT activity is extensively fine-tuned at the post-translational level through competitive protein interactions and regulated protein turnover. Previous studies identified bHLH18/19/20/25 (Cui et al, 2018), DELLA proteins (Wild et al, 2016), ZAT12 (Le et al, 2016), FBP (Chen et al, 2018), and MYC1 (Song et al, 2023) as negative regulators of FIT activity through distinct mechanisms. Our findings add MYB4 to this growing regulatory framework and further support the emerging concept that repression of FIT activity is actively required to balance Fe acquisition and prevent excessive Fe accumulation.

MYB4 belongs to the R2R3-MYB transcription factor family and has been implicated in a wide range of biological processes. Previous studies demonstrated that MYB4 negatively regulates vascular lignification by repressing lignin biosynthesis, a process modulated through MAPK-dependent phosphorylation and dephosphorylation by MKP1 (Lin et al, 2022). In addition, MYB4 directly activates MAN3 expression through the glutathione-dependent phytochelatin biosynthetic pathway and thereby enhances cadmium tolerance (Yan et al, 2021). MYB4 has also been reported to promote cadmium detoxification by inducing the expression of MT1C and PCS1 and alleviating oxidative damage (Agarwal et al, 2020). These studies collectively establish MYB4 as a multifunctional transcription factor involved in diverse stress response and metabolic pathways. Given these previously reported functions, it remained possible that MYB4 might influence Fe homeostasis through regulatory pathways beyond the FIT module. To address this possibility, we examined the expression of two well-characterized MYB4 target genes, *ADT6* and *MAN3*, under both Fe-sufficient and Fe-deficient conditions. Consistent with previous reports, *ADT6* expression was elevated, whereas *MAN3* expression was reduced in the *myb4* mutant relative to wildtype (Appendix Fig. S3G,H), confirming that *MYB4* retains its established transcriptional regulatory activities under Fe-deficient conditions. However, the expression patterns of these genes did not correlate with the enhanced Fe acquisition phenotype observed in *myb4* mutants, suggesting that their regulation is unlikely to account for the altered Fe-deficiency response. We further assessed whether *MYB4* participates more broadly in micronutrient deficiency responses. Notably, *myb4-1* mutants did not exhibit obvious phenotypic alterations under manganese-, copper-, or zinc-deficient conditions (Appendix Fig. S1). In contrast, the mutant consistently displayed enhanced tolerance to Fe deficiency, accompanied by increased expression of the FIT target genes *IRT1* and *FRO2*, elevated ferric chelate reductase activity, and increased Fe accumulation. Together with our genetic and biochemical evidence demonstrating that MYB4 regulates FIT-bHLH complex formation, these findings support a model in which the predominant detectable function of MYB4 in Fe homeostasis is mediated through the FIT-dependent Fe uptake pathway. Nevertheless, we cannot exclude the possibility that MYB4 indirectly influences additional Fe-responsive genes or signaling processes. Future transcriptomic and genome-wide target identification studies will be valuable for defining the broader MYB4-dependent regulatory network during Fe deficiency. Previous studies reported that MYB7 and MYB32 and their homolog MYB4 regulate flavonoid biosynthesis in Arabidopsis by interfering with the transcriptional activity of the MBW complex through interactions with TT8, GL3, and EGL3 transcription factors (Wang et al, 2020). Here, we found that the MYB4 homologs MYB7 and MYB32 did not interact with FIT by Y2H analyses (Appendix Fig. S2B), but the roles of MYB7 and MYB32 in Fe homeostasis in *Arabidopsis* are also worthy of further investigation, as they may have redundant or different functions.

Multiple repressors have been reported to attenuate FIT activity under Fe-deficient conditions, highlighting the necessity for multilayered control of Fe uptake. DELLA proteins, FBP, ZAT12, and MYC1 all negatively influence FIT function through protein–protein interactions or interference with FIT-bHLH complex formation (Le et al, 2016; Wild et al, 2016; Chen et al, 2018; Cui et al, 2018; Song et al, 2023). Our data suggest that MYB4 operates through a mechanistically distinct mode. Unlike DELLAs, MYB4 does not associate with Ib bHLH proteins, but instead competes directly with bHLH38/39 for binding to the FIT-M region. This competitive interaction provides a mechanistic explanation for the reduced DNA-binding activity and transcriptional output of FIT-bHLH complexes observed in the presence of MYB4. These findings reinforce the idea that FIT functions as a highly dynamic signaling hub whose activity is determined by the balance between activating and inhibitory interactors. Such a regulatory architecture may allow plants to rapidly adjust Fe uptake capacity in response to fluctuating environmental Fe availability while minimizing the risk of Fe-induced oxidative damage.

Post-translational phosphorylation has emerged as a central mechanism for rapidly rewiring stress-responsive transcriptional networks in plants. Several MYB transcription factors have previously been identified as MAPK substrates, with phosphorylation influencing either their stability or transcriptional activity (Li et al, 2016; Kim et al, 2017; Im et al, 2021). Our study extends this regulatory paradigm to Fe homeostasis by identifying MYB4 as a direct substrate of MPK3. Mechanistically, MPK3-mediated phosphorylation of MYB4 at Ser4 promotes its ubiquitin-dependent degradation and weakens its interaction with FIT, thereby relieving repression of FIT-bHLH transcriptional complexes under Fe-deficient conditions. This mechanism establishes a phosphorylation-dependent molecular switch that dynamically couples Fe-deficiency signaling to transcriptional activation of Fe uptake genes. Although MPK3 and MPK6 often function redundantly in multiple stress pathways, our genetic and biochemical analyses support a predominant role for MPK3 in regulating MYB4-dependent Fe responses. These findings suggest that Fe homeostasis may utilize a more specialized MAPK signaling branch than previously appreciated.

MPK3-mediated phosphorylation at Ser4 serves as a central regulatory switch controlling MYB4 stability and repressive activity during the Fe-deficiency response. Under both normal and Fe-deficient conditions, the dephosphorylation-mimicking MYB4^S4A^-Flag exhibits greater stability than MYB4-Flag, whereas the phosphorylation-mimicking MYB4^S4D^-Flag shows the opposite trend (Fig. 7G). Fe-deficient conditions markedly decreased MYB4 abundance and promoted the accumulation of polyubiquitinated MYB4 species, whereas the phospho-inactive MYB4^S4A^ variant remained stable and exhibited reduced ubiquitination, indicating that Ser4 phosphorylation is required for directing MYB4 to the ubiquitin-proteasome pathway, consistent with phosphorylation-dependent turnover mechanisms reported for ICE1 and several MYB transcription factors (Li et al, 2017; Nguyen et al, 2012; Kim et al, 2017). The contrasting phenotypes of MYB4^S4A^ and MYB4^S4D^ lines further highlight the functional relevance of this modification: persistent MYB4^S4A^ repression exacerbates Fe-deficiency sensitivity, whereas accelerated turnover of MYB4^S4D^ confers enhanced tolerance. Together with our domain-mapping evidence showing that MYB4 competitively occupies the FIT-M region required for bHLH38/39 recruitment, these results support a unified model in which Fe deficiency activates MPK3 (Ye et al, 2015), thereby triggering MYB4 phosphorylation, its dissociation from FIT, and subsequent degradation. This phosphorylation-ubiquitination cascade effectively relieves MYB4-mediated inhibition of FIT-bHLH complex assembly and aligns with established proteolytic control of FIT and bHLH IVc regulators in Fe homeostasis (Selote et al, 2015; Rodríguez-Celma et al, 2019; Gao et al, 2020), enabling rapid transcriptional activation of Fe-uptake genes under Fe-limiting conditions.

Previous studies have shown that some transcription factors are modified by ubiquitination after they undergo translation, and their proteins are usually tagged with ubiquitination and degraded via the 26S/ubiquitin-proteasome pathway (Desterro et al, 2000; Vierstra, 2009). Here, our data show that MG132 inhibits MYB4 protein degradation (Appendix Fig. S1C,D) and that MYB4 protein accumulation is significantly reduced by Fe deficiency stress (Fig. 3H), so we speculate that MYB4 stability is regulated by the ubiquitin-proteasome system (UPS). Further, we also used an anti-ubiquitin antibody to detect ubiquitination in phospho-inactive *Pro:MYB4:MYB4*^*S4A*^*-Flag* transgenic plants and found that the phospho-inactive MYB4^S4A^-Flag attenuated the polyubiquitination and degradation of MYB4 under Fe deficiency stress (Appendix Fig. S3C). Degradation assays and ubiquitination analyses suggest phosphorylation status influences MYB4 stability via the ubiquitin-proteasome system. Although our findings establish a clear functional connection between MPK3-dependent phosphorylation and MYB4 turnover, the molecular machinery responsible for this process remains to be identified. In particular, the E3 ubiquitin ligase(s) that recognize phosphorylated MYB4 and mediate its ubiquitination were not identified in the present study. Therefore, our work defines a phosphorylation-dependent mechanism controlling MYB4 stability, while the detailed ubiquitination pathway underlying MYB4 degradation awaits further investigation.

In conclusion, we identified MYB4 as an additional regulator of FIT activity and provided evidence that MPK3-mediated regulation of MYB4 stability contributes to the dynamic modulation of Fe-deficiency responses in Arabidopsis. Under Fe-sufficient conditions, MYB4 is moderately expressed and competes with bHLH38/39 for binding to FIT, thereby inhibiting the formation of functional FIT-bHLH38/39 heterodimers. As a result, the transcriptional activities of Fe-uptake genes *IRT1* and *FRO2* are inhibited, maintaining Fe homeostasis in plants under normal physiological conditions. Under Fe deficiency, MPK3 activation triggers MYB4 phosphorylation and subsequent degradation, thereby releasing FIT to form functional heterodimers with bHLH38/39 and activate Fe-uptake genes, ultimately enhancing plant tolerance to Fe deficiency stress. Such a reversible system enables plants to rapidly adjust Fe uptake while maintaining homeostatic control, thereby preventing both Fe deficiency and Fe toxicity. This work enhances our understanding of how MAPK signaling integrates environmental cues into micronutrient regulatory networks. While this research has largely elucidated the regulation of Fe deficiency stress in plants by the MPK3-MYB4-FIT cascade, several questions remain unresolved. How MPK3 senses Fe status and thereby regulates MYB4 phosphorylation remains an important question. The molecular basis underlying the specificity of MYB4 recognition requires further investigation. Additionally, whether MPK3, which is involved in Fe homeostasis, has additional substrates warrants further exploration. These questions will be addressed in future studies. Taken together, our study expands the current FIT-centered regulatory framework by identifying a phosphorylation-responsive competitive repressor mechanism that dynamically modulates FIT activity during Fe deficiency adaptation.

## Methods

**Table:** Reagents and tools table

Reagent/resource	Reference or Source	Identifier or Catalog Number
Arabidopsis: Col-0	Widely distributed	N/A
Arabidopsis: Ler	Landsberg erecta	N/A
Arabidopsis: *myb4-2*	Wang et al, 2020	N/A
Arabidopsis: *myb4-1*	Yan et al, 2021	N/A
Arabidopsis: *fit*	Song et al, 2023	N/A
Arabidopsis: *mpk3-1*	Li et al, 2017	N/A
Arabidopsis: *mpk3-2*	Li et al, 2017	N/A
Arabidopsis: *fit/myb4-2*	This study	N/A
Arabidopsis: *mpk3-1/myb4-2*	This study	N/A
Arabidopsis: *proMYB4:MYB4-Flag/myb4-2*	This study	N/A
Arabidopsis: *proMYB4:MYB4*^*S4A*^*-Flag/myb4-2*	This study	N/A
Arabidopsis: *proMYB4:MYB4*^*S4D*^*-Flag/myb4-2*	This study	N/A
Arabidopsis: *35S:MYB4-GFP*	This study	N/A
Arabidopsis: *35S:MYB4-GFP/mpk3-1*	This study	N/A
**Recombinant DNA**	This study	
*pGADT7-FIT*	This study	N/A
*pGADT7-FIT-N*	This study	N/A
*pGADT7-FIT-M*	This study	N/A
*pGADT7-FIT-C*	This study	N/A
*pGADT7-MYB4*	This study	N/A
*pGBKT7-MYB4*	This study	N/A
*pGADT7-MPK3*	This study	N/A
*pGADT7-bHLH38*	This study	N/A
*pGBKT7-bHLH38*	This study	N/A
*pGADT7-bHLH39*	This study	N/A
*pGBKT7-bHLH39*	This study	N/A
*pGADT7-bHLH100*	This study	N/A
*pGADT7-bHLH101*	This study	N/A
*pGBKT7-MYB7*	This study	N/A
*pGBKT7-MYB32*	This study	N/A
*pBTEX-MYB4-HA*	This study	N/A
*pBTEX-FIT-Flag*	This study	N/A
*pBTEX-MPK3-Flag*	This study	N/A
*pBSPYCE-MYB4-cYFP*	This study	N/A
*pBSPYCE-FIT-nYFP*	This study	N/A
*pBSPYCE-MPK3-nYFP*	This study	N/A
*pBSPYCE-bHLH38-cYFP*	This study	N/A
*pBSPYCE-bHLH39-cYFP*	This study	N/A
*pCAMBIA1300-35S-FIT-cLUC*	This study	N/A
*pCAMBIA1300-35S-MYB4-cLUC*	This study	N/A
*pCAMBIA1300-35S-MYB4* ^*S4A*^ *-cLUC*	This study	N/A
*pCAMBIA1300-35S-MYB4* ^*S4D*^ *-cLUC*	This study	N/A
*ProIRT1-*placZi	This study	N/A
*ProFRO2-*placZi	This study	N/A
PJG4-5-MYB4	This study	N/A
*pART27-35S-proIRT1-GUS*	This study	N/A
*pART27-35S-proFRO2-GUS*	This study	N/A
*pART27-35S-MYB4-GFP*	This study	N/A
*pART27-35S-FIT-GFP*	This study	N/A
*pART27-35S-bHLH39-GFP*	This study	N/A
*pCold-His-MYB4*	This study	N/A
*pCold-His-FIT*	This study	N/A
*pCold-His-bHLH39*	This study	N/A
*pMAL-c2-MKK5* ^*DD*^ *-MBP*	This study	N/A
*pMAL-c2-MBP3-MBP*	This study	N/A
*pGEX-4T-3-MYB4-GST*	This study	N/A
*pGEX-4T-3-MYB4* ^*S4A*^ *-GST*	This study	N/A
*pGEX-4T-3-MYB4* ^*T221A*^ *-GST*	This study	N/A
*pGEX-4T-3-MYB4* ^*2A*^ *-GST*	This study	N/A
*pCAMBIA1305-proMYB4-MYB4-Flag*	This study	N/A
*pCAMBIA1305-proMYB4-MYB4* ^*S4A*^ *-Flag*	This study	N/A
*pCAMBIA1305-proMYB4-MYB4* ^*S4D*^ *-Flag*	This study	N/A
*pBridge-BD- bHLH38-MYB4*	This study	N/A
*pBridge-BD- bHLH39-MYB4*	This study	N/A
*pBridge-BD- bHLH38-MBP*	This study	N/A
*pBridge-BD- bHLH39-MBP*	This study	N/A
**Antibodies**		
Anti-Actin	Abclonal	Cat#AC009
Anti-GFP antibody	Abmart	Cat#M20045
Anti-Flag antibody	Solarbio	Cat#K20000M
Anti-HA antibody	Abmart	Cat#M20003S
Anti-MPK3 antibody	PhytoAB	Cat#PHY1917A
Anti-p44/p42 MAPK	Cell Signaling	Cat# 9101
Anti-MBP antibody	Abmart	Cat#M20051S
Anti-GST antibody	Abmart	Cat#M20007S
Anti-Phosphoserine	Abcam	Cat#ab9332
Anti-FIT antibody	PhytoAB	Cat#PHYO939A
Anti-Ub antibody	Santa Cruz Biotechnology	Cat#SC8017
Anti-Rabbit	Abmart	Cat#M21001S
Anti-Mouse	Abmart	Cat#M21003S
**Oligonucleotides and other sequence-based reagents**		
yeast two-hybrid primers *EcoR I-FIT-F:* GGAATTCCATATGATGGAAGGAAGAGTCAACGCTCTGTCAA	This study	See Appendix Table S1 for details.
yeast two-hybrid primers *EcoR I-FIT-R:* CGGGATCCTCAAGTAAATGACTTGATGAATTCAAAA	This study	See Appendix Table S1 for details.
Other primers see Table S1	This study	See Appendix Table S1 for details.
**Chemicals, enzymes and other reagents**		
Anti-Flag Beads	Beyotime	Cat:P2115
Anti-GFP Beads	Beyotime	Cat:P2132
Ni NTA Beads 6FF	Solarbioe	Cat:P2010
Dextrin Beads 6FF	LABLEAD	Cat:M1050
GST-Sefinose Resin 4FF	Sangon Biotech	Cat:C6C0031-0005
MG132	Sangon Biotech	Cat:SP14421
D-Luciferin	GoldBio	Cat:017909LUCK
IPTG	Biofroxx	Cat:1122GR005
MES	Sangon Biotech	Cat:A610341-0100
PMSF	Aladdin	Cat:G1823053
**Software**		
ImageJ	National Institutes of Health	https://ImageJ.nih.gov/ij/
GraphPad Prism 10	GraphPad Software Inc.	https://www.graphpad.com
**Other**		
*Echerichia coli (E. coli)* strain BL21(DE3)	Lab stock	N/A
*E.coli* (DH5α)	Lab stock	N/A
*Agrobacterium tumefaciens* (strain GV3101)	Lab stock	N/A
Yeast strain (AH109)	Lab stock	N/A

### Plant genotypes and growing conditions

Mutants and transgenic lines were obtained from Landsberg erecta (Ler-0; *myb4-1 [CS26404]*; *MYB4-COM*), or Columbia (Col-0; *myb4-2 [GT8121.DS5]*; *MYB4-OE*; *fit* (SALK_144551); *mpk3-1* (SALK_151594); *mpk3-2* (SALK_100651) ecotypes as previously described (Li et al, 2017; Wang et al, 2020; Yan et al, 2021; Song et al, 2023). The *myb4* mutant was originally isolated in the Landsberg erecta (Ler) background (Wang et al, 2020). To generate a corresponding mutant in the Col-0 background, the *myb4* allele was backcrossed with wild-type Col-0 plants for five consecutive generations. The resulting *myb4-2* line carrying a predominantly Col-0 genetic background was used for all physiological, molecular, and genetic analyses in this study. Transgenic plants expressing *proMYB4:MYB4-Flag/myb4-2*, *proMYB4:MYB4*^*S4A*^*-Flag/myb4-2*, *proMYB4:MYB4*^*S4D*^*-Flag/myb4-2*, *35S:MYB4-GFP/Col*, and *35S:MYB4-GFP/mpk3-1* in Col, *myb4-2*, and *mpk3-1* background were generated in this work. The *fit/myb4-2* and *mpk3-1/myb4-2* double mutant plants were produced by hybridizing the *fit* or *mpk3-1* mutant with the *myb4-2* mutant as the female parent. Appendix Table S1 lists the primers used for genotyping.

For phenotypic analysis of Fe-deficiency responses, Arabidopsis seeds were surface-sterilized with 0.1 M mercuric chloride for 1 min, rinsed five times with double-distilled water, and subsequently germinated on either Fe-sufficient (+Fe) or Fe-deficient (−Fe) Murashige and Skoog (MS) medium. The Fe-deficient medium was prepared using MS salts lacking Fe-EDTA and supplemented with 5 mM ferrozine to chelate residual Fe potentially present in the agar, thereby minimizing the influence of Fe contamination on Fe-deficiency phenotypes. The pH of all media was adjusted to 5.8 prior to autoclaving. Culture plates were vernalized in a dark environment at 4 °C for 3 days and then moved to a controlled culture chamber at 22 °C for 10 days of growth before being photographed and tested for chlorophyll content.

### LCI assay

The LCI analysis was conducted following the previously established methodology (Xu et al, 2024). The coding sequences (CDS) of *MYB4*, *MYB4*^*S4A*^, *MYB4*^*S4D*^ and *FIT* were fused to the N-terminus or C-terminus of luciferase, respectively. The constructs were transformed into *Agrobacterium* and co-infiltrated into the leaves of 4-week-old *N. benthamiana* plants. Signals were detected using a low-light cooled charge-coupled device imaging system (Tanon 5200) 40 to 48 h after cultivation.

### Measuring plant chlorophyll content

Arabidopsis seedlings grown for 10 days on +Fe medium and Fe-deficient media were collected and weighed, and then extracted with 80% acetone solution for 12 h at 37 °C with occasional shaking in the dark. The supernatant was measured spectrophotometrically at 645 nm and 663 nm, and the chlorophyll content was calculated according to the previous method (Song et al, 2023).

### Measurement of Fe-chelate reductase activity

Yi et al (1996) provided a detailed description of the Fe-chelate reductase activity assay performed in this work. Using purple Fe^2+^-Ferrozine complexes spectrophotometrically to measure FCR activity in Arabidopsis roots. Arabidopsis seedlings grown on +Fe medium for 7 days were transferred to -Fe medium containing both 50 mM ferrozine +Fe medium and -Fe medium, and intact seedlings were collected after 3 days. The supernatants of the purple Fe^2+^-Ferrozine complexes were then analyzed spectrophotometrically at 562 nm. Ultimately, a molecular extinction coefficient of 28.6 mM^−1^ cm^−1^ was used to calculate FCR activity.

### RNA extract and RT-qPCR assay

Use Trizol reagent (Invitrogen) in accordance with the manufacturer’s instructions for extracting total plant RNA. A Nanodrop 2000 spectrophotometer was used to measure the concentration and purity of the extracted total RNA. A Nanodrop 2000 spectrophotometer was used to measure the purity and concentration of the extracted total RNA. The first strand of cDNA is produced from DNase-treated RNA in a 20 µl reaction using reverse transcriptase. RT-qPCR analyses were performed after setting up the program on a LightCycler 480 Real-Time PCR instrument (Roche) according to the SYBR Green manufacturer’s instructions. In the test using the *ACTIN8* gene as an internal reference. Appendix Table S1 lists the primers used in the RT-qPCR analysis.

### Transient expression assay

Song et al (2023) described in detail the transient expression assay implemented in *N. benthamiana* leaves. Inject *Agrobacterium* in the desired assay combination into the leaves of *N. benthamiana*. A fluorescent 4-methylumbelliferyl-b-D-glucuronide assay was used to detect *β*-glucuronidase (GUS) enzyme activity 48 h after injection. Primers utilized in this experiment are presented in Appendix Table S1.

### Y2H assay

To screen the cDNA libraries, the full-length *MYB4*-encoding fragment or the *FIT*-encoding fragment (100–318 bp) was cloned into the pGBKT7 bait vector, followed by transformation into the yeast strain AH109 (Clontech) and then into the cDNA library (Chen et al, 2018). The yeast transformants were screened in SD/-Leu/-His/-Trp (SD-LWH) containing 20 mM 3-AT selective media. For the detection of protein–protein interactions, full-length CDS fragments of truncated FIT and MYB4 were cloned into pGADT7, and full-length CDS fragments of *MYB4* and *MPK3* were cloned into pGBKT7. Primers utilized in this experiment are presented in Appendix Table S1.

### Bimolecular fluorescence complementation assay

For the BiFC assay, the full-length CDS fragment of *MYB4* was cloned into the pBSPYCE vector to obtain MYB4-cYFP, and full-length CDS fragments of *FIT* or *MPK3* were cloned into pBPYNE vector to obtain FIT-nYFP or MPK3-nYFP. Afterwards, the recombinant vector was transformed into *Agrobacterium* strain GV3101. Following this, *Agrobacterium* tumefaciens in various combinations of experiments were injected into *N. benthamiana* leaf blades for transient expression. Fluorescence signals of *N. benthamiana* leaf blades were observed and photographed using a confocal microscope after 36–48 h. Primers utilized in this experiment are presented in Appendix Table S1.

### Co-immunoprecipitation assay

The method of Co-IP analysis was as described previously (Song et al 2023). The full-length CDS fragment of *MYB4* was recombined into the pBTEX-HA vector to obtain MYB4-HA, and the full-length CDS fragments of *FIT* or *MPK3* were recombined into the pBTEX-Flag vector to obtain FIT-Flag or MPK3-Flag. The recombinant plasmid was then transformed into *Agrobacterium* strain GV3101. Thereafter, different experimental combinations of *Agrobacterium* tumefaciens GV3101 were injected into *N. benthamiana* leaves for transient expression. After 48 h, leaf proteins of *N. benthamiana* were extracted, 80 µl of protein supernatant was used as input and the rest of the protein supernatant was incubated with anti-Flag agarose beads at 4 °C for 6 h to capture Flag-tagged proteins. Immunoprecipitated proteins were analyzed by western blotting using anti-Flag or anti-HA antibodies.

### Chromatin immunoprecipitation assay

The ChIP assay was slightly modified according to the method of Wu et al (2024). The Arabidopsis materials used for the ChIP assay were all grown on +Fe medium for 7 d and then transplanted to +Fe and −Fe media for 3 d. Extracted and fragmented chromatin from these plant materials was then subjected to IP with anti-FIT (PhytoAB, California, USA) to obtain DNA–protein complexes. ChIP-DNA fragments were produced by uncrosslinking and were detected by RT-qPCR. In the test using the *ACTIN2* gene as an internal reference. Primers used in ChIP-qPCR are shown in Appendix Table S1.

### Yeast one- and three-hybrid assays

According to the experimental requirements, different combinations of the pLacZi reporter plasmid and the PJG4-5 fusion plasmid were transferred into the EGY48 yeast strain (Clontech) for the Y1H assay. The co-transformed yeast strains were incubated on SD/-Ura/-Trp culture plates containing X-gal in order to obtain a blue visual effect. Using the Y2H system, Y3H experiments were performed and were introduced as mentioned previously to express a third MYB4 (Zhu et al, 2022). The CDS sequence of *FIT* was recombined as prey to the pGADT7 construct, while the CDS sequence of *bHLH38* or *bHLH39* was recombined as bait to the pBridge construct. The CDS sequences of *MYB4* or MBP were recombined to the bHLH38-pBridge-link or bHLH39-pBridge-link constructs as a third decoy insert. Transform different experimental combinations into the AH109 yeast strain (Clontech). Transformed yeast strains were selected on SD/-Leu/-Trp medium and streaked onto SD/-His/-Trp/-Leu/-Ade and SD/-His/-Trp/-Leu/-Ade/-Met culture plates. Primers utilized in this experiment are presented in Appendix Table S1.

### Electrophoresis mobility shift assay

Thermo Fisher Scientific kits were used with the EMSA assay and performing the test according to the manufacturer’s instructions. Biotin-labeled *IRT1* and *FOR2* oligonucleotide probes were synthesized for use in the EMSA assay. His-tagged constructs MYB4, FIT, bHLH38 or bHLH39 were subjected to protein expression in *E. coli*, followed by purification of His-tagged fusion proteins using Amylose Purification. The oligonucleotides used in all binding sequences used herein are listed in Appendix Table S1.

### Fe content measurements

Separate roots and shoots of Arabidopsis seedlings grown on +Fe and Fe-deficient media for 10 days were collected to measure the Fe content. Afterward, 5 mM CaCl_2_ aqueous solution was used to wash the root samples while deionised water was used to wash the shoot samples. Tissue samples were weighed and dried at 80 °C in an oven for 2 days. Subsequently, 30% nitric acid and 10% perchloric acid were used to digest the tissue samples and an inductively coupled plasma mass spectrometer was applied to determine the Fe content (Song et al, 2022). Each sample used three biological replicates.

### Determination of MAPK Kinase Activity

Preparation of MAPK extraction buffer [100 mM HEPES, pH 8.0, 5 mM EGTA, 5 mM EDTA, 10 mM NaF, 10 mM Na_3_VO_4_, 10 mM dithiothreitol, 50 mM β-glycerophosphate, 1 mM phenylmethylsulfonyl fluoride, 5% glycerol, 5 g ml^−1^ aprotinin, and 5 g ml^−1^ leupeptin], and then Col proteins under normal and Fe deficiency treated using MAPK extraction buffer. anti-phospho-p44/42 MAPK antibody (Cell Signaling Technology, Massachusetts, USA) is used to detect MAPK kinase activity.

### Phosphorylation analysis in vivo

For in vivo phosphorylation assays of the MYB4 protein, MYB4-FLAG seedlings were grown vertically on Fe-sufficient agar plates for 10 days and then transferred to either Fe-sufficient (+Fe, control) or Fe-deficient (−Fe, without Fe) media for 3 days. Total proteins were extracted and immunoprecipitated using anti-FLAG agarose beads for 6 h, followed by digestion without or with calf intestinal phosphatase (CIP) at 30 °C for 40 min. The resulting samples were then detected by anti-Phosphoserine (Abcam, ab9332) and anti-Flag antibodies, respectively.

### In vitro phosphorylation assay

The non-radioactive ATP-gamma/γ-S in vitro phosphorylation assay was conducted in accordance with the method of Xu et al, (2024) with small modifications. MKK5^DD^-MBP, MPK3-MBP, MYB4-GST, MYB4^S4A^-GST, MYB4^T221A^-GST, and MYB4^2A^-GST vectors referred to in this assay were transformed to BL21 with expression and purification of recombinant proteins. The 1 mM ATP-γ-S (Abcam, Cambridge, UK) and kinase buffer (0.05% Triton X-100, 50 mM MgCl_2_, 50 mM KCl, 100 mM HEPES, PH 7.5) were co-added to the purified fusion proteins with 20 µl total volume. The reaction solution was incubated at 37 °C for 40 min, then incubated for an additional 2 h with 2.5 mM p-nitrobenzyl mesylate Alkylation (Abcam, Cambridge, UK). Anti-Thiophosphate ester (Abcam, Cambridge, UK) antibody is used for immunoblotting to detect phosphorylated bands.

### Cell-free protein degradation assay

Total proteins were extracted using in degradation buffer [10 mM MgCl_2_, 10 mM NaCl, 5 mM DTT, 4 mM PMSF, 10 mM ATP, and 25 mM Tris-HCl (pH 7.5)]. Afterwards, Bio-Rad protein assay was performed to measure the protein concentration of the extracts. The extract was then incubated with 100 µl of extract (containing 500 ug of total protein) with 100 ng of MBP-MYB4 protein purified from *E. coli* at 22 °C and sampled at different time intervals. Finally, western blotting was performed to detect MYB4 protein abundance.

### Quantification and statistical analysis

Statistical analyses and related parameters are described in the figure legends. Data processing and statistical tests were performed using GraphPad Prism 10. Statistical differences between two datasets were evaluated using two-tailed Student’s *t*-tests. When more than two groups were compared, one-way or two-way ANOVA followed by suitable post hoc multiple-comparison tests was applied. A threshold of *P* < 0.05 was considered statistically significant. Different letters denote significant differences between groups. Values are represented as means ± SD.

## Supplementary information

Appendix

Peer Review File

Source data Fig. 1

Source data Fig. 2

Source data Fig. 3

Source data Fig. 4

Source data Fig. 5

Source data Fig. 6

Source data Fig. 7

Source data Fig. 8

Source data Fig. 9

Expanded View Figures

## Data Availability

This study did not generate any new datasets requiring deposition in a public repository. The source data of this paper are collected in the following database record: biostudies:S-SCDT-10_1038-S44319-026-00906-1.
