# Supplementary material for: MPK3-mediated phosphorylation inactivates MYB4 to promote FIT-dependent iron acquisition
Source: EMBO Rep. 2026 Aug 25;27(19):5632–58. doi: 10.1038/s44319-026-00906-1 (PMC13649788; doi:10.1038/s44319-026-00906-1)
Supplement: Supplementary file 1 [file 44319_2026_906_MOESM12_ESM.pdf]

Expanded View Figures

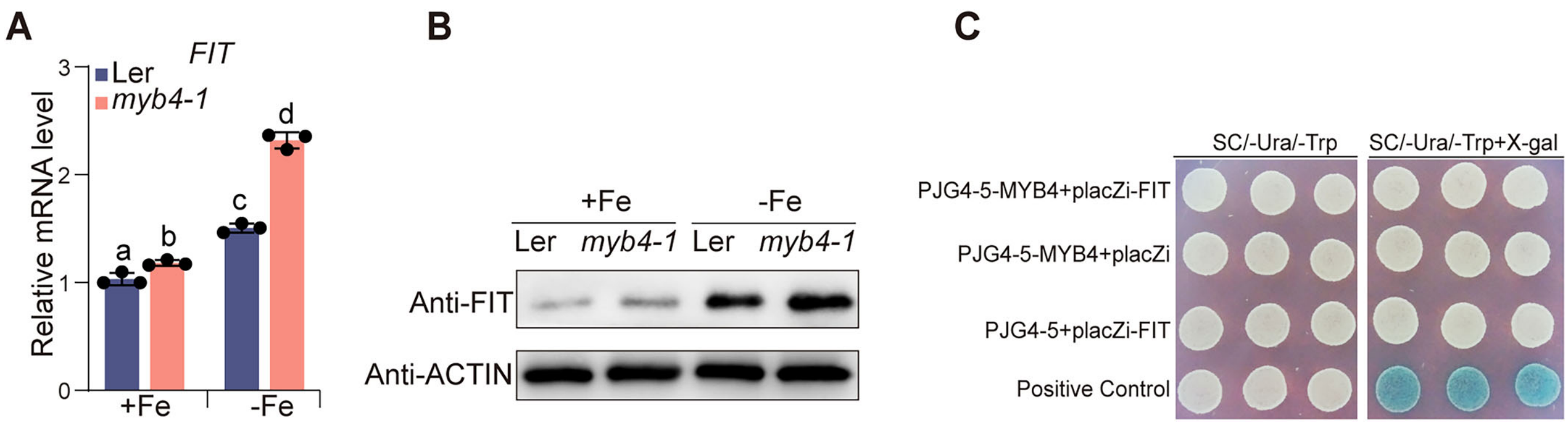

Figure EV1. Quantitative analysis of *FIT* transcript and protein levels in WT and *myb4-1* plants under Fe-deficiency stress.

(**A**) Expression levels of *FIT* in WT and *myb4-1* plants were detected by RT-qPCR under Fe deficiency conditions. Arabidopsis seedlings were grown under both normal and Fe-deficient conditions, and roots were harvested for RNA extraction followed by RT-qPCR analysis. *ACTIN8* was used as an internal control. Values shown are means ± SD ($n = 3$ biological replicates). Significance was determined using two-way ANOVA followed by Tukey's. *P* values = 0.0352 (+Fe Ler vs +Fe *myb4-1*), <0.0001 (+Fe Ler vs -Fe Ler), <0.0001 (+Fe Ler vs -Fe *myb4-1*), 0.003 (+Fe *myb4-1* vs -Fe Ler), <0.0001 (+Fe *myb4-1* vs-Fe *myb4-1*), <0.0001 (-Fe Ler vs -Fe *myb4-1*). (**B**) FIT protein levels in WT and *myb4-1* mutant plants were analyzed by Western blotting under Fe-deficiency conditions. Protein extracts from treated seedlings were analyzed by 10% SDS-PAGE and Western blotting assay using an anti-FIT antibody (upper panel). Total protein stained with Coomassie (lower panel), bright blue was used as the control. (**C**) MYB4 cannot interact with the *FIT* promoter in yeast. Y1H assays were performed on synthetic dropout (SD) medium lacking tryptophan and uracil (SD/-Trp/-Ura) to select yeast transformants carrying both bait and prey plasmids. Protein-DNA interactions were further assessed on SD/-Trp/-Ura medium supplemented with X-Gal (SD/-Trp/-Ura+X-Gal). The empty PJG4-5 and placZi prey vector is used as a negative control.

 © The Author(s)

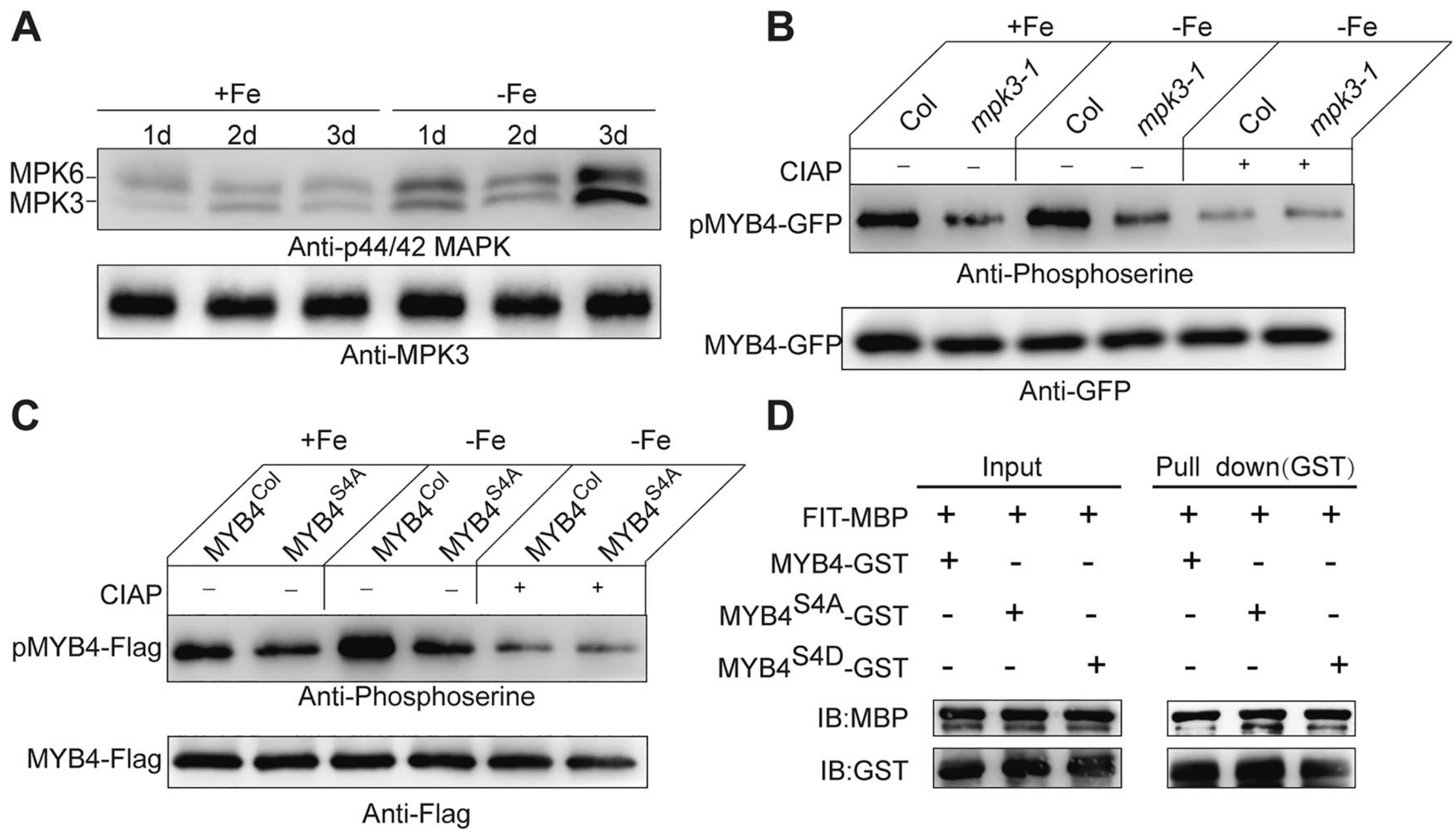

Figure EV2. Under Fe-deficiency stress, MPK3 regulates MYB4 accumulation by phosphorylating MYB4 at Ser4.

(**A**) Fe deficiency enhances MPK3 kinase activity. Proteins were extracted from wild-type Arabidopsis seedlings grown under Fe-sufficient or Fe-deficient conditions and subjected to gel-based kinase assays. MPK3 kinase activity was elevated under Fe-deficient conditions. MAPK activation was verified by immunoblotting with an anti-phospho-p44/42 MAPK antibody. Equivalent amounts of MPK3 protein were used for kinase activity measurements to evaluate changes in intrinsic kinase activity. (**B**) The effect of *mpk3-1* mutation on the phosphorylation of MYB4. The seedlings of the Col plants carrying the *35S:MYB4-GFP* gene and the *mpk3-1* plants were all cultivated on a medium with sufficient Fe for 10 days. Then, they were transferred to either Fe-sufficient or Fe-deficient medium for 3 days and immediately harvested. The seedlings were collected and processed for immunoprecipitation of MYB4-GFP, and subsequently, phosphorylation detection was performed using anti-Phosphoserine. (**C**) Effect of Ser4A mutation on MYB4 phosphorylation. The seedlings in the *myb4-2* background that carried the *ProMYB4:MYB4-Flag* ($MYB4^{Col}$) or *ProMYB4:MYB4*^{S4A}-*Flag* ($MYB4^{S4A}$) genes were all cultivated on a medium containing sufficient Fe for 10 days. Then, they were transferred to either Fe-sufficient or Fe-deficient medium for 3 days and immediately harvested. The seedlings were collected and processed for immunoprecipitation of MYB4-Flag, and subsequently, phosphorylation detection was performed using anti-Phosphoserine. (**D**) The effects of the Ser4A and Ser4D mutations on the interaction between MYB4 and FIT were examined using pull-down assays. For the pull-down assays, recombinant FIT-MBP protein was incubated with MYB4-GST, $MYB4^{S4A}$-GST, or $MYB4^{S4D}$-GST proteins at 4 °C for 5 h.
