# Supplementary material for: MPK3-mediated phosphorylation inactivates MYB4 to promote FIT-dependent iron acquisition
Source: EMBO Rep. 2026 Aug 25;27(19):5632–58. doi: 10.1038/s44319-026-00906-1 (PMC13649788; doi:10.1038/s44319-026-00906-1)
Supplement: Supplementary file 2 [file 44319_2026_906_MOESM1_ESM.pdf]

Appendix for

MPK3-mediated phosphorylation inactivates MYB4 to promote FIT-dependent iron acquisition

Table of contents

Appendix Figure S1. Phenotypes of *myb4-1* mutants under Mn-, Cu-, or Zn-deficient conditions. 2

Appendix Figure S2. Investigate the degradation pathway of MYB4 and its potential interactions with bHLH38, bHLH39, bHLH100, and bHLH101 proteins, as well as its binding activity toward the promoters of IRT1 and FRO2........ 3

Appendix Figure S3. The effect of MPK3-mediated phosphorylation on MYB4........... 4

Appendix Table S1. Primer sequences used in the manuscript........ 6

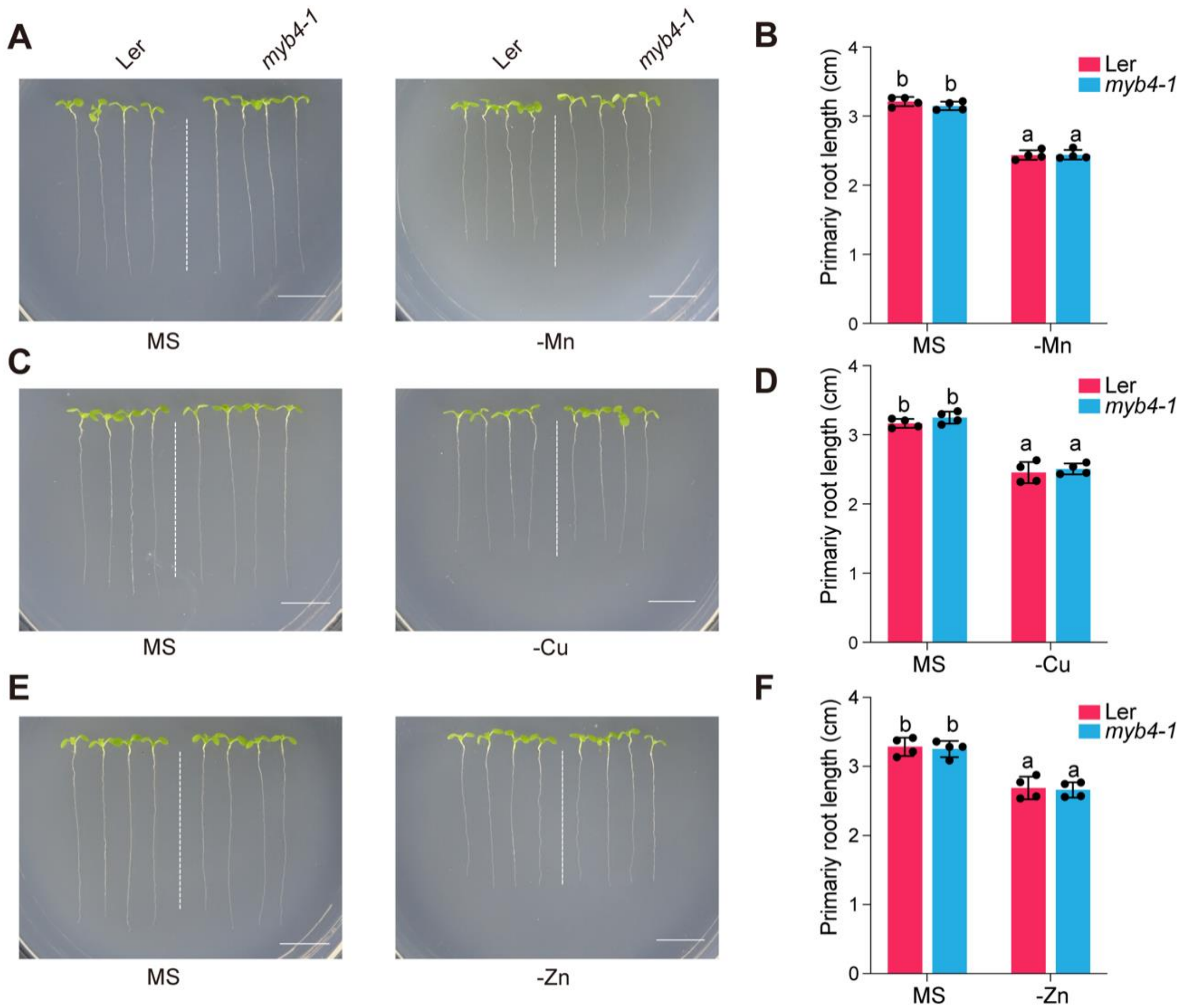

Appendix Figure S1. Phenotypes of *myb4-1* mutants under Mn-, Cu-, or Zn-deficient conditions.

(**A**) Analysis of Mn-deficiency tolerance in Ler and *myb4-1* seedlings. Scale bar: 1 cm. (**B**) Root length of plants described in A. (**C**) Analysis of Cu-deficiency tolerance in Ler and *myb4-1* seedlings. Scale bar: 1 cm. (**D**) Root length of plants described in C. (**E**) Analysis of Zn-deficiency tolerance in Ler and *myb4-1* seedlings. Bar= 1 cm. (**F**) Root length of plants described in E. All data were subjected to at least three independent replicate experiment. All data are presented as means±SD. Statistical significance was determined by Tukey's test, with different lowercase letters indicating significant differences ($P < 0.05$).

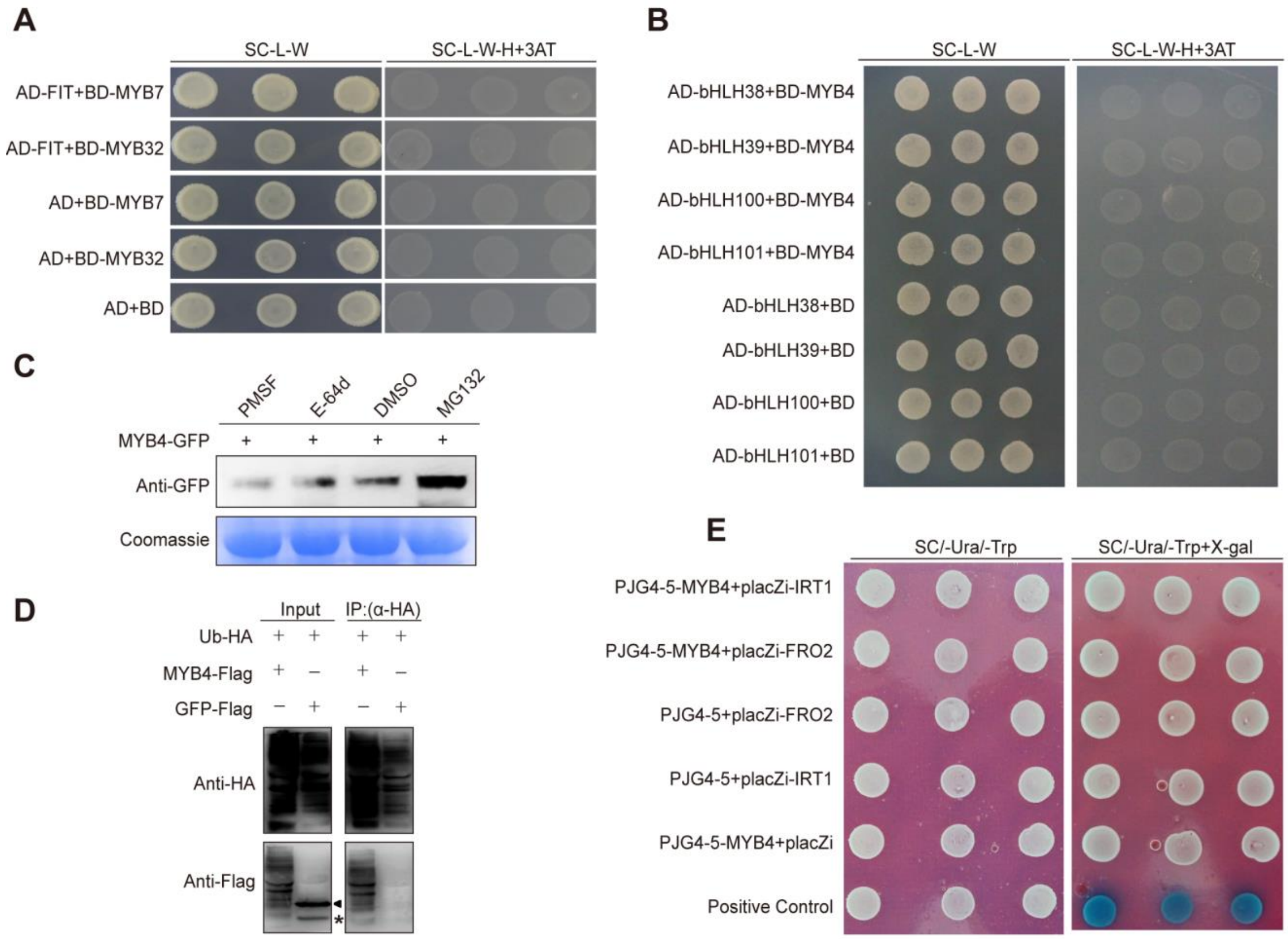

Appendix Figure S2. Investigate the degradation pathway of MYB4 and its potential interactions with bHLH38, bHLH39, bHLH100, and bHLH101 proteins, as well as its binding activity toward the promoters of *IRT1* and *FRO2*.

(**A**) Y2H assay to determine the interaction between MYB4 and bHLH38/39/100/101. (**B**) The interaction of FIT with MYB7/32, a homologue of MYB4, was detected by Y2H assay. (**C**) Effect of different protein degradation inhibitors on the degradation of MYB4-GFP protein. Proteins were extracted from treated *N. benthamiana* leaves, analyzed by 9% SDS-PAGE and analyzed by Western blotting using anti-GFP antibody (upper panel). Coomassie (lower panel) Brilliant Blue staining of total proteins was used as an internal control. (**D**) Ubiquitination of MYB4 in vivo was detected by Co-IP assay. *35S:Ub-HA* and *35S:GFP-FLAG* or *35S:MYB4-FLAG* were transiently co-expressed in *N. benthamiana* leaves. Total protein was extracted and then incubated with anti-HA agarose. Immunoprecipitated proteins were analyzed by Western blotting using anti-FLAG and anti-HA antibodies. (**E**)

The results of the Y1H assay indicated that MYB4 was not able to bind directly to the promoters of *IRT1* and *FRO2*.

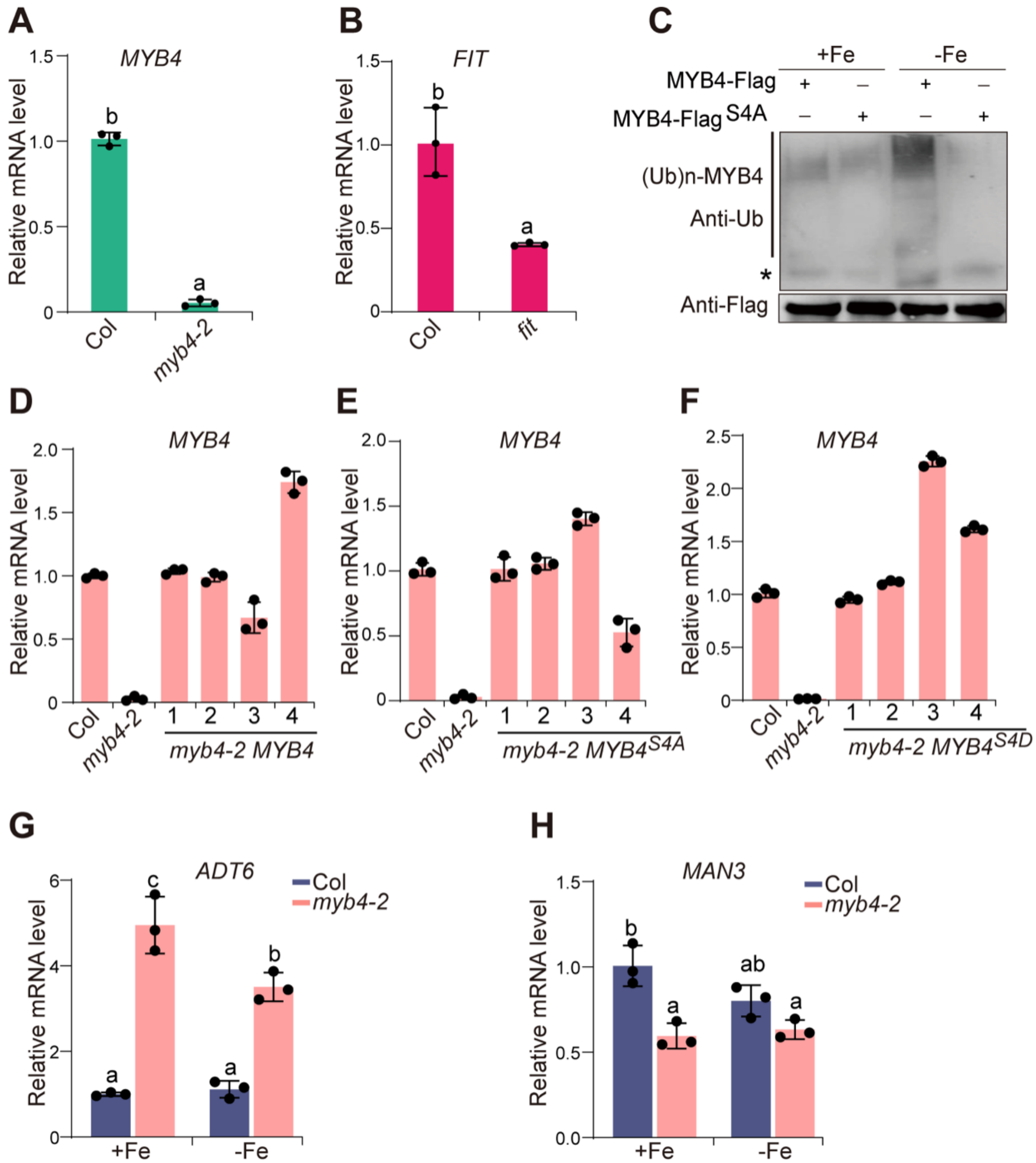

Appendix Figure S3. The effect of MPK3-mediated phosphorylation on MYB4.

(**A, B**) RT-qPCR identification of *FIT* and *MYB4* gene expression in *fit* and *myb4-2* mutant plants. Col, *myb4-2*, and *fit* mutants were grown under normal soil culture conditions for 4 weeks. *ACTIN8* was used as an internal control. Data are presented as means±SD of three independent biological replicates and significant differences (Student's-test, $P < 0.05$) are indicated by different lowercase letters. (**C**) Polyubiquitination of MYB4 and MYB4^{S4A} in vivo under normal and Fe deficit stress. *ProMYB4:MYB4-Flag/myb4-2* and

ProMYB4:MYB4^{S4A}-Flag/myb4-2 transgenic plants were grown vertically on normal medium for 10 days and then transferred to normal and Fe-deficient medium for 3 days for germination. Total protein extracts were immunoprecipitated with anti-Flag agarose beads, and then western immunoblotting analysis of the precipitated proteins was performed with anti-ubiquitin antibodies (upper panel). Immunoblot analysis of total protein extracts was performed using anti-Flag antibody as loading control (lower panel). Asterisks denote non-specific bands from anti-Flag agarose beads. (**D-F**) RT-qPCR identification of *MYB4* gene expression in Col, *myb4-2*, *ProMYB4:MYB4-Flag/myb4-2*, *ProMYB4:MYB4-Flag^{S4A}/myb4-2* and *ProMYB4:MYB4-Flag^{S4D}/myb4-2* transgenic plants. Transgenic materials expressing *MYB4-Flag/myb4-2,* *MYB4-Flag^{S4A}/myb4-2* and *MYB4-Flag^{S4D}/myb4-2* driven by the MYB4 native promoter, were grown under normal soil culture conditions for 4 weeks. *ACTIN8* was used as an internal control. Gene expression levels in WT plants were normalized to 1.0. All data represent the mean of three technical replicates ± SD and are representative of three independent biological experiments with consistent results. (**G, H**) Expression levels of *ADT6* (**G**) and *MAN3* (**H**) in Col and *myb4-2* plants were detected by RT-qPCR under both normal and Fe-deficient conditions. Arabidopsis seedlings were grown under both normal and Fe-deficiency conditions, and roots were harvested for RNA extraction followed by RT-qPCR analysis. *ACTIN8* was used as an internal control. Values shown are means ± SD (n = 3 biological replicates). Significance was determined using two-way ANOVA followed by Tukey's. (**G**) *P* values <0.0001 (+Fe Col vs +Fe *myb4-2*), 0.9828 (+Fe Col vs -Fe Col), 0.0002 (+Fe Col vs -Fe *myb4-2*), <0.0001 (+Fe *myb4-2* vs -Fe Col), 0.0077 (+Fe *myb4-2* vs-Fe *myb4-2*), 0.0003 (-Fe Col vs -Fe *myb4-2)*, (The following is the same order). (**H**) *P* values=0.0021, 0.0834, 0.0038, 0.0840, 0.9545, 0.1712.

Appendix Table S1. Primer sequences used in the manuscript.

Primers used for mutant isolation	
myb4-2-LP	ATGGGAAGGTCACCGTGCTG
myb4-2-RP	TTTCATCTCCAAGCTTCGAA
myb4-2-BPDs	ACCCGACCGGATCGTATCGGT
fit-LP	AGCCGATTGCAAATTAATTCC
fit-RP	GCTTATCCTTCATACGCCCTC
mpk3-1-LP	ATTTTTGTCAACAATGGCCTG
mpk3-1-RP	TCTGCCTTTTCACGGAATATG
mpk3-2-LP	CATGAATAAAAGAACAGGCAAAG
mpk3-2-RP	TTGGTGTTTTTGTTGTCATGG
SALK-BP	ATTTTGCCGATTTCGGAAC
Primers used for Y2H	
AD-FIT-Y2HF	**GGAATTCCAT**ATGATGGAAGGAAGAGTCAACGCTCTGTCAA
AD-FIT-Y2HR	**CGGGATCC**TCAAGTAAATGACTTGATGAATTCAAAA
AD-FITN-Y2HF	**gtaccagattacgctcatatg**ATGGAAGGAAGAGTCAACGCTC
AD-FITN-Y2HR	**cagctcgagctcgatggatcc**GGTCCCATCATTATTCGTAGTAGTGG
AD-FITM-Y2HF	**gtaccagattacgctcatatg**CGTAAGACGAAGACTGATCGGTC
AD-FITM-Y2HR	**cagctcgagctcgatggatcc**CTTTTGAGCATCAGGAGCATGTT
AD-FITC-Y2HF	**gtaccagattacgctcatatg**ACTCAACCTTTTCGCGGTATCA
AD-FITC-Y2HR	**cagctcgagctcgatggatcc**TCAAGTAAATGACTTGATGAATTCAAAA
AD-MYB4-Y2HF	**GGAATTC**ATGGGAAGGTCACCGTGCT

AD-MYB4-Y2HR	**CGGGATCC**TTATTTCATCTCCAAGCTTCGAAAG
BD-MYB4-Y2HF	**GGAATTC**ATGGGAAGGTCACCGTGCT
BD-MYB4-Y2HR	**CGGGATCC**TTATTTCATCTCCAAGCTTCGAAAG
BD-MPK3-Y2HF	**atggccatggaggccgaattc**ATGAACACCGGCGGTGGC
BD-MPK3-Y2HR	**ccgctgcaggtcgacggatcc**CTAACCGTATGTTGGATTGAGTGC
AD-bHLH38-Y2HF	**GGAATTC**ATGTGTGCATTAGTCCCTTCATTTT
AD-bHLH38-Y2HR	**CGGGATCC**CTAGTTAAACGAGTTTTCACATTTCTCG
BD-bHLH38-Y2HF	**atggccatggaggccgaattc**ATGTGTGCATTAGTCCCTTCA
BD-bHLH38-Y2HR	**ccgctgcaggtcgacggatcc**CTAGTTAAACGAGTTTTCACA
AD-bHLH39-Y2HF	**GGAATTC**ATGTGTGCATTAGTACCTCCATTGTT
AD-bHLH39-Y2HR	**CGGGATCC**TCATATATATGAGTTTCCACATTCCTCA
BD-bHLH39-Y2HF	**atggccatggaggccgaattc**ATGTGTGCATTAGTACCTCCAT
BD-bHLH39-Y2HR	**ccgctgcaggtcgacggatcc**TCATATATATGAGTTTCCACATT
AD-bHLH100-Y2HF	**GGAATTCCAT**ATGATGTGTGCACTTGTCCCTCCA
AD-bHLH100-Y2HR	**CGGGATCC**TCATGTAAACGAGTGTCCACATTTC

AD-bHLH101-Y2HF	**GGAATTC**ATGTGTACCTTAACGCCAATGTTT
AD-bHLH101-Y2HR	**CGGGATCC**TTATGATTGGCGTAATCCCAAGA
BD-MYB7-Y2HF	**GGAATTC**ATGGGAAGATCTCCTTGCTG
BD-MYB7-Y2HR	**CGGGATCC**TTATTTCATTTCCAAGCTTCGAAAAT
BD-MYB32-Y2HF	**GGAATTCCATATG**ATGGGAAGGTCTCCTTGCTG
BD-MYB32-Y2HR	**CGGGATCC**TCATTTCATTTCCAAAGTGCTAAAATC
Primers used for Y3H	
bHLH38-MCSI-F	**tgactgtatcgccggaattc**ATGTGTGCATTAGTCCCTTCATTTT
bHLH38-MCSI-R	**ggctgcaggtcgacggatcc**CTAGTTAAACGAGTTTTCACATTTCTCG
bHLH39-MCSI-F	**tgactgtatcgccggaattc**ATGTGTGCATTAGTACCTCCATTGTT
bHLH39-MCSI-R	**tggctgcaggtcgacggatcc**TCATATATATGAGTTTCCACATTCCTCA
MYB4-MCSII-F	**aagaagagaaaggtggcggccgc**ATGGGAAGGTCACCGTGCT
MYB4-MCSII-R	**gggagatcagcccgaagatct**CGGGATCCTTATTTCATCTCCAAGCT
MBP-MCSII-F	**aagaagagaaaggtggcggccgc**ATGAAAATCGAAGAAGGTAAACTG
MBP-MCSII-R	**gggagatcagcccgaagatct**CTTAGTCTGCGCGTCTTTCAG
Primers used for Co-IP assay	
HA-MYB4-F	**GGGGTACC**ATGGGAAGGTCACCGTGCT
HA-MYB4-R	**GAAGGCCT**TTTCATCTCCAAGCTTCGAAAG

Flag-FIT-F	**CG<u>GGATCC</u>**ATGGAAGGAAGAGTCAACGCT
Flag-FIT-R	**ACGC<u>GTCGAC</u>**AGTAAATGACTTGATGAATTCAAAAC
Flag-MPK3-F	**GG<u>GGTACC</u>**ATGAACACCGGCGGTGGC
Flag-MPK3-R	**CCG<u>CTCGAG</u>**ACCGTATGTTGGATTGAGTGCTA
Primers used for BIFC assay	
MYB4-cYFPF	**CG<u>GGATCC</u>**ATGGGAAGGTCACCGTGCT
MYB4-cYFPR	**CCG<u>CTCGAG</u>**TTTCATCTCCAAGCTTCGAAAG
FIT-nYFPF	**CG<u>GGATCC</u>**ATGGAAGGAAGAGTCAACGCTCTGTCAA
FIT-nYFPR	**CCG<u>CTCGAG</u>** AGTAAATGACTTGATGAATTCAAAACC
bHLH38-cYFPF	**CG<u>GGATCC</u>**ATGTGTGCATTAGTCCCTTCA
bHLH38-cYFPR	**CCG<u>CTCGAG</u>**GTTAAACGAGTTTTCACATTT
bHLH39-cYFPF	**CG<u>GGATCC</u>**ATGTGTGCATTAGTACCTCCA
bHLH39-cYFPR	**CCG<u>CTCGAG</u>**TATATATGAGTTTCCACATTC
MPK3-nYFPF	**CG<u>GGATCC</u>**ATGAACACCGGCGGTGGC
MPK3-nYFPR	**CCG<u>CTCGAG</u>**ACCGTATGTTGGATTGAGTGCTA
Primers used for LCI assay	
MYB4-nlciF	**acgggggacgagctcggtacc**ATGGGAAGGTCACCGTGCTG
MYB4-nlciR	**cgcgtacgagatctggtcgac**TTTCATCTCCAAGCTTCGAAAGC
$MYB4^{S4A}$-nlciF	**acgggggacgagctcggtacc**ATGGGAAGGGCTCCGTGCTG
$MYB4^{S4A}$-nlciR	**cgcgtacgagatctggtcgac**TTTCATCTCCAAGCTTCGAAAGC
$MYB4^{S4D}$-nlciF	**acgggggacgagctcggtacc**ATGGGAAGGGACCCGTGCTG
$MYB4^{S4D}$-nlciR	**cgcgtacgagatctggtcgac**TTTCATCTCCAAGCTTCGAAAGC
FIT-clciF	**tacgcgtcccggggcggtacc**ATGGAAGGAAGAGTCAACGCTC
FIT-clciR	**acgaaagctctgcaggtcgac**AGTAAATGACTTGATGAATTCAAAACC

Primers used for real-time quantitative PCR	
FIT-QRF	TCGGTCTAGGACTTTGATCTCTG
FIT-QRR	TCTTGAACATACAACACTGCATCT
FRO2-QRF	TTCACCGTTCATGGTCTTTGTT
FRO2QRR	GAGCTATCTCTCCGGCCAAATT
IRT1-QRF	CTCTTTGCTTCCATCAAATGTTC
IRT1-QRR	CCTAACGCTATTCCGAATGG
MYB4-QRF	CAAGGGCATGGAAAGTCAAC
MYB4-QRR	TTGCTGCTACCTCCGACTAC
ACTIN8-QRF	TCAGCACTTTCCAGCAGATG
ACTIN8-QRR	CTGTGGACAATGCCTGGAC
Primers used for yeast one-hybrid	
ProIRT1-placZiF	**GGGGTACC**CGAAGTTGACTAGTTAGGAGGCATG
ProIRT1-placZiR	**CCGCTCGAG**TTTTTTTTTTTTCTTTTTTCTTTTGG
ProFRO2-placZiF	**GGGGTACC**GACCTCTAGGAGGACTATTATTAA
ProFRO2-placZiR	**CCGCTCGAG**CCTCTCTTTCCTCTCAGGATTT
MYB4-PJG45F	**GGAATTC**ATGGGAAGGTCACCGTGCTGT
MYB4-PJG45R	**CCGCTCGAG**TTATTTCATCTCCAAGCTTCG
Primers used for overexpression	
MYB4-OEF	**gagaacacgggggacggtacc**ATGGGAAGGTCACCGTGCT
MYB4-OER	**ctaggtgcggccgcctcgag**TTATTTCATCTCCAAGCTTCGAAAG
FIT-OEF	**gagaacacgggggacggtacc**ATGGAAGGAAGAGTCAACGCTC
FIT-OER	**ctaggtgcggccgcctcgag**TCAAGTAAATGACTTGATGAATTCAAAAC
bHLH39-OEF	**gagaacacgggggacggtacc**ATGTGTGCATTAGTACCTCCATTGT

bHLH39-OER	**ctaggtgcggccgcctcgag**TCATATATATGAGTTTCCACATTCC
Primers used promoter cloning	
ProIRT1-GUSF	**acgcgttgggagctcggtacc**CGAAGTTGACTAGTTAGGAGGCATG
ProIRT1-GUSR	**cctaggtgcggccgcctcgag**TTTTTTTTTTTCTTTTTCTTTTGG
ProFRO2-GUSF	**GG<u>GGTACC</u>**GACCTCTAGGAGGACTATTATTAA
ProFRO2-GUSR	**CCG<u>CTCGAG</u>**CCTCTCTTTCCTCTCAGGATTT
Primers used for protein expression in EMSA assay	
MYB4-HisF	**GG<u>GGTACC</u>**ATGGGAAGGTCACCGTGCT
MYB4-HisR	**CG<u>GGATCC</u>**TTTCATCTCCAAGCTTCGAAAG
FIT-HisF	**aggcatatggagctcggtacc**ATGGAAGGAAGAGTCAACGCTC
FIT-HisR	**gacaagcttgaattcggatcc**AGTAAATGACTTGATGAATTCAAAACC
bHLH38-HisF	**aggcatatggagctcggtacc**ATGTGTGCATTAGTCCCTTCATTTT
bHLH38-HisR	**gacaagcttgaattcggatcc**GTTAAACGAGTTTTCACATTTCTCGT
bHLH39-HisF	**aggcatatggagctcggtacc**ATGTGTGCATTAGTACCTCCATTGTT
bHLH39-HisR	**gacaagcttgaattcggatcc**TATATATGAGTTTCCACATTCCTCATACA
Oligonucleotides used for EMSA assay	
EMSA-pIRT1-a-F	AGCACAAGCTGTCACGTGTGATGTTGTGCTC
EMSA-pIRT1-a-R	GAGCACAACATCACACGTGACAGCTTGTGCT
EMSA-pIRT1-b-F	GGAATCTACATTCATATGTAGTGTTTTTGG
EMSA-pIRT1-b-R	CCAAAAACACTACATATGAATGTAGATTCC
EMSA-pFRO2-a-F	ATGGGGAAGAGTCACATGCATGGAGCATGC

EMSA-pFRO2-a-R	GCATGCTCCATGCATGTGACTCTTCCCCAT
Primers used for ChIP assay	
IRT1-P1F	GTTTCACACTGGAAGCAAACGAG
IRT1-P1R	TGTGTGCTGGTTGTTTTCATGCC
IRT1-P2F	TAGACATCAAGCTATGAAAAGA
IRT1-P2R	CTATAGTTCTGTTAGTCAACTC
IRT1-P3F	ATAACATTGAAATATCTTAATA
IRT1-P3F	GGAAAGGATAATGACAAATA
FRO2-P1F	ACGTCATCTCGCCAAAACAACAA
FRO2-P1R	GGTACGATCGCTTTAGTGAGAA
FRO2-P2F	GTCCTTGGCCTCTTTCTCAAGTC
FRO2-P2R	AACGGTTAAGTATTCAGGGATGGG
FRO2-P3F	TGATGATGAGGAAGAAAGAAGA
FRO2-P3R	GATATGAGACCTCCCAAATTGGA
ACTIN2-F	GACGACTAACGTTGCCTGGA
ACTIN2-R	GACTGTGACCTGCTCGATCC
Primers used for yeast three-hybrid	
MYB4-MCSIF	**ttgactgtatcgccggaattc**ATGGGAAGGTCACCGTGCTGT
MYB4-MCSIR	**tggctgcaggtcgacggatcc**TTATTTCATCTCCAAGCTTCG
MBP-MCSIIF	**aagaagagaaaggtggcggccgc** ATGAAAATCGAAGAAGGTAAACTG
MBP-MCSIIR	**gggagatcagcccgaagatct** CTTAGTCTGCGCGTCTTTCAG
bHLH38-MCSIIF	**aagaagagaaaggtggcggccgc** ATGTGTGCATTAGTCCCTTCATTTT
bHLH38-MCSIIF	**gggagatcagcccgaagatct** CTAGTTAAACGAGTTTTCACATTTCTCG
bHLH39-MCSIIF	**aagaagagaaaggtggcggccgc** ATGTGTGCATTAGTACCTCCATTGTT

bHLH39-MCSIIF	**gggagatcagcccgaagatct** TCATATATATGAGTTTCCACATTCCTCA
Primers used for constructs in plant transformation	
MYB4-F	**tacgaattcgagctcggtacc**ATGGGAAGGTCACCGTGCTG
MYB4-R	**acgacggccagtgccaagctt**TTTCATCTCCAAGCTTCGAAAGC
MYB4^{S4A}-F	**tacgaattcgagctcggtacc**ATGGGAAGGGCTCCGTGCTG
MYB4^{S4A}-R	**acgacggccagtgccaagctt**TTTCATCTCCAAGCTTCGAAAGC
MYB4^{S4D}-F	**tacgaattcgagctcggtacc**ATGGGAAGGGACCCGTGCTG
MYB4^{S4D}-R	**acgacggccagtgccaagctt**TTTCATCTCCAAGCTTCGAAAGC
MYB4-GFPF	**gagaacacgggggacggtacc**ATGGGAAGGTCACCGTGCTG
MYB4-GFPR	**cctaggtgcggccgcctcgag**TTTCATCTCCAAGCTTCGAAAGC
Primers used for phosphorylation assays	
MYB4-GSTF	**gatctggttccgcgtggatcc**ATGGGAAGGTCACCGTGCT
MYB4-GSTR	**gtcacgatgcggccgctcgag**TTATTTCATCTCCAAGCTTCGAAAG
MYB4^{S4A}-GSTF	**gatctggttccgcgtggatcc**ATGGGAAGGGCTCCGTGC
MYB4^{S4A}-GSTR	**gtcacgatgcggccgctcgag**TTATTTCATCTCCAAGCTTCGAAA
MYB4^{T221A}-GSTF	**gatctggttccgcgtggatcc**ATGGGAAGGTCACCGTGCT
MYB4^{T221A}-GSTR	**gtcacgatgcggccgctcgag**TTATTTCATCTCCAAGCTTCGAAAG
MYB4^{2A}-GSTF	**gatctggttccgcgtggatcc**ATGGGAAGGGCTCCGTGC
MYB4^{2A}-GSTR	**gtcacgatgcggccgctcgag**TTATTTCATCTCCAAGCTTCGAAA
MPK3-MBPF	**aggatttcagaattcggatcc**ATGAACACCGGCGGTGGC
MPK3-MBPR	**caagcttgcctgcaggtcgac**CTAACCGTATGTTGGATTGAGTGC
MKK5^{DD}-MBPF	**aggatttcagaattcggatcc**ATGAAACCGATTCAATCTCCTTCT
MKK5^{DD}-MBPR	**caagcttgcctgcaggtcgac**CTAAGAGGCAGAAGGAAGAGGACG
