# Supplementary material for: MPK3-mediated phosphorylation inactivates MYB4 to promote FIT-dependent iron acquisition
Source: EMBO Rep. 2026 Aug 25;27(19):5632–58. doi: 10.1038/s44319-026-00906-1 (PMC13649788; doi:10.1038/s44319-026-00906-1)
Supplement: Supplementary file 3 [file 44319_2026_906_MOESM2_ESM.pdf]

EMBO reports

MPK3-mediated phosphorylation inactivates MYB4 to promote FIT-dependent iron acquisition

hui song, Guangna Chen, Jiena Xu, Jing Zhang, Shuangyao Li, Shuxun Xu, Yanjun Wu, Li Jiang, Xi Wu, and shuqing cao

Corresponding author(s): shuqing cao (shuqingcao@hfut.edu.cn) , Li Jiang (lijiang@hfut.edu.cn), Xi Wu (xiwu@hfut.edu.cn)

Review Timeline:

Submission Date:	10th Feb 26
Editorial Decision:	5th Mar 26
Revision Received:	25th Jun 26
Editorial Decision:	22nd Jul 26
Revision Received:	4th Aug 26
Accepted:	13th Aug 26

Editor: Esther Schnapp

Transaction Report:

(Note: With the exception of the correction of typographical or spelling errors that could be a source of ambiguity, letters and reports are not edited. Depending on transfer agreements, referee reports obtained elsewhere may or may not be included in this compilation. Referee reports are anonymous unless the Referee chooses to sign their reports.)

1st Editorial Decision 5th Mar 2026

Dear Prof. Cao,

Thank you for the transfer of your manuscript to EMBO reports and for your proposed revision plan.

I agree with your suggestions for how to revise your ms, and I would thus like to invite you to revise your manuscript with the understanding that the referee concerns must be fully addressed and their suggestions taken on board. Please address all referee concerns in a complete point-by-point response. Acceptance of the manuscript will depend on a positive outcome of a second round of review. It is EMBO reports policy to allow a single round of major revision only and acceptance or rejection of the manuscript will therefore depend on the completeness of your responses included in the next, final version of the manuscript.

We realize that it is difficult to revise to a specific deadline. In the interest of protecting the conceptual advance provided by the work, we recommend a revision within 3 months (5th Jun 2026). Please discuss the revision progress ahead of this time with the editor if you require more time to complete the revisions.

IMPORTANT NOTE: we perform an initial quality control of all revised manuscripts before re-review. Your manuscript will FAIL this control and the handling will be DELAYED if the following APPLIES:
1) A data availability section providing access to data deposited in public databases is missing. If you have not deposited any data, please add a sentence to the data availability section that explains that.
2) Your manuscript contains statistics and error bars based on n=2. Please use scatter blots in these cases. No statistics should be calculated if n=2.

When submitting your revised manuscript, please review the submission guidelines (https://link.springer.com/journal/44319/submission-guidelines#cms-Revised-submissions) and carefully review the instructions that follow below. Failure to include requested items will delay the evaluation of your revision.

1) a .docx formatted version of the manuscript text (including legends for main figures, EV figures and tables). Please make sure that the changes are highlighted to be clearly visible.

2) individual production quality figure files as .eps, .tif, .jpg (one file per figure). See hhttps://media.springernature.com/original/springer-cms/rest/v1/content/27825798/data/v1 for more info on how to prepare your figures.

3) We replaced Supplementary Information with Expanded View (EV) Figures and Tables that are collapsible/expandable online. A maximum of 5 EV Figures can be typeset. EV Figures should be cited as 'Figure EV1, Figure EV2" etc... in the text and their respective legends should be included in the main text after the legends of regular figures.

- For the figures that you do NOT wish to display as Expanded View figures, they should be bundled together with their legends in a single PDF file called *Appendix*, which should start with a short Table of Content. Appendix figures should be referred to in the main text as: "Appendix Figure S1, Appendix Figure S2" etc. See 'Expanded View' section of the submission guidelines.

- Additional Tables/Datasets should be labeled and referred to as Table EV1, Dataset EV1, etc. Legends have to be provided in a separate tab in case of .xls files. Alternatively, the legend can be supplied as a separate text file (README) and zipped together with the Table/Dataset file.

4) a .docx formatted letter INCLUDING the reviewers' reports and your detailed point-by-point responses to their comments. As part of the EMBO Press transparent editorial process, the point-by-point response is part of the Review Process File (RPF), which will be published alongside your paper.

5) a complete author checklist, which you can download from our author guidelines. Please insert information in the checklist that is also reflected in the manuscript. The completed author checklist will also be part of the RPF.

6) Please note that all corresponding authors are required to supply an ORCID ID for their name upon submission of a revised manuscript (<https://orcid.org/>), which can be linked to your profile in the manuscript submission system.

7) Before submitting your revision, primary datasets produced in this study need to be deposited in an appropriate public database. Please remember to provide a reviewer password if the datasets are not yet public. The accession numbers and database should be listed in a formal "Data Availability" section placed after Methods. Please note that the Data Availability Section is restricted to new primary data that are part of this study. * Note - All links should resolve to a page where the data can be accessed. *

If your study has not produced novel datasets, please mention this fact in the Data Availability Section.

8) At EMBO Press we ask authors to provide source data for the main manuscript figures. You will receive a separate email with instructions for providing source data with your revised manuscript, including how to upload and organize the files.

9) Our journal also encourages inclusion of *data citations in the reference list* to directly cite datasets that were re-used and obtained from public databases. Data citations in the article text are distinct from normal bibliographical citations and should directly link to the database records from which the data can be accessed. In the main text, data citations are formatted as follows: "Data ref: Smith et al, 2001" or "Data ref: NCBI Sequence Read Archive PRJNA342805, 2017". In the Reference list, data citations must be labeled with "[DATASET]". A data reference must provide the database name, accession number/identifiers and a resolvable link to the landing page from which the data can be accessed at the end of the reference.

10) Regarding data quantification, the following points must be specified in each figure legend:

- the name of the statistical test used to generate error bars and P values,

- the number (n) of independent experiments (please specify technical or biological replicates) underlying each data point,

- the nature of the bars and error bars (s.d., s.e.m.),

- If the data are obtained from n Program fragment delivered error ``Can't locate object method "less" via package "than" (perhaps you forgot to load "than"?) at //ejpvfs23/sites23b/embor_www/letters/embor_decision_revise_and_review.txt line 54.' 2, use scatter blots showing the individual data points.

Discussion of statistical methodology can be reported in the materials and methods section, but figure legends should contain a basic description of n, P and the test applied.

- Please also include scale bars in all microscopy images.

11) The journal requires a statement specifying whether or not authors have competing interests (defined as all potential or actual interests that could be perceived to influence the presentation or interpretation of an article). In case of competing interests, this must be specified in your disclosure statement.

12) All Materials and Methods need to be described in the main text using our 'Structured Methods' format, which is required for all research articles. According to this format, the Methods section includes a separate Reagents and Tools Table file (listing key reagents, experimental models, software and relevant equipment and including their sources and relevant identifiers) and a Methods and Protocols section describing the methods using a step-by-step protocol format. The aim is to facilitate adoption of the methodologies across labs. More information on how to adhere to this format as well as a downloadable template (.docx) for the Reagents and Tools Table can be found in our author guidelines.

We would also welcome the submission of cover suggestions, or motifs to be used by our Graphics Illustrator in designing a cover.

As part of the EMBO publication's Transparent Editorial Process, EMBO reports publishes online a Review Process File (RPF) to accompany accepted manuscripts. This File will be published in conjunction with your paper and will include the referee reports, your point-by-point response and all pertinent correspondence relating to the manuscript.

You are able to opt out of this by letting the editorial office know (emboreports@embo.org). If you do opt out, the Review Process File link will point to the following statement: "No Review Process File is available with this article, as the authors have chosen not to make the review process public in this case."

I look forward to seeing a revised form of your manuscript when it is ready. Please use this link to submit your revision:
https://embor.msubmit.net/cgi-bin/main.plex

Best regards,
Esther

Esther Schnapp, PhD
Senior Editor
EMBO reports

Dear Editor:

Thank you very much for your review of our manuscript. We decided to resubmit a revised version of our paper (EMBOR-2026-63836-T). In order to improve our research, we have revised the paper and more supplemental experiments were carried out according to suggestions from editors and reviewers. We have revised the manuscript in response to the reviewer's comments, and we have marked the revised position in red font. A point-by-point reply to the comments of reviewers are as follows:

Response to the Editor's Comments:

I agree with your suggestions for how to revise your ms, and I would thus like to invite you to revise your manuscript with the understanding that the referee concerns must be fully addressed and their suggestions taken on board. Please address all referee concerns in a complete point-by-point response. Acceptance of the manuscript will depend on a positive outcome of a second round of review. It is EMBO reports policy to allow a single round of major revision only and acceptance or rejection of the manuscript will therefore depend on the completeness of your responses included in the next, final version of the manuscript.

Response: We sincerely appreciate your positive evaluation of our manuscript and the opportunity to revise it. We fully recognize the importance of thoroughly addressing all comments and suggestions provided by the reviewers. We have carefully revised the manuscript and provided detailed responses to each of the reviewers' comments. We understand that the final decision will depend on the completeness and quality of this revision, and we have made every effort to ensure that all issues are comprehensively resolved. We believe these revisions will significantly improve the quality of our manuscript.

Responses to comments of the Reviewer 1:

The transcription factor FIT plays a central role in regulating plant responses to iron (Fe) deficiency. In this manuscript, the authors identify the R2R3-MYB transcription factor MYB4 as a negative regulator of FIT activity. They show that MYB4 competes

with bHLH38/39 for interaction with FIT, thereby preventing the formation of transcriptionally active heterodimers and repressing the expression of Fe uptake genes IRT1 and FRO2. Furthermore, the authors report that the kinase MPK3 phosphorylates MYB4 at Ser4, promoting MYB4 degradation, which in turn facilitates FIT-bHLH38/39 heterodimer formation and enhances plant tolerance to Fe deficiency. This study proposes a previously uncharacterized MPK3-MYB4-FIT signaling module that positively regulates plant adaptation to Fe deficiency. However, several issues should be addressed to fully substantiate the conclusions and improve the manuscript.

Response: We appreciate the reviewers' positive evaluation of our study and their recognition of the novelty and significance of the proposed MPK3-MYB4-FIT signaling module in regulating plant iron deficiency responses. We fully agree with the reviewers' comments that certain aspects of the manuscript still require further clarification, and additional evidence is needed to more thoroughly support our conclusions. In response, we have carefully revised the manuscript and conducted additional analyses to address all raised questions. Details are provided below.

Specific comments:

1. Lines 192-193: Although MYB4 suppresses IRT1 and FRO2 expression, this result alone does not demonstrate that the inhibition occurs through a FIT-dependent pathway.

Response: We thank the reviewer for this important and constructive comment. Thank you for the reviewer's comments; we have revised the expression here. Please refer to the revised manuscript for details (Lines 195-206, Lines 316-330).

2. It remains unclear whether MYB4 regulates FIT at the transcriptional level or affects FIT protein accumulation. This distinction should be clarified.

Response: We thank the reviewer for this important suggestion. We observed that FIT transcript and protein levels are moderately elevated in the *myb4* mutant compared to wild type under Fe-deficient conditions (Fig. 1A, B). However, our data do not support a direct transcriptional or post-translational regulation of FIT by MYB4. First,

MYB4 does not bind to the FIT promoter (Fig. 1C), and no evidence suggests that MYB4 directly regulates FIT transcription. Second, our biochemical and genetic analyses consistently demonstrate that MYB4 physically interacts with FIT and interferes with the formation of transcriptionally active FIT-bHLH38/39 complexes. We therefore propose that the increased FIT transcript and protein levels observed in the *myb4* mutant likely represent an indirect consequence of altered Fe homeostasis and enhanced Fe-deficiency signaling, rather than a direct regulatory effect of MYB4 on FIT expression or stability. To clarify this point, we have revised the manuscript to emphasize that MYB4 primarily regulates FIT activity through competitive protein-protein interaction. Please refer to the revised manuscript for details (Lines 195-206, Figure EV1A-C).

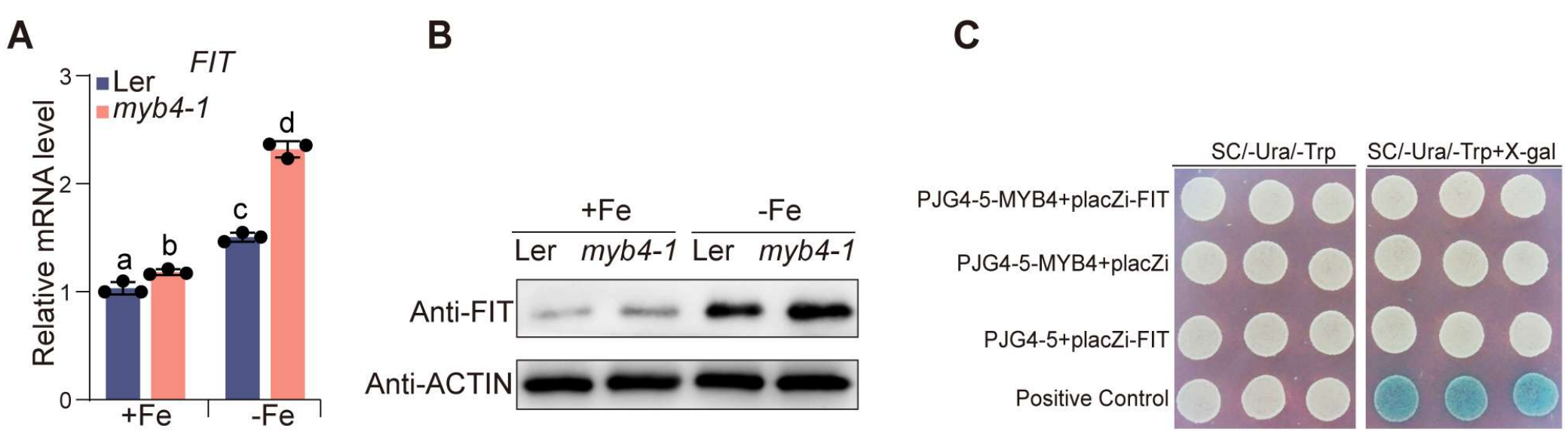

Figure 1. Quantitative analysis of *FIT* transcript and protein levels in WT and *myb4-1* plants under Fe-deficiency stress. (A) Expression levels of *FIT* in WT and *myb4-1* plants were detected by RT-qPCR under Fe deficiency conditions. Arabidopsis seedlings were grown under both normal and Fe-deficiency conditions, and roots were harvested for RNA extraction followed by RT-qPCR analysis. *ACTIN8* was used as an internal control. All data were subjected to at least three independent replicate experiments, each with three biological replicates. All data are presented as means ± SD. Statistical significance was determined by Tukey's test, with different lowercase letters indicating significant differences ($P < 0.05$). (B) FIT protein levels in WT and *myb4-1* mutant plant were analyzed by Western blotting under Fe-deficiency conditions. Protein extracts from treated seedlings were analyzed by 10% SDS-PAGE and Western blot assay using an anti-FIT antibody (upper panel). Total protein stained with Coomassie (lower panel), bright blue was used as the control. (C) MYB4 cannot

interact with the *FIT* promoter in yeast. Y1H assays were performed on synthetic dropout (SD) medium lacking tryptophan and uracil (SD/−Trp−Ura) to select yeast transformants carrying both bait and prey plasmids. Protein-DNA interactions were further assessed on SD/−Trp−Ura medium supplemented with X-Gal (SD/−Trp−Ura−X-Gal). The empty PJG4-5 and placZi prey vector is used as a negative control.

3. To strengthen the conclusion that MPK3 phosphorylates MYB4 at Ser4, the authors should examine whether MYB4 phosphorylation is reduced in the mpk3 mutant. In addition, they should test whether the S4A mutation diminishes MYB4 phosphorylation in planta.

Response: We thank the reviewer for this valuable suggestion. We agree that directly examining MYB4 phosphorylation in the *mpk3* mutant and assessing the effect of the S4A mutation in planta are important to strengthen our conclusion. To test whether MYB4 phosphorylation depends on MPK3 in planta, we analyzed the phosphorylation status of MYB4 in the *mpk3* mutant background. The results show a clear reduction in the phosphorylation signal of MYB4 in *mpk3* compared with Col plants under Fe-deficient conditions (Figure 2A). This trend indicates that MPK3 is a major kinase responsible for MYB4 phosphorylation in vivo.

Furthermore, to assess the effect of the S4A mutation, we examined the phosphorylation level of $MYB4^{S4A}$ in transgenic plants. It was found that the phosphorylation signal of $MYB4^{S4A}$ was significantly lower than that of the wild-type MYB4 (Figure 2B). This result further confirmed that Ser4 is the main phosphorylation site of MYB4 in plants. Please refer to the revised manuscript for details (Lines 363-371, Figure EV5).

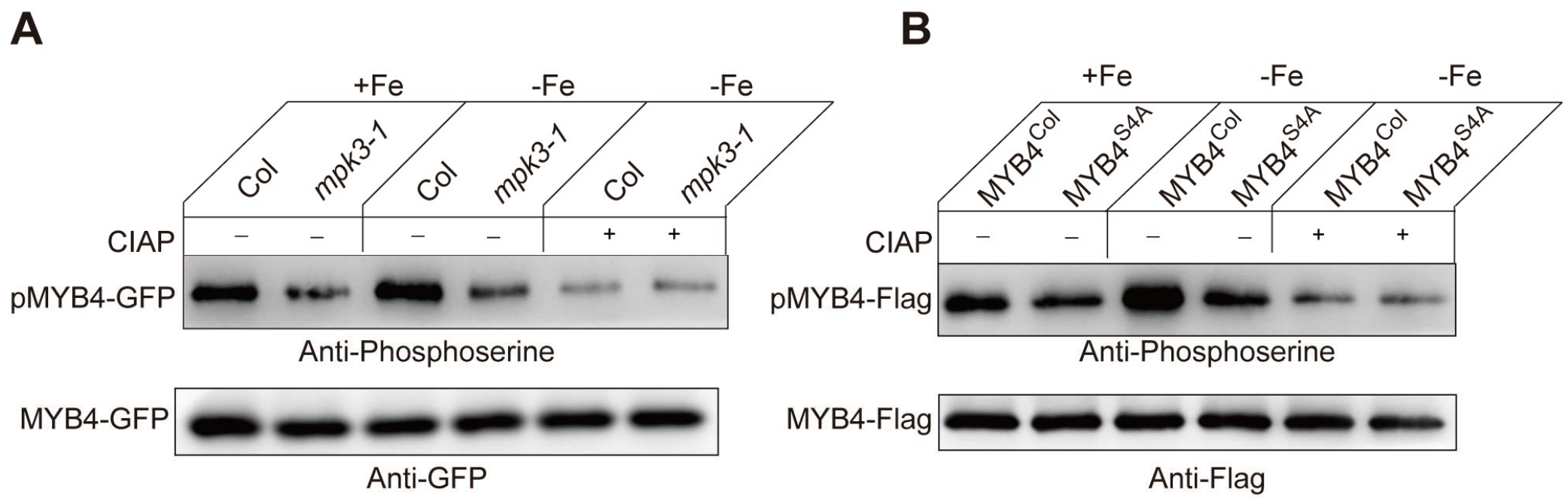

Figure 2. Under Fe-deficiency stress, MPK3 regulates MYB4 accumulation through phosphorylating MYB4 at Ser4. (A) The effect of *mpk3-1* mutation on the phosphorylation of MYB4. The seedlings of the Col plants carrying the MYB4-GFP gene and the *mpk3-1* plants were all cultivated on a medium with sufficient Fe for 10 days. Then, they were transferred to either Fe-sufficient or Fe-deficient medium for 3 days and immediately harvested. The seedlings were collected and processed for immunoprecipitation of MYB4-GFP, and subsequently, phosphorylation detection was performed using anti-Phosphoserine. (B) Effect of Ser4A mutation on MYB4 phosphorylation. The seedlings in the *myb4-2* background that carried the MYB4-Flag (MYB4^{Col}) or MYB4^{S4A}-Flag (MYB4^{S4A}) genes were all cultivated on a medium containing sufficient iron for 10 days. Then, they were transferred to either Fe-sufficient or Fe-deficient medium for 3 days and immediately harvested. The seedlings were collected and processed for immunoprecipitation of MYB4-Flag, and subsequently, phosphorylation detection was performed using anti-Phosphoserine.

4. To assess the specificity of MYB4 function in Fe deficiency responses, the authors should examine the sensitivity of the myb4 mutant to other micronutrient deficiencies (e.g., zinc, copper, or manganese), rather than responses to metal excess. Supplementary Figure 1, which focuses on metal toxicity, could be removed.

Response: We thank the reviewer for this valuable suggestion. We agree that testing the response of the myb4 mutant to other micronutrient deficiencies provide a more appropriate assessment of the specificity of MYB4 function in Fe-deficiency signaling. Accordingly, we performed additional experiments examining the growth of the *myb4-1* mutant under manganese (Mn)-, copper (Cu)-, and zinc (Zn)-deficient

conditions. In contrast to the enhanced tolerance observed under Fe deficiency, the *myb4-1* mutant did not display obvious phenotypic differences compared with Ler plants under Mn-, Cu-, and Zn-deficient conditions (Fig. 3). These results indicate that MYB4 does not broadly regulate micronutrient deficiency responses but instead functions more specifically in the Fe-deficiency signaling pathway. Based on the reviewer's suggestion, we have removed the previous Supplementary Figure 1. In its place, we have included new supplementary data showing the responses of *myb4-1* to Mn-, Cu-, and Zn-deficiency conditions. We have also revised the corresponding Results and Discussion sections accordingly.

A corresponding sentence has been added to the Results section as follows: "To further evaluate the specificity of *MYB4* function in micronutrient deficiency responses, we examined the growth of *myb4-1* mutants under Mn-, Cu-, and Zn-deficient conditions. Unlike the enhanced tolerance observed under Fe deficiency stress, *myb4-1* mutants showed no obvious phenotypic differences compared with wild-type plants under Mn-, Cu-, and Zn-deficient conditions, suggesting that MYB4 primarily functions in the regulation of Fe-deficiency responses."

A corresponding sentence has been added to the Discussion section as follows: "Although MYB transcription factors have been implicated in diverse metal stress responses, our additional analyses showed that *myb4-1* mutants did not display obvious phenotypic alterations under Mn-, Cu-, and Zn-deficient conditions. In contrast, *myb4* mutants consistently exhibited enhanced tolerance specifically under Fe deficiency stress, supporting the conclusion that MYB4 primarily functions in the FIT-dependent Fe-deficiency signaling pathway rather than in general micronutrient deficiency responses." Please refer to the revised manuscript for details (Lines 217-223, Lines 559-562, Figure EV2).

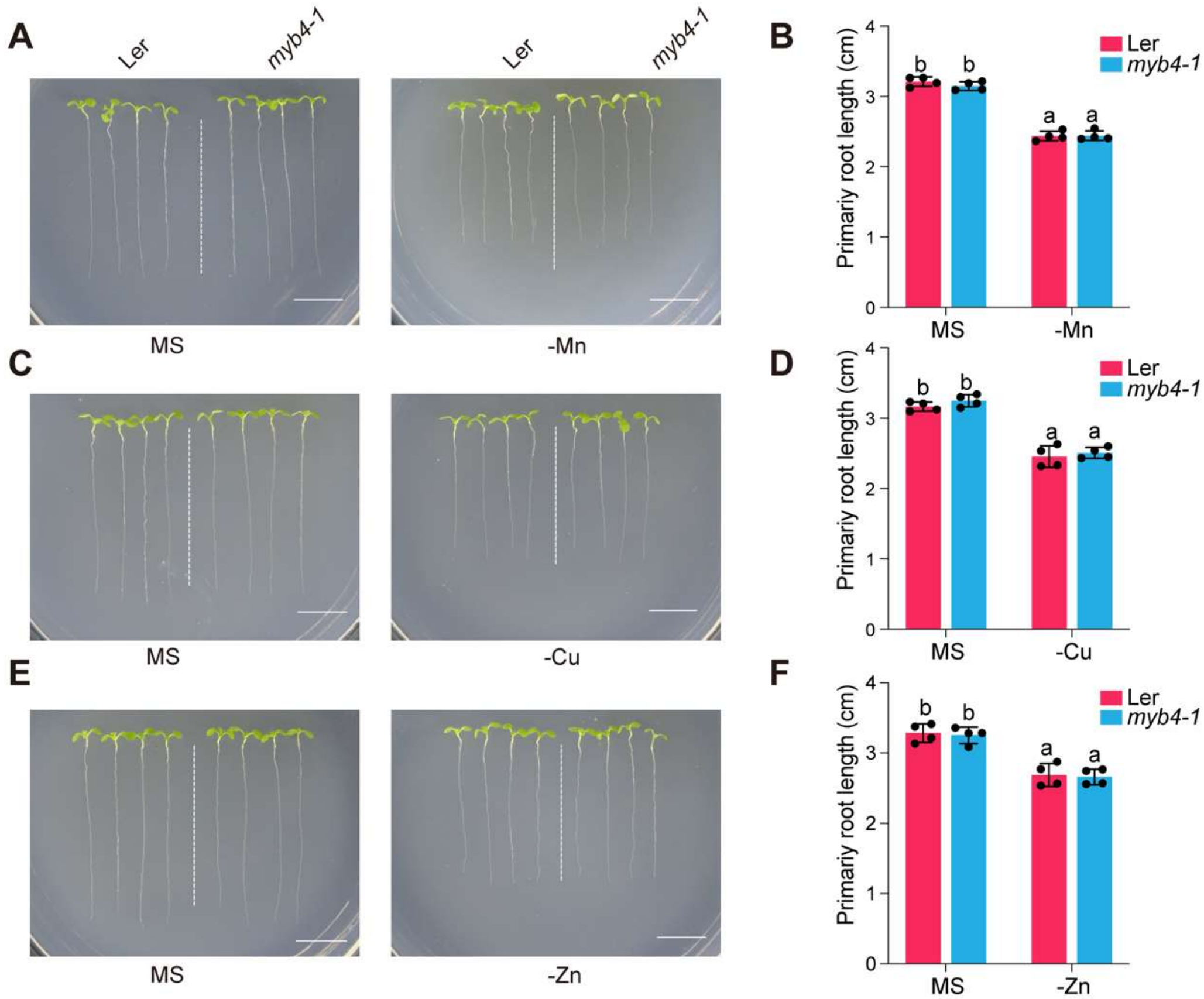

Figure 3. Phenotypes of *myb4-1* mutants under Mn-, Cu-, or Zn-deficient conditions. (A) Analysis of Mn-deficiency tolerance in Ler and *myb4-1* seedlings. Bar= 1 cm. **(**B) Root length of plants described in A. **(**C) Analysis of Cu-deficiency tolerance in Ler and *myb4-1* seedlings. Bar= 1 cm. **(**D) Root length of plants described in C. **(**E) Analysis of Zn-deficiency tolerance in Ler and *myb4-1* seedlings. Bar= 1 cm. **(**F) Root length of plants described in E. All data were subjected to at least three independent replicate experiments, each with three biological replicates. All data are presented as means ± SD. Statistical significance was determined by Tukey's test, with different lowercase letters indicating significant differences ($P < 0.05$).

5. Because the Ser4 mutation affects MYB4 stability, the authors need to distinguish between effects on MYB4 protein abundance and effects on MYB4-FIT interaction in Fig. 9. A pull-down assay would be more appropriate to determine whether MYB4 phosphorylation directly influences its interaction with FIT.

Response: We thank the reviewer for this insightful suggestion. We agree that the reduced interaction observed in the Co-IP and LCI assays could potentially be influenced by differences in MYB4 protein abundance caused by the Ser4 mutation. To address this concern, we performed additional in vitro pull-down assays using purified recombinant proteins to directly examine whether phosphorylation at Ser4 affects the MYB4-FIT interaction independently of protein stability. The pull-down assays demonstrated that wild-type MYB4, the phosphorylation-deficient mutant $MYB4^{S4A}$, and the phosphorylation-mimicking mutant $MYB4^{S4D}$ exhibited comparable binding capacities toward FIT, indicating that alteration of the Ser4 phosphorylation status does not directly impair the physical association between MYB4 and FIT (Fig. 4). These findings suggest that the altered interaction signals observed in planta are primarily attributable to phosphorylation-dependent changes in MYB4 protein stability and accumulation, rather than a direct effect of Ser4 phosphorylation on the intrinsic binding affinity between MYB4 and FIT. We have incorporated these new results into the revised manuscript and clarified in the Discussion that MPK3-mediated phosphorylation mainly regulates MYB4 abundance through protein destabilization, thereby indirectly modulating the availability of MYB4 to associate with FIT.

A corresponding sentence has been added to the Results section as follows: "To determine whether Ser4 phosphorylation directly affects the intrinsic binding ability of MYB4 toward FIT, we further performed in vitro pull-down assays using purified recombinant proteins. The results showed that wild-type MYB4, the dephosphorylation-mimicking mutant $MYB4^{S4A}$, and the phosphorylation-mimicking mutant $MYB4^{S4D}$ exhibited comparable binding capacities toward FIT, indicating that alteration of the Ser4 phosphorylation status does not directly impair the physical association between MYB4 and FIT. These findings suggest that the reduced interaction observed in planta is more likely attributable to phosphorylation-dependent changes in MYB4 protein abundance or stability rather than altered intrinsic binding affinity." Please refer to the revised manuscript for details (Lines 493-502, Figure EV5D).

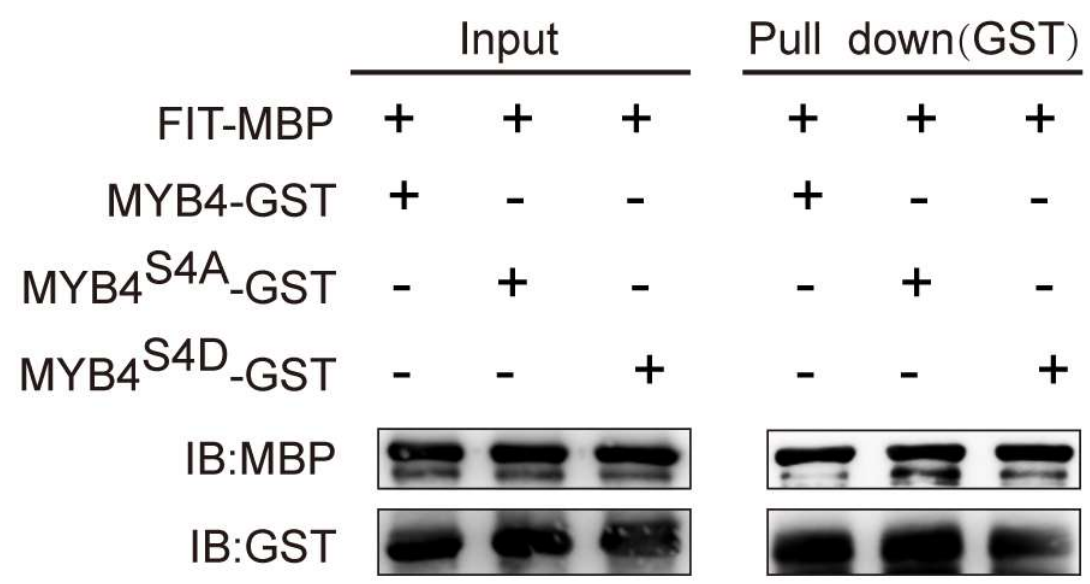

Figure 4 The effects of the Ser4A and Ser4D mutations on the interaction between MYB4 and FIT were examined using pull-down assays. For the pull-down assays, recombinant FIT-MBP protein was incubated with MYB4-GST, MYB4^{S4A}-GST, or MYB4^{S4D}-GST proteins at 4° C for 5 h.

6. Given that Fe deficiency strongly induces MPK3 protein accumulation, it is unclear whether the increased MPK3 phosphorylation shown in Fig. 6I reflects elevated protein abundance and/or enhanced kinase activity. This issue should be clarified.

Response: We thank the reviewer for this important comment. We agree that the increased kinase signal observed in the original Fig. 6I could potentially reflect elevated MPK3 protein abundance, enhanced kinase activity, or both, since Fe deficiency strongly induces MPK3 accumulation (Fig. 6H). To clarify this issue, we performed an additional kinase assay using equivalent amounts of MPK3 protein from Fe-sufficient and Fe-deficient seedlings. Consistent with our original results, MPK3 kinase activity was higher under Fe-deficient conditions when analyzed using equal amounts of total protein (Fig. 5). Importantly, even after normalizing MPK3 protein abundance, MPK3 isolated from Fe-deficient seedlings still exhibited substantially higher kinase activity than MPK3 from Fe-sufficient seedlings. These results demonstrate that Fe deficiency not only increases MPK3 protein accumulation but also enhances its intrinsic kinase activity. We have incorporated these new data and revised the corresponding text in the Results section. Therefore, the increased MPK3 signaling output under Fe deficiency is attributable to both elevated protein abundance and increased kinase activation. A corresponding sentence has been added to the Results section as follows: “To further elucidate the functional involvement of

MPK3 in the plant response to Fe-deficiency stress, we analyzed changes in MPK3 protein levels in wild-type Arabidopsis seedlings under Fe-deficient conditions. The results showed that MPK3 protein accumulation was markedly increased under Fe deficiency stress (Fig. 6H). Consistent with this observation, kinase assays performed using equal amounts of total protein revealed substantially higher MPK3 kinase activity in Fe-deficient seedlings than in Fe-sufficient seedlings (Fig. 6I). To further determine whether this increase was solely attributable to elevated MPK3 abundance, we performed an additional kinase assay using equivalent amounts of MPK3 protein from Fe-sufficient and Fe-deficient seedlings. Under these normalized conditions, MPK3 derived from Fe-deficient seedlings still exhibited markedly higher kinase activity than MPK3 from Fe-sufficient seedlings. These results indicate that Fe deficiency not only promotes MPK3 protein accumulation but also enhances its intrinsic kinase activity. Therefore, the increased MPK3 signaling output under Fe deficiency is attributable to both elevated protein abundance and increased kinase activation." Please refer to the revised manuscript for details (Lines 363-371).

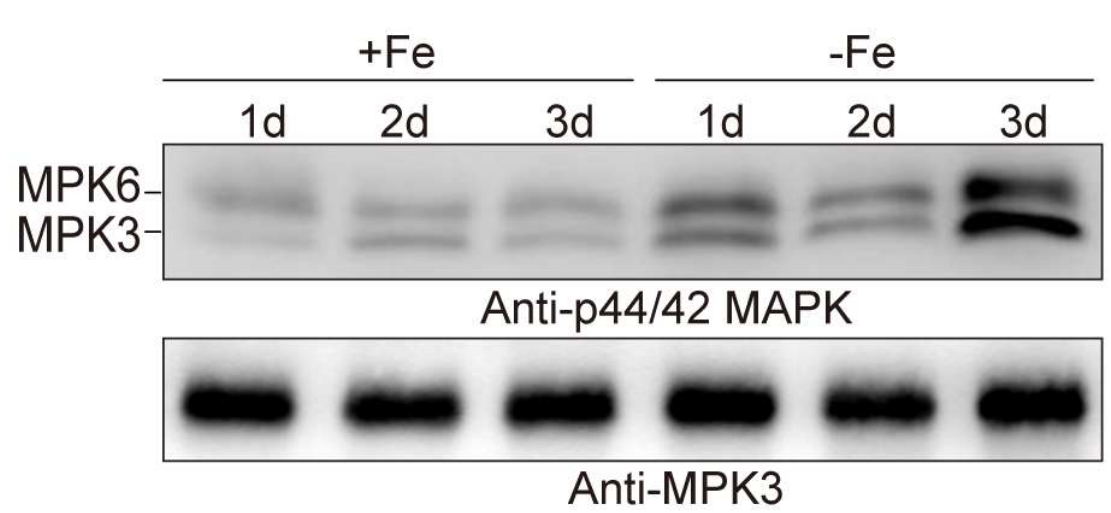

Figure 5. Fe deficiency enhances MPK3 kinase activity. Proteins were extracted from wild-type Arabidopsis seedlings grown under Fe-sufficient or Fe-deficient conditions and subjected to gel-based kinase assays. MPK3 kinase activity was elevated under Fe-deficient conditions. MAPK activation was verified by immunoblotting with an anti-phospho-p44/42 MAPK antibody. Equivalent amounts of MPK3 protein were used for kinase activity measurements to evaluate changes in intrinsic kinase activity.

7. The reference citation requires substantial improvement. Several important studies on Fe deficiency responses are missing, and some statements in the Introduction are not supported by the cited references (e.g., lines 44, 65, and 117).

Response: We thank the reviewer for this constructive suggestion. We have substantially revised the Introduction and reference citations throughout the manuscript to improve both the completeness and accuracy of the literature support. Please refer to the revised manuscript for details (Lines 45-49, 54, 58, 71, 121-124, 126).

8. The Discussion section should be shortened and made more concise. The authors are encouraged to integrate their findings with previous studies and provide broader insights, rather than reiterating their results.

Response: We appreciate the reviewer's insightful suggestion. In response, we substantially revised and condensed the Discussion section to improve its clarity and conceptual focus. Specifically, we removed repetitive descriptions of experimental results, reduced figure-by-figure interpretation, and integrated our findings more extensively with previous studies on FIT regulatory networks and MAPK-mediated post-translational regulation. We further expanded the broader biological implications of the MPK3–MYB4–FIT module in balancing Fe acquisition and Fe toxicity, thereby strengthening the conceptual significance of the study. Please refer to the revised Discussion section for details (Lines 521-539, Lines 579-608).

9. In Fig. 2, the order of the figure legend does not correspond to the panels in the figure and should be corrected.

Response: Thank you for the reviewer's comments. We have corrected it. Please refer to the revised manuscript for details (Lines 185, 187, 190, 211-214, 1180-1185).

10. The promoters used to drive MYB4-Flag expression in Figs. 7 and 8 are not described and should be clearly indicated.

Response: Thank you for the reviewer's comments. The promoters used to drive MYB4-Flag expression in Figs 7 and 8 have been indicated. Please refer to the revised manuscript for details (Lines 412, 432-433, 443-449, 1271, 1278-1279).

11. Fe deficiency phenotypes are often difficult to observe on standard MS medium due to Fe contamination. The authors should clarify whether a modified MS medium was used and provide relevant details in the Methods section.

Response: We thank the reviewer for this valuable comment. To minimize the potential influence of residual Fe contamination in standard MS medium, Fe-deficiency treatments were performed using a modified MS medium. Specifically, Arabidopsis seeds were surface-sterilized with 0.1 M $HgCl_2$ for 1 min, rinsed five times with sterile distilled water, and then sown on either Fe-sufficient (+Fe) MS medium or Fe-deficient (-Fe) MS medium lacking Fe-EDTA. To further reduce the availability of trace Fe that may be present in the agar, 5 mM ferrozine was added to the Fe-deficient medium to chelate residual Fe. The pH of all media was carefully adjusted to 5.8 before autoclaving. These experimental details have now been added to the Materials and Methods section of the revised manuscript. Please refer to the revised manuscript for details (Lines 694-701).

Responses to comments of the Reviewer 2:

This manuscript examines a regulatory module in which MPK3 phosphorylates the R2R3-MYB transcription factor MYB4, promoting its destabilization and thereby relieving repression of FIT-bHLH38/39 complexes to enhance iron uptake in Arabidopsis. The authors assemble an impressive and diverse experimental toolkit encompassing genetics, biochemistry, cell biology, and transcriptional assays, which represents a clear strength of the work. The identification of MYB4 as an additional negative regulator acting on FIT is potentially interesting and contributes another layer to the increasingly complex post-translational control of iron acquisition. With improved genetic coherence, more rigorous mechanistic validation-particularly regarding kinase specificity and in planta phosphorylation-and a more restrained framing of conceptual advance, this study could ultimately represent a meaningful contribution to the field.

Response: We sincerely appreciate the insightful and constructive comments provided by the reviewers on our manuscript. We are particularly grateful for their recognition of the extensive experimental approach employed in this study, as well as the potential

significance of the MPK3–MYB4–FIT regulatory module in iron homeostasis. In response to the reviewers' suggestions, we have substantially revised the manuscript to strengthen the genetic and mechanistic coherence of our research and to more comprehensively present its theoretical advances. These revisions have significantly improved the rigor and clarity of the paper, and we believe the revised version better reflects the scope and importance of our work. The details are as follows.

1. From a conceptual perspective, however, the regulatory logic proposed here largely conforms to an already well-developed paradigm in which FIT activity is fine-tuned by multiple interacting repressors, competitive protein-protein interactions, and regulated proteolysis. While the involvement of MYB4 expands this regulatory network, the manuscript occasionally presents the advance in broader terms than warranted by the data. The Discussion would benefit from a sharper and more explicit definition of what mechanistically distinguishes MYB4 from previously described FIT regulators, rather than emphasizing novelty in a general sense. A more focused positioning would strengthen the conceptual impact.

Response: We thank the reviewer for this insightful and constructive comment. We agree that FIT activity is regulated through an increasingly complex network involving multiple interacting repressors, competitive protein-protein interactions, and post-translational regulatory mechanisms. We also appreciate the reviewer’s suggestion that the Discussion should more clearly define the mechanistic contribution of MYB4 within this established regulatory framework, rather than emphasizing novelty in a broad sense. In response to this comment, we have carefully revised the Discussion to provide a more focused conceptual positioning of our findings. Specifically, we toned down several generalized statements regarding novelty and instead emphasized the mechanistic features that distinguish MYB4 from previously reported FIT repressors. First, we now clarify that MYB4 functions through a mechanistically distinct mode of inhibition. Our interaction domain-mapping analyses revealed that MYB4 and bHLH38/39 compete for an overlapping binding interface within the FIT-M region, thereby directly interfering with assembly of the transcriptionally active FIT-bHLH complex. We further

strengthened this interpretation by explicitly integrating the EMSA and ChIP-qPCR results, which demonstrate that MYB4 attenuates both DNA-binding activity of the FIT-bHLH complex and FIT enrichment at Fe-responsive promoters. Second, we revised the Discussion to highlight that MYB4 is dynamically regulated through MPK3-mediated phosphorylation. We now emphasize that phosphorylation at Ser4 simultaneously weakens MYB4-FIT interaction and promotes MYB4 ubiquitin-mediated degradation, thereby functioning as a phosphorylation-responsive molecular switch that relieves repression of FIT activity under Fe-deficient conditions. We believe this regulatory logic provides a mechanistic distinction from previously described FIT repressors. Accordingly, throughout the revised manuscript, we replaced several broad "novel regulatory mechanism" statements with more precise descriptions indicating that MYB4 represents an additional phosphorylation-responsive layer of regulation within the established FIT-centered network. Please refer to the revised manuscript for details (Lines 579-593).

2. Several key conclusions currently rest on incomplete mechanistic support and would benefit from tighter causal linkage. A major issue concerns genetic consistency. The study employs different myb4 alleles in distinct ecotype backgrounds (Ler and Col), raising the possibility of background-dependent effects, especially given the documented natural variation in iron responses among Arabidopsis accessions. Indeed, root growth responses under iron deficiency appear inconsistent across figures and backgrounds: Ler plants show reduced root growth under −Fe conditions in Figure 2, whereas Col-0 exhibits different behavior between Figures 2 and 6. These discrepancies raise concerns about experimental reproducibility and whether iron deficiency responses are being evaluated in a comparable manner across experiments. Ideally, the authors should generate and analyze a myb4 allele in a single standardized background (e.g., CRISPR/Cas9 in Col-0). At minimum, potential background effects should be explicitly addressed, and key molecular and physiological phenotypes should be validated across genotypes.

Response: We thank the reviewer for raising this important point regarding genetic

background consistency and the use of different *myb4* alleles in distinct ecotypes. We fully agree that genetic background control is essential for robust phenotypic interpretation, particularly in the context of documented natural variation in iron deficiency responses among Arabidopsis accessions. In our study, both Ler and Col genetic contexts involve bona fide loss-of-function alleles. The original *myb4* mutant was isolated in the Landsberg erecta (Ler) background (Wang et al., 2020, The Plant Journal). To obtain a corresponding allele in the Columbia background, this mutant was backcrossed five consecutive generations with wild-type Col-0 plants prior to experimental use. The *myb4-2* line used in our study therefore carries a uniform Col-0 genetic background, minimizing the possibility of residual Ler-derived genomic regions. We will explicitly describe this backcrossing procedure in the revised Methods section to ensure full transparency regarding the genetic origin of the material. Importantly, the major physiological and molecular phenotypes reported in our manuscript were obtained using this well-characterized Col-0 *myb4-2* knockout line. Although Ler and Col-0 may differ in the absolute magnitude of root growth inhibition under −Fe conditions, the directionality of MYB4-dependent effects is consistent: loss of MYB4 enhances Fe deficiency responses across genetic contexts. Key molecular readouts, including altered expression of Fe uptake genes and modulation of FIT-dependent transcriptional activity, were reproducibly observed in the Col-0 background. All key physiological and molecular analyses presented in our manuscript were performed using this well-characterized Col-0 **myb4-2** mutant line. We will explicitly describe this backcrossing procedure in the Methods section to ensure transparency regarding the genetic origin of the mutant. We believe that this approach adequately addresses the reviewer's concern and strengthens the reproducibility of our conclusions. Regarding the apparent differences in root growth observed in the figures, to ensure experimental consistency and more rigorously address the reviewers' concerns, we conducted phenotypic experiments under standardized and fully consistent conditions (including synchronized seedling development, uniform medium composition, and consistent duration of iron treatment). This allows for direct comparison of different genotypes within the same

experimental batch and eliminates potential batch-specific variability. The revised manuscript has incorporated the results from these parallel validation experiments, and the experimental setup is clearly documented in the "Methods" section and figure legends. We believe that this additional validation will enhance the reproducibility and reliability of the physiological analysis. Please refer to the revised manuscript for details (Lines 683-688, Fig 2, 6).

3. Related to this point, the phenotypic focus of the study lacks clarity. Much of the mechanistic framework centers on FIT regulation and root-associated iron uptake processes (IRT1, FRO2), yet primary root growth is not systematically analyzed or integrated, while chlorophyll accumulation is used as a major physiological readout without a clear conceptual justification in the Introduction. If chlorophyll content is intended as a central outcome, additional markers linked to chloroplast function, iron partitioning, or hormonal regulation (e.g., ethylene signaling) would help reconcile the molecular findings with the physiological interpretation. As currently presented, it is difficult to fully assess the biological relevance and internal consistency of the phenotypes attributed to MYB4.

Response: We thank the reviewer for this important comment regarding the conceptual coherence of the phenotypic analyses and the integration of molecular and physiological findings. Our mechanistic framework primarily focuses on the regulation of FIT activity and downstream iron uptake genes (*IRT1*, *FRO2*) in roots. The physiological phenotypes were intended to reflect the systemic consequences of altered root iron acquisition capacity rather than to suggest that MYB4 directly regulates chloroplast-specific processes. To clarify this conceptual linkage, we will strengthen the connection between the molecular mechanism and physiological outcomes in the revised manuscript. Specifically, we will explicitly state in the Introduction: "Because Fe availability directly affects chlorophyll biosynthesis and photosynthetic capacity, chlorophyll content serves as a widely used integrative indicator of plant iron nutritional status." Under iron deficiency, impaired root iron uptake rapidly reduces Fe availability in aerial tissues, leading to compromised chlorophyll biosynthesis and leaf chlorosis. In this context, altered chlorophyll

accumulation in the myb4 mutant reflects systemic changes in iron nutritional status resulting from modulation of FIT-dependent iron uptake in roots. We agree that this root-to-shoot linkage was not sufficiently articulated in the original version. The Introduction sections have been revised to explicitly present chlorophyll content as a downstream, comprehensive indicator of whole-plant iron nutrition. Please refer to the revised manuscript for details (Lines 45-50).

4. The authors convincingly show that MYB4 does not directly bind the promoters of IRT1 or FRO2 and instead interferes with FIT-bHLH complex formation. However, MYB4 is a transcription factor with well-established regulatory roles in other stress and metabolic pathways, raising the possibility that it may influence additional iron-responsive or stress-related genes indirectly. Without broader expression profiling or targeted analysis beyond the FIT module, it remains difficult to define the specificity and physiological scope of MYB4 action in iron homeostasis. Expanding this analysis would substantially strengthen the biological interpretation and avoid an overly narrow mechanistic framing.

Response: We thank the reviewer for this insightful comment. We agree that MYB4 is a multifunctional transcription factor previously implicated in diverse stress responses and metabolic pathways. Our objective in this study was not to comprehensively define the entire MYB4-dependent transcriptional network, but rather to elucidate the molecular mechanism by which MYB4 regulates the iron-deficiency response.Several lines of evidence support the conclusion that MYB4 primarily acts through the FIT module under iron-deficient conditions. First, MYB4 does not directly bind the promoters of the key Fe uptake genes IRT1 and FRO2. Instead, MYB4 physically interacts with FIT and competitively interferes with FIT-bHLH38/39 heterodimer formation, thereby suppressing FIT-dependent transcriptional activation. Second, the enhanced expression of IRT1 and FRO2, elevated ferric chelate reductase activity, and increased Fe accumulation observed in the myb4 mutants are all consistent with enhanced FIT activity. Third, the Fe-deficiency-tolerant phenotype of myb4 was completely suppressed in the fit background, indicating that MYB4 functions

genetically through FIT.To further address the reviewer’s concern, we conducted a study on the expression of the two previously reported MYB4 target genes - ADT6 and MAN3 (for instance, ADT6 is involved in the biosynthesis of flavonoids, and MAN3 is involved in cadmium stress) - under conditions of iron sufficiency and iron deficiency. As shown in the Fig. 6, ADT6 expression was elevated whereas MAN3 expression was reduced in the *myb4-2* mutant compared with wild type, confirming that MYB4 retains its established regulatory functions under these conditions. However, the expression patterns of these genes did not correlate with the enhanced Fe-deficiency tolerance observed in *myb4* mutants and were not specifically associated with the FIT-dependent Fe uptake pathway. These findings suggest that although MYB4 can regulate additional targets, its primary detectable role in iron homeostasis under Fe-deficient conditions is mediated through modulation of FIT complex activity. In the revised discussion and results sections, we have clearly stated this point and explicitly pointed out that our conclusions are restricted to the FIT-dependent branch of the iron acquisition network and do not exclude additional MYB4 functions in other physiological contexts. Please refer to the revised manuscript for details (Lines 316-330, 539-572).

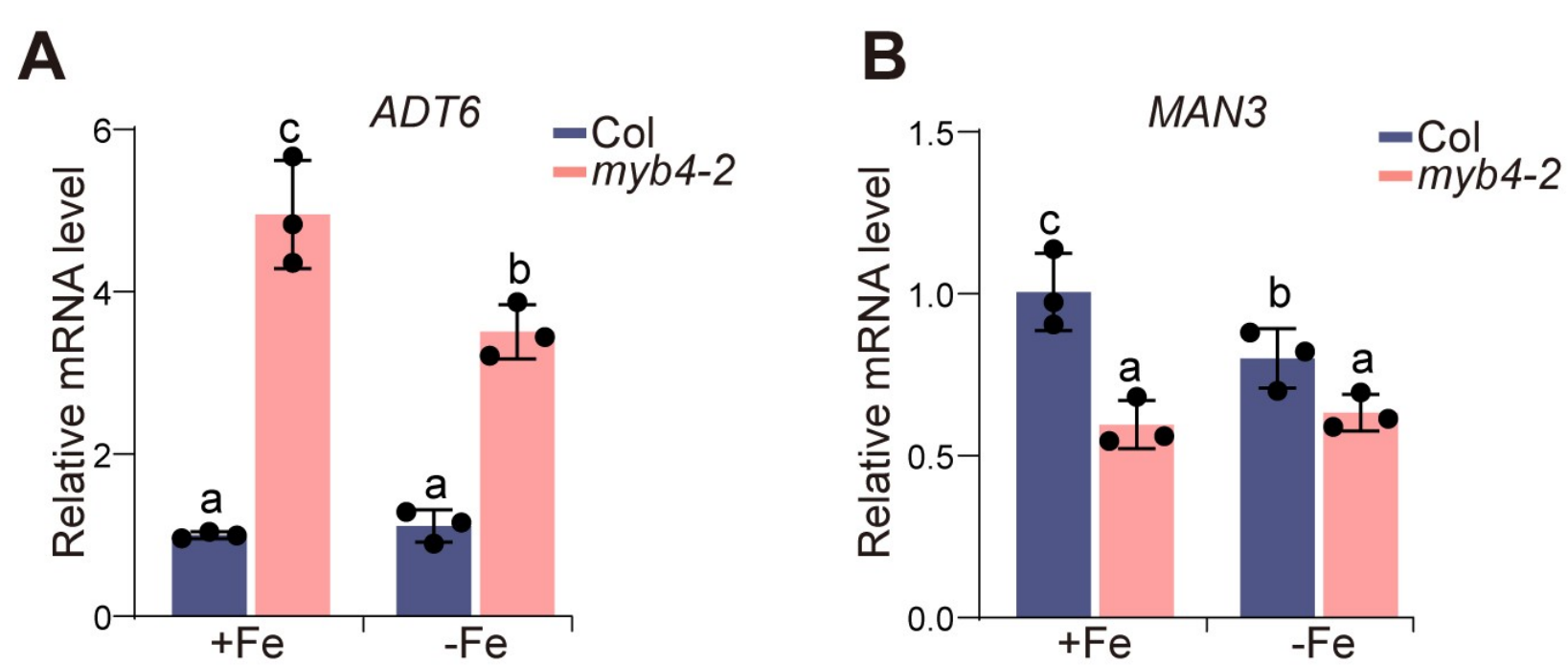

Figure 6 Quantitative analysis of ADT6 and MAN3 transcript levels in Col and *myb4-2* plants under both normal and Fe-deficient conditions. (A, B) Expression levels of ADT6 (A) and MAN3 (B) in Col and *myb4-2* plants were detected by RT-qPCR under both normal and Fe-deficient conditions. Arabidopsis seedlings were grown under both normal and Fe-deficiency conditions, and roots were harvested for RNA extraction followed by RT-qPCR analysis. *ACTIN8* was used as an internal control. All data were subjected to at least three independent replicate experiments,

each with three biological replicates. All data are presented as means±SD. Statistical significance was determined by Tukey's test, with different lowercase letters indicating significant differences ($P < 0.05$).

5. Another concern is the reliance on transient assays in tobacco for several mechanistic conclusions. While such systems are appropriate for exploratory interaction testing, conclusions related to transcriptional regulation, protein stability, and signaling hierarchy should ideally be corroborated in Arabidopsis, particularly for a pathway involving spatially coordinated and long-distance iron signaling. Tobacco-based assays should therefore be presented as supportive rather than definitive unless validated in the native context.

Response: We sincerely thank the reviewer for this thoughtful comment. We fully agree that transient expression assays in *N. benthamiana*, while highly useful for rapid biochemical and interaction analyses, may not fully reflect the physiological and spatial complexity of Fe signaling in *Arabidopsis.* In the original manuscript, some interpretations derived from tobacco transient assays may indeed have been presented too definitively. We have carefully revised the manuscript to clarify that these assays provide supportive biochemical evidence rather than definitive proof of signaling hierarchy in the native *Arabidopsis* context. We replaced overly definitive wording such as "demonstrates" or "establishes" with more cautious descriptions, including "supports" or "suggests"; we explicitly indicate when conclusions are derived from transient *N. benthamiana* assays. Please refer to the revised manuscript for details (Lines 268-270, 284, 493-502, 515-517).

6. The assignment of MPK3 as the relevant upstream kinase is plausible but not yet fully resolved. The increase in MPK3 abundance and activity under iron deficiency is convincing, and in vitro phosphorylation assays support MYB4 as a potential substrate. However, the substantial functional overlap between MPK3 and MPK6 necessitates a more rigorous assessment of kinase specificity. It remains unclear whether MPK6 can phosphorylate MYB4 in vitro or whether MYB4 phosphorylation

and stability are altered in mpk6 mutants. Moreover, direct evidence that MYB4 phosphorylation in planta depends on MPK3 is currently missing. Demonstrating reduced phospho-MYB4 levels or altered MYB4 turnover in an mpk3 background would substantially strengthen the causal argument. At present, the proposed sequence linking iron deficiency to MPK3 activation and MYB4 degradation remains largely correlative. A temporal analysis would help establish directionality and strengthen causality. The upstream signal triggering MPK3 activation is not addressed and should be acknowledged more explicitly as an unresolved mechanistic gap.

Response: We fully agree that MPK3 and MPK6 often exhibit overlapping functions in various stress responses, and careful evaluation of kinase specificity is important. Our identification of MPK3 was initiated through an unbiased yeast two-hybrid screening using MYB4 as bait, in which MPK3, but not MPK6, was recovered as an interacting partner. Subsequent Y2H, BiFC, and Co-IP assays consistently confirmed a specific physical interaction between MPK3 and MYB4. Importantly, our genetic and biochemical analyses further support a functional dependency of MYB4 regulation on MPK3 in planta. In the mpk3 background, MYB4 protein degradation was markedly attenuated in cell-free degradation assays (Fig. 7E), and MYB4 protein accumulation was significantly increased compared to wild type under both normal and Fe-deficient conditions (Fig. 7F). Moreover, mutation of the identified phosphorylation site (Ser4) abolished MPK3-mediated phosphorylation in vitro and substantially altered MYB4 stability and ubiquitination in vivo. The phospho-mimic and phospho-dead transgenic lines further demonstrate that Ser4 phosphorylation is essential for MYB4 turnover and biological function under Fe deficiency stress. To further confirm the conclusion that MPK3 can phosphorylate the Ser4 site of MYB4 in plants, we have measured the phosphorylation levels of MYB4 in the wild type (Col-0) and the *mpk3* mutant background. The results show a clear reduction in the phosphorylation signal of MYB4 in mpk3 compared with Col plants under Fe-deficient conditions (Figure 7A). This trend indicates that MPK3 is a major kinase responsible for MYB4 phosphorylation in vivo. Furthermore, to assess the effect of the S4A mutation, we examined the phosphorylation level of MYB4^{S4A} in transgenic

plants. It was found that the phosphorylation signal of $MYB4^{S4A}$ was significantly lower than that of the wild-type MYB4 (Figure 7B). This result further confirmed that Ser4 is the main phosphorylation site of MYB4 in plants. While we cannot formally exclude the possibility that MPK6 may phosphorylate MYB4 under certain conditions, we did not detect physical interaction between MPK6 and MYB4 in our screening assays. Given that MAPK substrate specificity frequently depends on docking interactions, the absence of detectable interaction suggests that MPK3 is the primary kinase acting on MYB4 in the Fe-deficiency context examined here. We have made revisions to the results and discussion sections. Please refer to the revised manuscript for details (Lines 405-415, 594-608, Figure EV5).

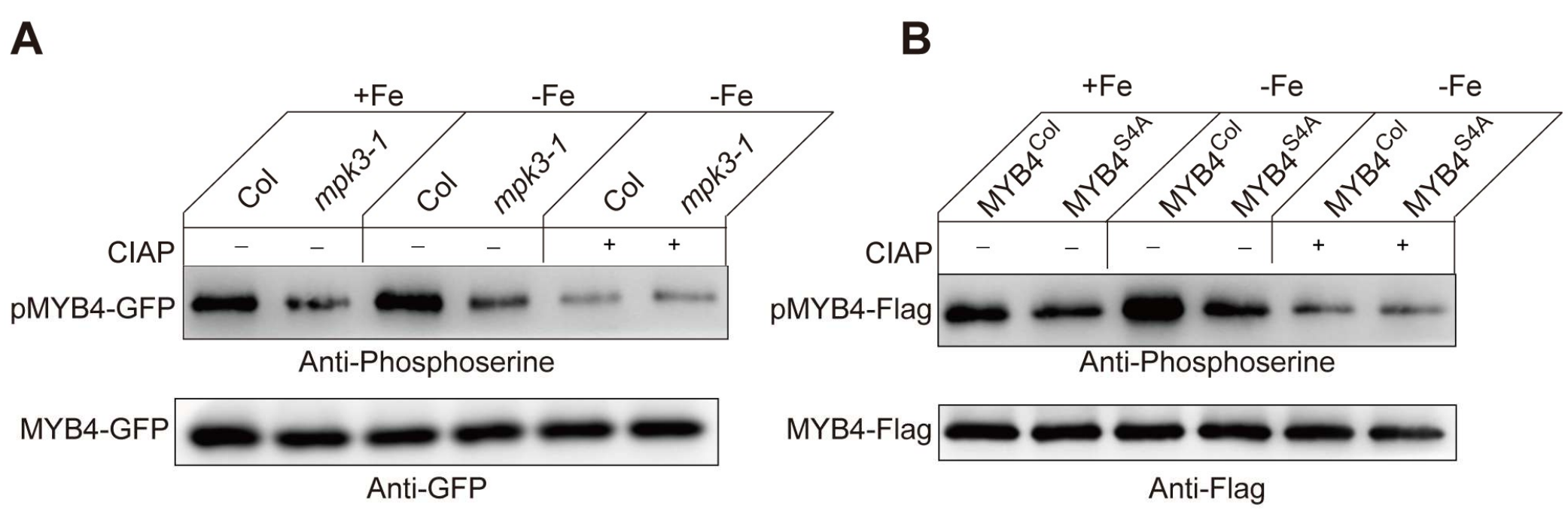

Figure 7. Under Fe-deficiency stress, MPK3 regulates MYB4 accumulation through phosphorylating MYB4 at Ser4. (A) The effect of *mpk3-1* mutation on the phosphorylation of MYB4. The seedlings of the Col plants carrying the MYB4-GFP gene and the *mpk3-1* plants were all cultivated on a medium with sufficient Fe for 10 days. Then, they were transferred to either Fe-sufficient or Fe-deficient medium for 3 days and immediately harvested. The seedlings were collected and processed for immunoprecipitation of MYB4-GFP, and subsequently, phosphorylation detection was performed using anti-Phosphoserine. (B) Effect of Ser4A mutation on MYB4 phosphorylation. The seedlings in the *myb4-2* background that carried the MYB4-Flag ($MYB4^{Col}$) or $MYB4^{S4A}$-Flag ($MYB4^{S4A}$) genes were all cultivated on a medium containing sufficient iron for 10 days. Then, they were transferred to either Fe-sufficient or Fe-deficient medium for 3 days and immediately harvested. The seedlings were collected and processed for immunoprecipitation of MYB4-Flag, and subsequently, phosphorylation detection was performed using anti-Phosphoserine.

7. The mechanistic treatment of MYB4 degradation is also incomplete. Although proteasome dependence and increased ubiquitination are demonstrated, the ubiquitination machinery itself is not explored and the data remain largely qualitative. If identification of the relevant E3 ligase(s) is beyond the scope of this study, this limitation should be clearly stated and the mechanistic claims tempered accordingly.

Response: We sincerely thank the reviewer for this insightful and constructive comment. We agree that, although our data support the involvement of ubiquitin-associated proteasomal degradation in MYB4 turnover, the upstream ubiquitination machinery responsible for this process has not yet been identified. In the current study, we focused primarily on establishing the functional relationship between MPK3-mediated phosphorylation and MYB4 destabilization under Fe-deficiency conditions. While our MG132 inhibition assays and ubiquitination analyses support a role for ubiquitin-associated degradation, we agree that the responsible E3 ubiquitin ligase and the detailed ubiquitination mechanism remain unresolved. Identification and mechanistic characterization of the relevant E3 ligase are important future directions but are beyond the scope of the present study. In response to the reviewer's suggestion, we have carefully revised the manuscript to temper several mechanistic statements regarding MYB4 ubiquitination and degradation. Specifically: we replaced definitive wording such as "promotes ubiquitination" with more cautious descriptions including "is associated with enhanced ubiquitination". We revised statements referring to a fully defined degradation mechanism; and we added a paragraph in the Discussion explicitly acknowledging that the E3 ligase responsible for MYB4 ubiquitination remain unknown. Please refer to the revised manuscript for details (Lines 450-452, 644-651).

8. Several aspects of data presentation and reporting require attention. Sample sizes should be stated consistently, the distinction between biological and technical replicates should be unambiguous, and statistical analyses should be uniformly reported. Minor labeling inconsistencies, figure cross-referencing errors, and language

issues detract from clarity and should be corrected to meet journal standards.

Response: We sincerely thank the reviewer for these careful and constructive comments regarding data presentation and manuscript clarity. We fully agree that consistent reporting and standardized presentation are essential for meeting the journal's rigor and formatting standards. We have thoroughly revised the manuscript to clearly distinguish biological replicates from technical replicates. We now consistently report sample sizes and replication information using standardized terminology. We also standardized the reporting of statistical analyses across all figure legends. The statistical methods, including one-way ANOVA followed by Tukey's multiple comparison test, are now described uniformly throughout the manuscript. Please refer to the revised manuscript for details (Lines 1027, 1190, 1119-1200, 1230, 1248, 1301, 1318, 1322, 1342-1343, 1361-1362, 1383-1384). In addition, we carefully re-examined the entire manuscript and corrected figure labeling inconsistencies, panel citation errors, and figure cross-referencing issues. Please refer to the revised manuscript for details (Lines 185-187, 211-214, 440, 447, 450, 466). We believe these revisions have substantially improved the overall clarity, rigor, and presentation quality of the manuscript and have brought it into better alignment with journal standards.

9. Finally, some interpretations appear overstated. Referring to the MPK3-MYB4-FIT pathway as a dominant or central regulatory mechanism seems premature given the multiplicity of parallel regulators already known to modulate FIT activity. Likewise, the reported responses to other metals (e.g., Co and Mn) are potentially interesting but are not mechanistically integrated into the main model and should either be developed further or de-emphasized.

Response: We thank the reviewer for this insightful and constructive comment. We fully agree that the regulation of FIT activity involves a complex network of multiple parallel regulators, and that our initial wording may have overstated the relative contribution of the MPK3–MYB4–FIT module. To address this concern, we have carefully revised the manuscript to moderate our interpretation. Specifically, we have

replaced terms such as “central regulatory mechanism” with more appropriate descriptions, such as “previously unrecognized regulatory module”. Please refer to the revised manuscript for details (Lines 29-32, 162-163). Regarding the reviewer’s comment on the responses to other metal ions, we agree that these observations were not sufficiently integrated into the main mechanistic framework. Therefore, we have revised the corresponding text to clearly present these results as preliminary observations. We have reduced their emphasis and now explicitly state that the underlying mechanisms remain to be elucidated in future studies. Please refer to the revised manuscript for details (Lines 217-223, 559-572).

Dear Prof. Cao

Thank you for the submission of your revised manuscript. We have now received the enclosed report from referee 1 who was asked to assess your full reply to all referee comments. I am happy to say that referee 1 supports the publication of your study now. Only a few editorial requests will need to be addressed before we can proceed with the official acceptance of your manuscript:

- In the Data Availability Section (DAS), the following sentence should be removed: "All data are available within the article, appendix data, and source data files. All materials used in this study are available by request from the corresponding author. Source data are provided with this paper." If I understand correctly, your study did not generate any new datasets that are publicly deposited, in this case, please mention this fact in the DAS.

- The Disclosure and Competing Interest Statement needs to be placed after the Acknowledgments.

- The ORCID IDs of the other 2 corresponding authors still need to be added to their personal profile pages.

- The author credits need to be removed from the ms file. All credits need to be entered during online ms submission.

- The REFERENCE format needs to be corrected: et al needs to be used after 10 author names. Please use the EMBO reports reference style.

- Please co-submit with your final ms a completed author checklist, which you can download from our author guidelines <https://link.springer.com/journal/44319/submission-guidelines> The completed author checklist will also be part of the transparent peer-review file.

- Some FUNDING INFO is missing in our online submission system: the Fundamental Research Funds for the Central Universities of China (grant no. JZ2025HGTB0185), the China Postdoctoral Science Foundation (Certificate Number: 2023M740962), and the Postdoctoral Fellowship Program of CPSF under Grant Number GZC20240364. Please add all funders with your next ms submission.

- Table EV1 is provided in the ms, but it needs to be uploaded as a separate file.

- The Materials and Methods section should include a separate Reagents and Tools Table file (listing key reagents, experimental models, software and relevant equipment and including their sources and relevant identifiers) and a Methods and Protocols section in which methods should be described using a step-by-step protocol format with bullet points. More information and a downloadable Table template is available in our guide to authors online: https://link.springer.com/journal/44319/submission-guidelines

- Our routine image analysis of to-be-accepted ms detected a possible reuse of cells within Figure EV3B and possibly empty cells in Figure 1B, Figure 5B and Figure 9E. Can you please clarify and correct ? If really no signal at all was detected, please add a cred cross across the figure panel and explain in the figure legend that no signal was obtained. Usually, some background signal is obtained.

* Figure Legends - Comments *

- Please note that the exact p values are not provided in the legends of figures 2B,D-L; 3B,D; 4B,C,G,I,J; 6B-G,K-N; 8B,D,F-I; 9F,G,H,I; EV-1A; EV-4G,H. Please provide exact values as reasonable.
- Please note that for the figure EV-4A,B, p-values and statistical tests are indicated in the legends. However, comparison for the same, "" different lowercase letters"" has not been represented in the figures. Please rectify this in the figures or legends as applicable.

I would like to suggest a small change to the ms title. Do you agree with this :

MPK3-mediated phosphorylation inactivates MYB4 to promote FIT-dependent iron acquisition

EMBO press papers are accompanied online by A) a short (1-2 sentences) summary of the findings and their significance, B) 2-3 bullet points highlighting key results and C) a synopsis image that is exactly 550 pixels wide and 300 pixels high. The synopsis image should provide a sketch of the major findings, like a graphical abstract. Please note that text needs to be readable at the final size. Please send us this information along with the final manuscript.

I look forward to seeing a final version of your manuscript as soon as possible. Please use this link to submit your revision:
https://embor.msubmit.net/cgi-bin/main.plex

Best regards,
Esther

Esther Schnapp, PhD
Senior Editor
EMBO reports

Referee #1:

The revised manuscript has improved substantially. The authors have adequately addressed my previous concerns through the performance of additional experiments, and I have no further comments.

As requested, I have reviewed the author's responses to Reviewer 2. I am satisfied that the authors have carefully and comprehensively addressed all the concerns raised and have responded appropriately to the reviewer's queries. In my view, the manuscript is of high quality an is fully worthy of publication in EMBO reports.

Dear Editor:

Thank you very much for your review of our manuscript. We decided to resubmit a revised version of our paper (EMBOR-2026-63836-T). In order to improve our research, we have revised the paper were carried out according to suggestions from editors. We have revised the manuscript in response to the editor's comments, and we have marked the revised position in red font. A point-by-point reply to the comments of editors are as follows:

Response to the Editor's Comments:

Thank you for resubmitting your manuscript and for making the requested changes. In checking your manuscript we noticed that the following must still be addressed before we can proceed with the editorial process:

1. Please check the possible image duplicate in Appendix Figure S2E, and comment and correct if applicable.

Response: Thank you for bringing this issue to our attention. We carefully examined the flagged panels. We confirmed that the apparent reuse of cells in Appendix Figure S2E was indeed due to an inadvertent copy-and-paste error during figure preparation. The duplicated image was mistakenly inserted when assembling the figure panel, although the experiments were independently performed as described. The duplicated image has now been replaced with the correct representative image from the original experiment. The updated results are presented in the revised manuscript. Please refer to the revised manuscript for details (Appendix Figure S2E).

We sincerely apologize for the error in Appendix Figure S2E and appreciate the Editor's careful image screening, which helped us identify and correct this issue. We have also rechecked all figure panels to ensure the accuracy and integrity of the revised manuscript.

2. In the Data Availability statement, please remove the sentence "All data are available within the article, appendix data, and source data files. All materials used in this study are available by request from the corresponding author. Source data are provided with this paper."

Response: Thank you for pointing this out. We have revised the Data Availability Statement accordingly and removed the sentence: “All data are available within the article, appendix data, and source data files. All materials used in this study are available by request from the corresponding author. Source data are provided with this paper.” Please refer to the revised manuscript for details (Lines 866-867).

3. In the reagents and tools table, please correct the citation for "Table S1" -- should this be Appendix Table S1?

Response: Thank you for pointing this out. We have carefully checked the citation in the Reagents and Tools Table and confirmed that the correct reference should be Appendix Table S1. We have revised the citation accordingly in the updated Reagents and Tools Table. Please refer to the revised Reagents and Tools Table for details.

4. Thank you for uploading a synopsis text and image. Please upload the synopsis image as a separate file, in png or jpg format, and sized exactly 550 pixels wide x 300 pixels high, which is the size in which synopsis images are published. Your paper has been placed back in the Author Approval Folder where you may access via the following link:https://embor.msubmit.net/cgi-bin/main.plex?el=A7Ij5BJaP5C1DXjP3F4A9ftdNXeMti0qdfnTJLG1rKZg5wY. Please make the corrections as specified above and resubmit your paper following the same steps as before.

Response: Thank you for your instruction. We have revised the submission accordingly. The synopsis image has now been prepared as a separate JPG file with the exact dimensions of 550 pixels in width and 300 pixels in height, as required. We have uploaded the corrected synopsis image separately in the Author Approval Folder and resubmitted the manuscript following the specified procedure. We appreciate your assistance and hank you for your careful guidance.

2nd Revision - Editorial Decision **13th Aug 2026**

Prof. Shuqing Cao
Hefei University of Technology
School of Food and Biological Engineering
193 Tunxi Road
Hefei, Anhui 230009
China

Dear Prof. Cao,

I am very pleased to accept your manuscript for publication in the next available issue of EMBO reports. Thank you for your contribution to our journal.

Your manuscript will be processed for publication by EMBO Press. It will be copy edited and you will receive page proofs prior to publication. Please note that you will be contacted by Springer Nature Author Services to complete licensing and payment information.

You may qualify for financial assistance for your publication charges - either via a Springer Nature fully open access agreement or an EMBO initiative. Check your eligibility: https://link.springer.com/journal/44319/how-to-publish-with-us

Should you be planning a Press Release on your article, please notify the production team via this form in order to coordinate publication and release dates: https://forms.cloud.microsoft/e/eH4A8JVP6g

If you have any questions, please do not hesitate to contact the Editorial Office. Thank you for your contribution to EMBO Reports.

Yours sincerely,

Esther Schnapp, PhD
Senior Editor
EMBO reports

>>> Please note that it is EMBO Reports policy for the transcript of the editorial process (containing referee reports and your response letter) to be published as an online supplement to each paper. If you do NOT want this, you will need to inform the Editorial Office via email immediately. More information is available here: https://link.springer.com/partners/embo-press/editorial-policies#Peer%20review

EMBO Press Author Checklist

Corresponding Author Name: Shuqing Cao	
Journal Submitted to: EMBO Reports	
Manuscript Number: EMBOR-2026-63836V2	

USEFUL LINKS FOR COMPLETING THIS FORM
The EMBO Journal - Author Guidelines
EMBO Reports - Author Guidelines
Molecular Systems Biology - Author Guidelines
EMBO Molecular Medicine - Author Guidelines

Reporting Checklist for Life Science Articles (updated January 2022)
This checklist is adapted from Materials Design Analysis Reporting (MDAR) Checklist for Authors. MDAR establishes a minimum set of requirements in transparent reporting in the life sciences (see Statement of Task: 10.31222/osf.io/9sm4x). Please follow the journal's guidelines in preparing your manuscript.
Please note that a copy of this checklist will be published alongside your article.

Abridged guidelines for figures
1. Data
The data shown in figures should satisfy the following conditions:
- the data were obtained and processed according to the field's best practice and are presented to reflect the results of the experiments in an accurate and unbiased manner.
- ideally, figure panels should include only measurements that are directly comparable to each other and obtained with the same assay.
- plots include clearly labeled error bars for independent experiments and sample sizes. Unless justified, error bars should not be shown for technical replicates.
- if n<5, the individual data points from each experiment should be plotted. Any statistical test employed should be justified.
- Source Data should be included to report the data underlying figures according to the guidelines set out in the authorship guidelines on Data Presentation.

2. Captions
Each figure caption should contain the following information, for each panel where they are relevant:
- a specification of the experimental system investigated (eg cell line, species name).
- the assay(s) and method(s) used to carry out the reported observations and measurements.
- an explicit mention of the biological and chemical entity(ies) that are being measured.
- an explicit mention of the biological and chemical entity(ies) that are altered/varied/perturbed in a controlled manner.
- the exact sample size (n) for each experimental group/condition, given as a number, not a range;
- a description of the sample collection allowing the reader to understand whether the samples represent technical or biological replicates (including how many animals, litters, cultures, etc.).
- a statement of how many times the experiment shown was independently replicated in the laboratory.
- definitions of statistical methods and measures:
  - common tests, such as t-test (please specify whether paired vs. unpaired), simple χ2 tests, Wilcoxon and Mann-Whitney tests, can be unambiguously identified by name only, but more complex techniques should be described in the methods section;
  - are tests one-sided or two-sided?
  - are there adjustments for multiple comparisons?
  - exact statistical test results, e.g., P values = x but not P values < x;
  - definition of 'center values' as median or average;
  - definition of error bars as s.d. or s.e.m.

Please complete ALL of the questions below.
Select "Not Applicable" only when the requested information is not relevant for your study.

Materials

Newly Created Materials	Information included in the manuscript?	In which section is the information available? (Reagents and Tools Table, Materials and Methods, Figures, Data Availability Section)
New materials and reagents need to be available; do any restrictions apply?	Not Applicable	

Antibodies	Information included in the manuscript?	In which section is the information available? (Reagents and Tools Table, Materials and Methods, Figures, Data Availability Section)
For **antibodies** provide the following information:<br>- Commercial antibodies: RRID (if possible) or supplier name, catalogue number and or/clone number<br>- Non-commercial: RRID or citation	Yes	Reagents and Tools Table, Materials and Methods,

DNA and RNA sequences	Information included in the manuscript?	In which section is the information available? (Reagents and Tools Table, Materials and Methods, Figures, Data Availability Section)
**Short novel DNA or RNA including primers, probes**: provide the sequences.	Yes	Reagents and Tools Table, Materials and Methods

Cell materials	Information included in the manuscript?	In which section is the information available? (Reagents and Tools Table, Materials and Methods, Figures, Data Availability Section)
**Cell lines:** Provide species information, strain. Provide accession number in repository **OR** supplier name, catalog number, clone number, and/**OR** RRID.	Not Applicable	
**Primary cultures:** Provide species, strain, sex of origin, genetic modification status.	Not Applicable	
Report if the cell lines were recently **authenticated** (e.g., by STR profiling) and tested for mycoplasma contamination.	Not Applicable	

Experimental animals	Information included in the manuscript?	In which section is the information available? (Reagents and Tools Table, Materials and Methods, Figures, Data Availability Section)
**Laboratory animals or Model organisms:** Provide species, strain, sex, age, genetic modification status. Provide accession number in repository **OR** supplier name, catalog number, clone number, **OR** RRID.	Not Applicable	
**Animal observed in or captured from the field:** Provide species, sex, and age where possible**.**	Not Applicable	
Please detail **housing and husbandry conditions**.	Not Applicable	

Plants and microbes	Information included in the manuscript?	In which section is the information available? (Reagents and Tools Table, Materials and Methods, Figures, Data Availability Section)
**Plants:** provide species and strain, ecotype and cultivar where relevant, unique accession number if available, and source (including location for collected wild specimens).	Yes	Materials and Methods, Figures
**Microbes:** provide species and strain, unique accession number if available, and source.	Not Applicable	

Human research participants	Information included in the manuscript?	In which section is the information available? (Reagents and Tools Table, Materials and Methods, Figures, Data Availability Section)
If collected and within the bounds of privacy constraints report on age, sex and gender or ethnicity for all study participants	Not Applicable	

Core facilities	Information included in the manuscript?	In which section is the information available? (Reagents and Tools Table, Materials and Methods, Figures, Data Availability Section)
If your work benefited from core facilities, was their service mentioned in the acknowledgments section?	Not Applicable	

Design

Study protocol	Information included in the manuscript?	In which section is the information available? (Reagents and Tools Table, Materials and Methods, Figures, Data Availability Section)
If study protocol has been **pre-registered, provide DOI in the manuscript**. For clinical trials, provide the trial registration number **OR** cite DOI.	Not Applicable	
Report the **clinical trial registration number** (at ClinicalTrials.gov or equivalent), where applicable.	Not Applicable	

Laboratory protocol	Information included in the manuscript?	In which section is the information available? (Reagents and Tools Table, Materials and Methods, Figures, Data Availability Section)
Provide DOI OR other citation details if **external detailed step-by-step protocols** are available.	Not Applicable	

Experimental study design and statistics	Information included in the manuscript?	In which section is the information available? (Reagents and Tools Table, Materials and Methods, Figures, Data Availability Section)
Include a statement about **sample size** estimate even if no statistical methods were used.	Not Applicable	
Were any steps taken to minimize the effects of subjective bias when allocating animals/samples to treatment (e.g. **randomization procedure**)? If yes, have they been described?	Not Applicable	
Include a statement about **blinding** even if no blinding was done.	Not Applicable	
Describe **inclusion/exclusion criteria** if samples or animals were excluded from the analysis. Were the criteria pre-established?<br><br>If sample or data points were omitted from analysis, report if this was due to attrition or intentional exclusion and provide justification.	Not Applicable	
For every figure, are **statistical tests** justified as appropriate? Do the data meet the assumptions of the tests (e.g., normal distribution)? Describe any methods used to assess it. Is there an estimate of variation within each group of data? Is the variance similar between the groups that are being statistically compared?	Yes	Figures

Sample definition and in-laboratory replication	Information included in the manuscript?	In which section is the information available? (Reagents and Tools Table, Materials and Methods, Figures, Data Availability Section)
In the figure legends: state number of times the experiment was **replicated** in laboratory.	Yes	Figures
In the figure legends: define whether data describe **technical or biological replicates**.	Yes	Figures

Ethics

Ethics	Information included in the manuscript?	In which section is the information available? (Reagents and Tools Table, Materials and Methods, Figures, Data Availability Section)
Studies involving **human participants**: State details of **authority granting ethics approval** (IRB or equivalent committee(s), provide reference number for approval.	Not Applicable	
Studies involving **human participants**: Include a statement confirming that **informed consent** was obtained from all subjects and that the experiments conformed to the principles set out in the WMA Declaration of Helsinki and the Department of Health and Human Services Belmont Report.	Not Applicable	
Studies involving **human participants:** For publication of **patient photos**, include a statement confirming that consent to publish was obtained.	Not Applicable	
Studies involving experimental **animals**: State details of **authority granting ethics approva**l (IRB or equivalent committee(s), provide reference number for approval. Include a statement of compliance with ethical regulations.	Not Applicable	
Studies involving **specimen and field samples:** State if relevant **permits** obtained, provide details of authority approving study; if none were required, explain why.	Not Applicable	

Dual Use Research of Concern (DURC)	Information included in the manuscript?	In which section is the information available? (Reagents and Tools Table, Materials and Methods, Figures, Data Availability Section)
Could your study fall under dual use research restrictions? Please check biosecurity documents and list of **select agents and toxins** (CDC): https://www.selectagents.gov/sat/list.htm	Not Applicable	
If you used a select agent, is the security level of the lab appropriate and reported in the manuscript?	Not Applicable	
If a study is subject to dual use research of concern regulations, is the name of the **authority granting approval and reference number** for the regulatory approval provided in the manuscript?	Not Applicable	

Reporting

The MDAR framework recommends adoption of discipline-specific guidelines, established and endorsed through community initiatives. Journals have their own policy about requiring specific guidelines and recommendations to complement MDAR.

Adherence to community standards	Information included in the manuscript?	In which section is the information available? (Reagents and Tools Table, Materials and Methods, Figures, Data Availability Section)
State if relevant guidelines or checklists (e.g., **ICMJE, MIBBI, ARRIVE, PRISMA**) have been followed or provided.	Not Applicable	
For **tumor marker prognostic studies,** we recommend that you follow the **REMARK** reporting guidelines (see link list at top right). See author guidelines, under 'Reporting Guidelines'. Please confirm you have followed these guidelines.	Not Applicable	
For **phase II and III randomized controlled trials**, please refer to the **CONSORT** flow diagram (see link list at top right) and submit the CONSORT checklist (see link list at top right) with your submission. See author guidelines, under 'Reporting Guidelines'. Please confirm you have submitted this list.	Not Applicable	

Data Availability

Data availability	Information included in the manuscript?	In which section is the information available? (Reagents and Tools Table, Materials and Methods, Figures, Data Availability Section)
Have **primary datasets** been deposited according to the journal's guidelines (see 'Data Deposition' section) and the respective accession numbers provided in the Data Availability Section?	Not Applicable	
Were **human clinical and genomic datasets** deposited in a public access-controlled repository in accordance to ethical obligations to the patients and to the applicable consent agreement?	Not Applicable	
Are **computational models** that are central and integral to a study available without restrictions in a machine-readable form? Were the relevant accession numbers or links provided?	Not Applicable	
If publicly available data were reused, provide the respective **data citations in the reference list**.	Not Applicable	
